# Heteroatom Effects on Quantum Interference in Molecular Junctions: Exploring Perturbation through Multiple Cross-Conjugation

**DOI:** 10.1021/acs.jpcc.5c08386

**Published:** 2026-04-02

**Authors:** Luke J. O’Driscoll, James M. Targett, Wei Xu, Rebecca J. Salthouse, Luke J. Williams, Abdalghani Daaoub, Sara Sangtarash, Wenjing Hong, Hatef Sadeghi, Martin R. Bryce

**Affiliations:** † Department of Chemistry, 3057 Durham University, Lower Mountjoy, Stockton Road, Durham DH1 3LE, U.K.; ‡ Quantum Device Modelling Group, School of Engineering, 2707 University of Warwick, Coventry CV4 7AL, U.K.; § State Key Laboratory of Physical Chemistry of Solid Surfaces, College of Chemistry and Chemical Engineering & IKKEM, 12466 Xiamen University, 361005 Xiamen, China

## Abstract

Understanding how quantum interference (QI) controls charge transport through metal | molecule | metal junctions is central to the progress of molecular electronics. In this work, scanning tunneling microscopy break junction (STM-BJ) measurements combined with charge transport calculations have been used to investigate, for the first time, the conductance of single-molecules that combine (i) multiple cross-conjugation points, (ii) nonalternant frameworks, and (iii) heteroatoms in the conductance pathway. The six studied molecules are symmetrical triaryl systems, each containing two terminal 1,3-difunctionalized pyrrole rings. Their structures vary in three aspects: (i) the anchoring groups (SMe or thiolate), (ii) the connectivity (*para* or *meta* central ring), and (iii) whether the central ring is benzene or pyridine. STM-BJ studies showed that *para*-connectivity afforded higher conductance than *meta*-connectivity, but in contrast to analogous conjugated systems, there was no appreciable difference between *meta*-connected benzene and pyridine species. These results offer an advanced testbed for QI interpretation via intuitive curly arrow rules, orbital analysis, “M-theory”, and high-level computational techniques. By combining these methods to account for subtle modulation of destructive QI and antiresonances, the experimental trends have been successfully rationalized. These fundamental experimental and computational insights should be applicable to other heterocycles in molecular junctions.

## Graphical abstract

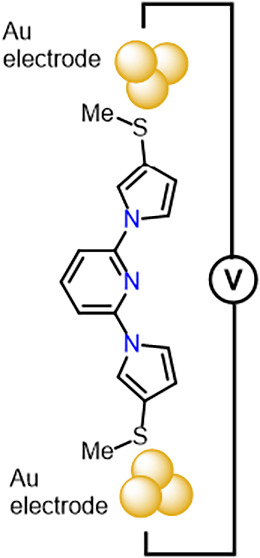

## Introduction

Since the advent of research into the conductance of molecular junctions in the late 1990s and early 2000s, the refinement of scanning tunnelling microscopy break junction (STM-BJ)^1^ and mechanically controlled break junction (MCBJ)^2^ techniques has enabled the single-molecule conductance of a diverse range of molecules to be determined experimentally.^3–8^ Key structure–property relationships have emerged and strategies to better understand, predict and optimize different aspects of transport behavior have been proposed and verified.^9–19^

Many of the structure–property relationships in molecular wires can be attributed to quantum interference (QI) effects. The nanoscale dimensions of molecular wires mean that electrons traveling between electrodes through these structures can be considered as de Broglie waves which can form either constructive or destructive interference patterns.^20^ Destructive QI (DQI) results in reduced electronic conductance compared to constructive QI (CQI).^21^ In contrast, DQI can result in enhanced thermopower (i.e., higher Seebeck coefficient) relative to CQI.^22,23^ Understanding how structural variations of molecular wires can influence QI, and hence tune charge transport behavior, is highly relevant to proposed applications in energy conversion.^24^

The archetypal QI effect is the lower conductance of *meta*-functionalized benzene derivatives versus *para*-isomers.^14,21^ Comparable behavior related to connectivity is observed or predicted for more complex species.^11,25–27^ Many methods have been developed to predict and rationalize QI behavior, particularly in alternant hydrocarbons, without having to resort to computationally demanding charge transport simulations. These include curly arrow rules^11,26^ and other graphical schemes,^28,29^ approaches derived from graph theory,^27,30,31^ magic ratio rules and “M-theory”,^10,32,33^ and molecular orbital analysis, including the use of “QI maps”.^13,34,35^ In summary, for alternant hydrocarbons, CQI is expected for molecules with full conjugation between their anchoring groups, whereas in the absence of full conjugation DQI is expected.

The effects of structural features such as cross-conjugation, nonalternant frameworks and heteroatoms on QI are more challenging to summarize succinctly, especially if these factors are combined in the same molecule. Several recent experimental and theoretical studies have probed the effect of heteroatoms on QI behavior.^12,18,19,25,30,33,36–42^ Notably, heteroatoms can cause QI behavior to differ from that observed in analogous hydrocarbon systems. Often this is associated with a shift in the position of the DQI feature away from the Fermi energy (*E_F_*) in transmission functions derived from charge transport simulations. In many cases, this “shifted DQI” (SDQI) can be predicted and rationalized using “extended curly arrow rules” (ECARs).^11^ These rules state that where DQI is expected because conjugation is not possible between the anchoring groups of a molecular wire, SDQI is expected if both anchoring groups can be conjugated to the same electron-withdrawing or -donating group (typically a cross-conjugated heteroatom).

In this context we investigated the QI behavior of molecular wires based on 1-phenylpyrrole systems **A** and **B** (Figure 1),^12^ which combine a nonalternant structure, a heteroatom and cross-conjugation. The 1-phenylpyrrole motif meant that any π-orbital-based conductance pathway was required to pass through a heteroatom p-orbital (i.e., that formally containing the nitrogen lone pair). This lone pair can be delocalized either to an acceptor substituent on the pyrrole ring or to an acceptor substituent in the *para-*position on the phenyl ring, such that SDQI is expected according to ECARs.^11^ For a *meta-*substituent on the phenyl ring, such delocalization is not possible and DQI was predicted. The corresponding trends in the conductance behavior were indeed observed experimentally.^12^ Interestingly, despite the presence of cross-conjugation, the conductance of **A**, containing a 1,3-difunctionalized pyrrole, was comparable to that of the *para*-substituted benzene analog **C** (Figure 1) and higher than its *meta*-substituted benzene analog **D**. Furthermore, the conductance of pyrrole derivative **B** was intermediate between that of its *para-* and *meta*-substituted benzene analogs (**D** and **E**, respectively, Figure 1).^12^ The 1,3-difunctionalized pyrrole motif is therefore of interest in achieving relatively high conductance despite SDQI.

**Figure 1:**
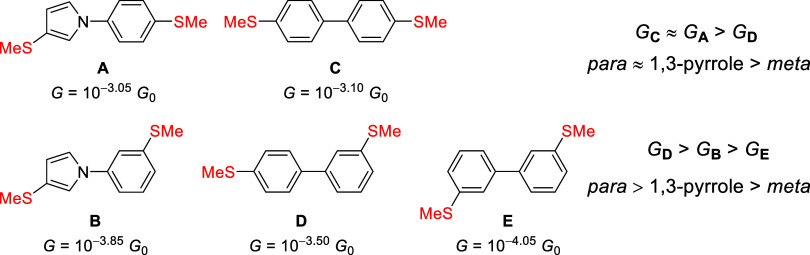
Summary of a previous study of 1-phenylpyrrole molecular wires, highlighting the observed conductance trends with respect to biphenyl analogues.^12^ Species **C**, **A** and **D** represent a variation of one ring from *para*-benzene to 1,3-disubstituted pyrrole to *meta*-benzene, respectively, while the second ring remains *para*-benzene. Species **D**, **B** and **E** represent variation of one ring from *para*-benzene to 1,3-disubstituted pyrrole to *meta*-benzene, respectively, while the second ring remains *meta*-benzene.

The present experimental and theoretical work extends the study of molecules **A** and **B** outlined above.^12^ To our knowledge, this is the first QI study of molecular wires which combine multiple heteroatoms in the conduction pathway, cross-conjugation and a nonalternant framework. The investigated species (**1-SMe**, **2-SMe**, **3-SMe**, **1-SAc**, **2-SAc** and **3-SAc**: Figure 2) comprise symmetrical triaryl systems each containing two 1,3-difunctionalized pyrrole rings. The molecules differ from one another in three respects: (i) their connectivity, (ii) their anchoring groups (SMe or thiolate), and (iii) whether the central ring is benzene or pyridine. This combination of factors allows the interaction of several previously reported QI and charge transport phenomena to be investigated in relatively simple systems.

**Figure 2:**
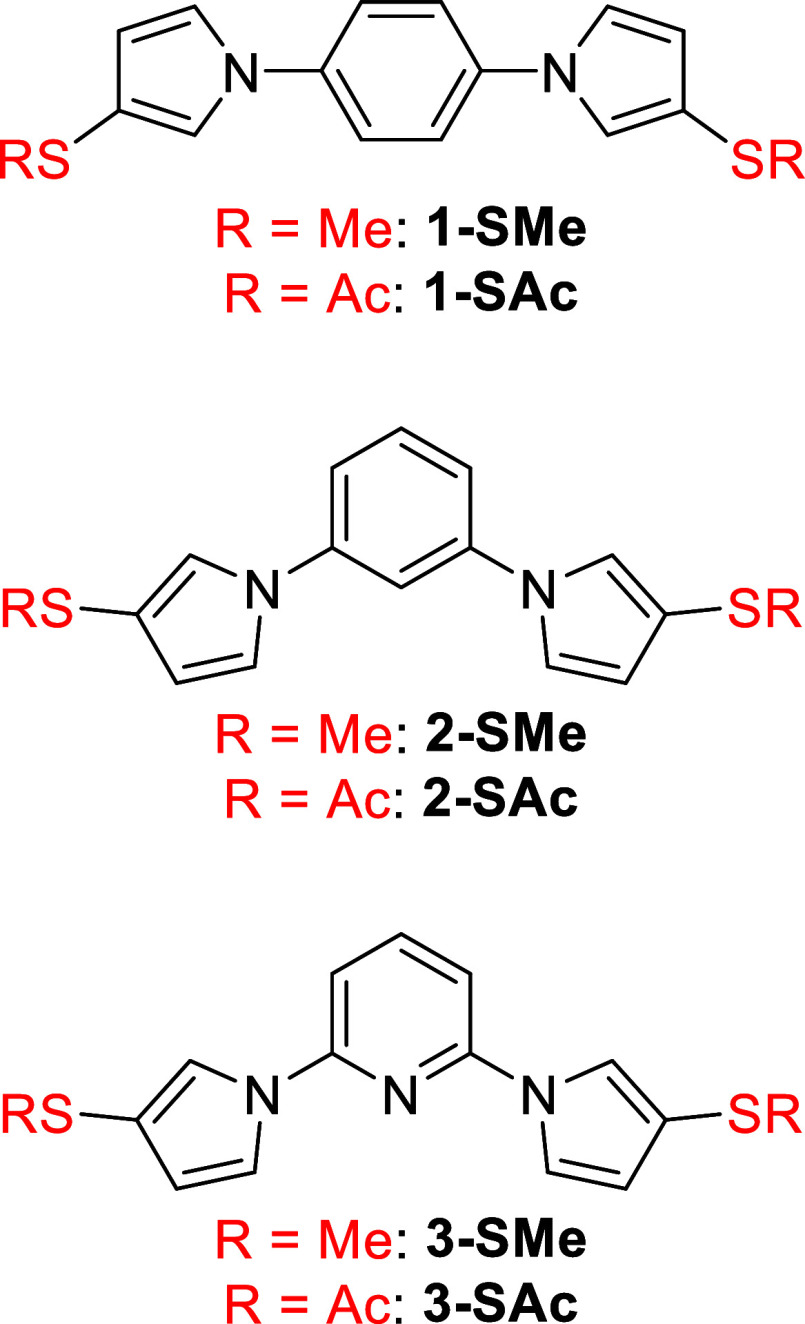
Structures of the six triaryl molecular wires **1-SMe**, **2-SMe**, **3-SMe**, **1-SAc**, **2-SAc** and **3-SAc** studied in the present work. Anchoring groups are indicated in red.

## Methods

### Synthesis and Characterization

Full synthetic details and characterization of **1-SMe**, **2-SMe**, **3-SMe**, **1-SAc**, **2-SAc** and **3-SAc** and their precursors are reported in the Supporting Information (SI). The general protocol for the synthesis of the thiomethyl anchored species (**1-SMe**, **2-SMe** and **3-SMe**) was based on that previously used to prepare **A** and **B**.^12^ 3-Bromo-1-(triisopropylsilyl)­pyrrole was converted to 3-(methylthio)-1-(triisopropylsilyl)­pyrrole via lithiation and treatment with dimethyl disulfide.^43^ In a one-pot procedure, removal of the silyl protecting group using tetrabutylammonium fluoride (TBAF) was followed by Ullmann coupling with the relevant dihaloarene^44^ to afford the target molecules.

The synthesis of the (protected) thiol anchored species (**1-SAc**, **2-SAc** and **3-SAc**) required a longer route. 3-Bromo-1-(triisopropylsilyl)­pyrrole was desilylated using TBAF then reacted with either the appropriate diiodobenzene in an Ullmann coupling^44^ or with 2,6-difluoropyridine in a nucleophilic aromatic substitution reaction.^43^ The resulting dibromotriarene was then converted to the diiodide via lithium-halogen exchange followed by treatment with iodine solution. Finally, the diiodides were converted to dithioacetates using a Cu-catalyzed C–S coupling reaction with potassium thioacetate.^45^

### Molecular Conductance Measurements

Molecular conductance measurements were performed using the STM-BJ technique using lab-built equipment as described previously.^1,12,46^ In brief, molecular junctions were repeatedly formed by driving the gold tip in and out of contact with a gold substrate. Conductance was measured as a function of the gold tip–substrate displacement, which is mainly controlled by a piezo stack during the repeated formation of junctions (see SI Section 3 for more details). All experiments were carried out in a solution of the target molecules (0.1 mM) in mesitylene or trichlorobenzene under ambient conditions with a 0.1 V bias voltage. Logarithmically binned one-dimensional (1D) conductance histograms and two-dimensional (2D) conductance-displacement histograms were plotted by compiling at least 2000 molecular conductance-displacement traces. Statistical analysis was performed using the methods reported previously.^46^

### Charge Transport Simulations

To investigate the electronic properties of the junctions, the mean-field Hamiltonian of each structure was obtained from the optimized geometry of the junctions using density functional theory (DFT; see SI Section 4.3).^47^ These Hamiltonians were then combined with the transport code Gollum^48,49^ to calculate the electron transmission coefficient *T*(*E*) with energy *E* passing from one gold electrode to the other. The conductance is proportional to *T*(*E*) via the Landauer formula $G=G_{0}\int d\mathrm{ET}\left(E\right)\left(-\frac{\partial f}{\partial E}\right)$ where *G*_0_ is conductance quantum, $f\left(E\right)={\left(1+\exp\left(\frac{\left(E-E_{F}\right)}{k_{B}T}\right)\right)}^{-1}$ is the Fermi function, *k*_B_ is the Boltzmann’s constant, *T* is the temperature, and *E*_*F*_ is the Fermi energy of the electrode (see SI Section 4.4).

## Results and Discussion

### Molecular Design Rationale

The six molecules shown in Figure 2 are all symmetrical, with a central aromatic ring bound to two pyrrole rings through their nitrogen atoms. Each pyrrole ring bears an anchoring group in the 3-position. This design means that any π-orbital-based conductance pathway between the anchoring groups must pass through at least two nitrogen p-orbitals; no all-carbon p-orbital pathway between the anchoring groups exists. All these molecules are therefore multiply cross-conjugated, with each pyrrole nitrogen conjugated to the central ring and to one of the two anchoring groups. This has the consequence that they are all expected to show DQI based on ECARs,^11^ as there is neither conjugation between the two anchoring groups nor conjugation of both anchoring groups to the same electron-withdrawing or -donating group. An ECARs analysis of the studied systems, and consideration of curly arrow interactions beyond ECARs, is presented in the SI (Section 1.1, Figures S1–S3).

Three different central rings were studied (*para-*benzene, **1**; *meta-*benzene, **2**; and 2,6-disubstituted pyridine, **3**), each with thiomethyl (**SMe**) and (protected) thiol anchoring groups (**SAc**). The acetyl protecting group undergoes *in situ* cleavage to form thiolate-anchored assemblies on gold and in gold | molecule | gold junctions.^50–52^ The thiolate species derived from the **SAc** series will be referred to as **1-S**, **2-S** and **3-S**.

These structures allow the interplay of a range of QI and charge transport phenomena to be investigated. The different QI behavior of *para*- (CQI) and *meta*- (DQI) substituted benzenes is well-established.^14,21,22,36,40,53,54^ Comparison between backbones **1** and **2** enables any effect related to the inclusion of two cross-conjugated heteroatoms in the π-conductance pathway to be observed, especially as the conductance of the *para-*anchored *N*-phenylpyrrole molecule **A** was comparable to the *para-*anchored biphenyl analog **C**.^12^

Several experimental^36,37,40,55^ and theoretical^33,38,39^ works have shown that “perturbing” a *meta*-connected benzene ring (or a polycyclic aromatic hydrocarbon) within a molecular wire can result in significantly different QI behavior versus the parent system. This perturbation can be accomplished either by adding an electron-donating or -withdrawing substituent to the benzene ring, or by replacing benzene with a *meta*-connected 6-membered heteroaromatic ring such as pyridine. In the present family of molecules the 2,6-disubstituted pyridine in **3-SMe** and **3-SAc** was expected to change the QI behavior relative to **2-SMe** and **2-SAc** based on prior studies of different molecular backbones.^36–39^ 2,6-Disubstitution was favored over 2,4-disubstitution to retain a simple, symmetrical system. Moreover, the increased steric hindrance around the pyridyl nitrogen atom in **3-SMe** and **3-SAc**, relative to their 2,4-isomers, should restrict the pyridyl nitrogen atoms from binding to an electrode, which would create undesired shorter conductance pathways.^55,56^ The 3,5-disubstitued analog was not considered as this perturbation previously had no effect on QI behavior.^36,38–40^

Investigating whether the anchor group influenced the observed QI behavior was also of interest. Thiomethyl (**SMe**) groups form dative bonds to Au surfaces whereas thiolates (**S**) can bind covalently.^50–52^ Furthermore, several prior studies have compared the Seebeck coefficient of otherwise equivalent **SMe**- and **S**-anchored molecular wires.^57–59^ Thiolate anchors consistently afford positive Seebeck coefficients,^57,58,60^ indicative of HOMO-dominated conductance (i.e., hole transport dominates). In contrast, thiomethyl anchors often (although not always^59,61^ ) have negative Seebeck coefficients,^57,58,62,63^ indicative of LUMO-dominated conductance (i.e., electron transport dominates). These two anchoring groups should enable comparisons between different binding modes and potentially different dominant transport orbitals to establish whether QI behavior is consistent for the molecular backbones. The chosen anchors made the molecules synthetically accessible and structurally similar, reducing the impact of other variables on their properties.

### Molecular Conductance Studies

The most probable molecular conductances for the six molecular wires were determined using the STM-BJ technique.^1,46^ The results are summarized in Table 1 with the 1D and 2D conductance histograms for the ***n*** **-S** series (***n*** = **1**, **2**, or **3**) shown in Figure 3. Conductance traces for the ***n*** **-SMe** series are shown in Figure S24. Two trends are clear: (i) for both the **SMe** and **S** series the *para*-connected benzene species (**1-X**; **X** = **SMe** or **S**) are significantly more conductive than their *meta-*isomers (**2-X**); (ii) the thiolate species (***n*** **-S**) are consistently more conductive than their thiomethyl analogs (***n*** **-SMe**). These results are broadly in line with prior studies which compared *para-* and *meta*-connectivity,^14,21,22,36,40,53,54^ and **S** versus **SMe** anchors in different series of molecular wires.^57–59,64,65^ The experimental plateau lengths and the DFT-optimized intramolecular Au—Au distances are reported in SI Section 3.1 and Figures S25 and S31. A comparison of the experimental and theoretical junction lengths reveals consistent trends, with the theoretical values remaining consistently higher. This can be attributed to the theory representing fully stretched junctions as opposed to the experimental average configuration.

**Figure 3:**
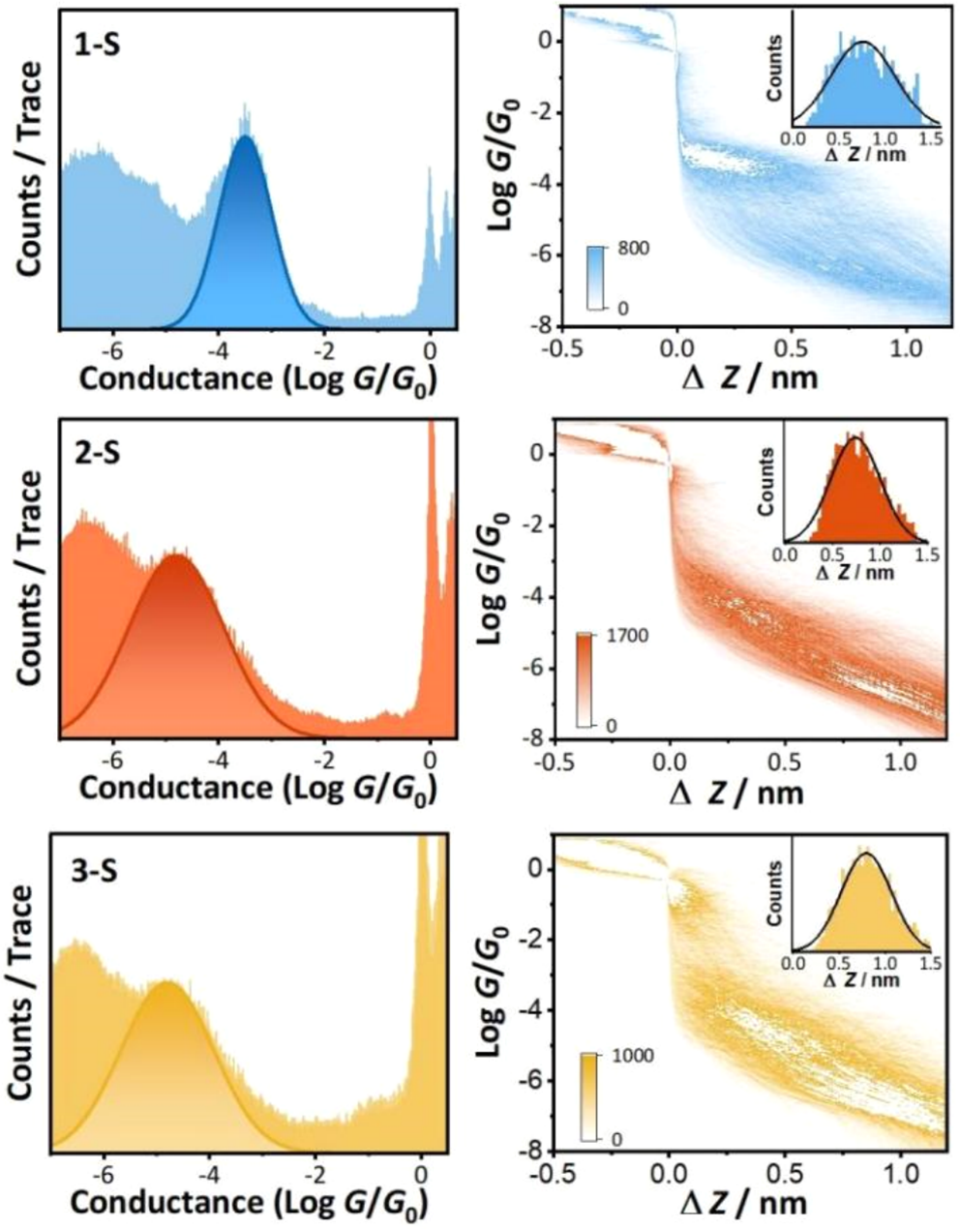
Left: Conductance histograms obtained for **1-S**, **2-S** and **3-S** in trichlorobenzene, with the most probable conductance peak indicated with a Gaussian fit. Right: 2D conductance-displacement histograms for **1-S**, **2-S** and **3-S**.

**Table 1:** Summary of Experimental and Computational Conductance Data for the Investigated Species

molecule	STM-BJ conductance (log (*G*/*G*_0_))	charge transport simulation conductance at *E_F_* = 0.5 eV (log (*G*/*G*_0_))
1-SMe	–3.96^a^; −3.92^b^	–3.02
2-SMe	–4.91^a^; −4.91^b^	–4.48
3-SMe	–4.91^a^; −4.86^b^	–4.48
1-S	–3.50^a^	–3.41
2-S	–4.77^a^	–4.31
3-S	–4.78^a^	–3.80

a Solvent: Trichlorobenzene (TCB).

b Solvent: Trimethylbenzene (TMB, mesitylene).

For both **SMe** and **S** anchors, the conductance of the *para*-connected species (**1-X**) is approximately a factor of 2.5 less than literature values for analogous *para*-terphenyls with anchors in the *para*-positions. **1-SMe** (10^–3.92^ *G*_0_ in TMB) can be compared directly to 4,4″-bis­(methylthio)-*p*-terphenyl measured under equivalent conditions in the same laboratory (10^–3.56^ *G*_0_ in TMB);^46^ other studies report 10^–3.65^ *G*_0_ ^66^ and 10^–3.1^ *G*_0_ ^65^ in TCB. For **1-S**, a conductance of 10^–3.50^ *G*_0_ was recorded in TCB which can be compared to reported values for *p*-terphenyl-4,4″-dithiolate of 10^–2.7^ *G*_0_ in TCB^65^ and 10^–3.15^ *G*_0_ ^67^ or ca. 10^–3.2^ *G*_0_ ^68^ under solvent-free conditions. This is a relatively modest decrease compared to when the anchoring position on two terminal benzene rings is changed from *para* to *meta*, which is typically at least an order of magnitude decrease.^14,25,69,70^ These observations provide further evidence that in molecular wires 1,3-difunctionalized pyrrole units represent an intermediate between benzene rings bearing anchors in the *para*- and *meta*-positions.^12^ Flicker noise measurements indicate that through-bond is the predominant charge transport pathway in the present systems (Figure S26).

In contrast to expectations based on previous studies,^36–39^ no significant difference was observed when comparing *meta*-benzene species (**2-X**) with their 2,6-disubstituted pyridine analogs (**3-X**) for either anchoring group. This poses the question of whether the pyrrole units have enhanced the conductance of **2-X** or suppressed the conductance of **3-X**, relative to analogous systems. A quantum circuit rule has previously shown that the contribution of the central ring of oligo­(arylene ethynylene) (OAE) wires comprising three alkyne-linked 6-membered benzene or pyridine rings is dominant over, and largely independent of, anchoring group contributions to molecular conductance.^14^ The rule states that

$$\frac{G_{A1‐p‐A1}}{G_{A1‐m‐A1}}\approx \frac{G_{A2‐p‐A2}}{G_{A2‐m‐A2}}$$

where *G*_A*x*‑c‑A*x*_ is the conductance of a molecular wire in which two anchor groups *A*_*x*_ are connected to a central ring with connectivity c (i.e., *para* = *p* or *meta* = *m*).

By extending this rule to systems beyond those in the initial study, including triaryls, it is possible to investigate whether the present molecules behave like *meta*-benzene derivatives or 2,6-pyridyl derivatives. Table 2 compares the ratios between the conductances of *para*-benzene connected systems (*G*_*para*_) and either *meta*-benzene connected analogs (*G*_*meta*_) or perturbed *meta*-analogs based on 2,6-difunctionalized pyridines (*G*_2,6‑py_) for the present species and other systems reported in the literature (the structures of these systems are summarized in Table S1 in the SI). If the quantum circuit rule^14^ holds more generally, *G*_*para*_/*G*_*meta*_ (and *G*_*para*_/*G*_2,6‑py_) should be comparable across the different systems, although a broad range of values may be anticipated given the inherent width of measured conductance distributions. Examples for *G*_*para*_/*G*_2,6‑py_ are limited to two prior studies, but a value close to 10 is obtained in both cases. Across a wider range of examples, *G*_*para*_/*G*_*meta*_ usually lies between ca. 20 and 45, although there are exceptions. These include an unusually low *G*_*para*_/*G*_*meta*_ of 3.3 for iodo-anchored terphenyls,^54^ although it is notable that in this gating study the ungated conductance values affording this ratio were found to lie at a bias far from that needed to observe significant DQI effects. This may relate to the unusual choice of an iodide anchoring group. The value obtained for thiolate-anchored OAEs in work by Miao et al.^22^ (*G*_*para*_/*G*_*meta*_ = 10.9) differs significantly from that of Liu et al.^36^ for the same compounds (*G*_*para*_/*G*_*meta*_ = 31.6). While the conductance of the *para*-isomer shows good agreement across both studies, the conductance of the *meta-*isomer reported by Miao et al.^22^ is higher than that reported by Liu et al.^36^ for the same system and for a 3,5-pyridyl analog (i.e., a heteroatom in a nonperturbing position), and that reported for a *meta*-methoxy-substituted analog^40^ (i.e., substitution in a nonperturbing position). We therefore consider the value of 31.6 derived from Liu et al.’s study to be more reliable. Other exceptions relate to another 5-membered heterocycle, oxazole, as discussed further below. The large variability in Table 2 demonstrates the need for further studies to provide more reliable data for the application of quantum circuit rules.

**Table 2:** Comparison of Experimental Conductance Ratios for Analogous Para and Meta Connected Molecular Wires, and Where Applicable, 2,6-Pyridyl Connected Molecular Wires

system	*G_para_*/*G_meta_*	*G*_*para*_/*G*_2,6‑py_
**1-SMe**, **2-SMe**, **3-SMe** (this work)	8.9^a^; 9.8^b^	8.9^a^; 8.7^b^
**1-S**, **2-S**, **3-S** (this work)	18.6^a^	19.1^a^
thiolate-anchored OAEs (ref 36)	31.6	12.6
thiomethyl-anchored terphenyls and derivatives^c^ (refs 37,46 )	43.7^c^	6.9
thiolate-anchored OAEs (ref 22)	10.9	
*para*-pyridyl-anchored OAEs (MCBJ) (ref 14)	31.6	
*para*-pyridyl-anchored OAEs (STM-BJ) (ref 14)	20.0	
*meta*-pyridyl anchored OAEs (ref 14)	25.1	
DHBT-anchored OAEs^d^ (ref 53)	>79.4^e^	
iodo-anchored terphenyls (ref 54)	3.3	
5-oxazolyl-anchored triaryls (ref 71)	6.2	
4-oxazolyl-anchored triaryls (ref 71)	6.6	
asymmetric 4- and 5-oxazole-anchored triaryls (ref 71)	0.46	

a Solvent: Trichlorobenzene (TCB).

b Solvent: Trimethylbenzene (TMB, mesitylene).

c N.B. *G*_*para*_ derives from a different study to the other values and *G*_*meta*_ is derived from a more complex system that would be expected to afford comparable conductance to the simple *meta* analog of the other species.

d DHBT = dihydrobenzothiophene.

e A conductance was not detected above the experimental noise level for the *meta* species, but this still allows a minimum ratio to be determined based on the reported noise level.

For the present systems, unlike prior examples in the literature, *G*_*para*_/*G*_*meta*_ and *G*_*para*_/*G*_2,6‑py_ are approximately equal for a given anchoring group. For **SMe** anchors, *G*_*para*_/*G*_*meta*_ ≈ *G*_*para*_/*G*_2,6‑py_ ≈ 9, within the typical range of *G*_*para*_/*G*_2,6‑py_. This indicates that with **SMe** anchors, the inclusion of 1,3-difunctionalized pyrroles increased conductance in the *meta*-connected case, giving a value in line with expectations for a heteroatom-perturbed system. However, adding another heteroatom (i.e., the pyridine N) to further perturb the structure does not additionally enhance conductance. The anchor group appears to have some influence: for thiolate-anchored (***n*** **-S**) species, *G*_*para*_/*G*_*meta*_ ≈ *G*_*para*_/*G*_2,6‑py_ ≈ 19, i.e., at the lower end of the typical range for *G*_*para*_/*G*_*meta*_ and larger than other values of *G*_*para*_/*G*_2,6‑py_. This suggests that for the thiol anchor the opposite behavior is observed, i.e., the conductance of the 2,6-pyridyl system is suppressed to a value comparable to a *meta*-benzene system.

Heterocyclic perturbation of QI may not be restricted to pyrrole. Li et al. found that symmetrical oxazole-benzene-oxazole systems have a relatively low *G*_*para*_/*G*_*meta*_ of ca. 6, while unsymmetrical systems (in which two oxazoles were each connected through different positions) showed *G*_*para*_/*G*_*meta*_ = 0.46, i.e., the *meta*-isomer was (unusually) more conductive than the *para*.^71^ It was suggested that these observations may relate to either interacting DQI contributions, σ-conductance effects, or both. The low *G*_*para*_/*G*_*meta*_ for the symmetrical systems is similar to that determined for **1-SMe** vs **2-SMe**. This behavior may relate to the same QI phenomena, given that both studies (the present work and that of Li et al.^71^) include 5-membered heterocyclic anchor units, although in the oxazole systems an all-carbon p-orbital-based route between the anchoring units (the oxazole nitrogen atoms^71^) does exist.

Overall, the implication of these break junction data is that the conductance of the present systems, and potentially other heterocycle derivatives, is subject to subtle and underexplored QI effects. Notably, the presence of two 1,3-disubstituted pyrrole systems appears to perturb DQI in **2-SMe** comparably to heteroatom substitution on a central ring in analogous systems.

### QI Models and Charge Transport Simulations

To better understand the subtle QI effects in the present systems and their role in perturbation of DQI, computational studies were conducted. These included molecular orbital analysis and charge transport simulations based on both a simple tight-binding (TB) method and DFT material-specific Hamiltonians (Figure 4). Table S15 in the SI summarizes the QI and conductance behavior and trends predicted or measured using the different methods applied in this work.

**Figure 4:**
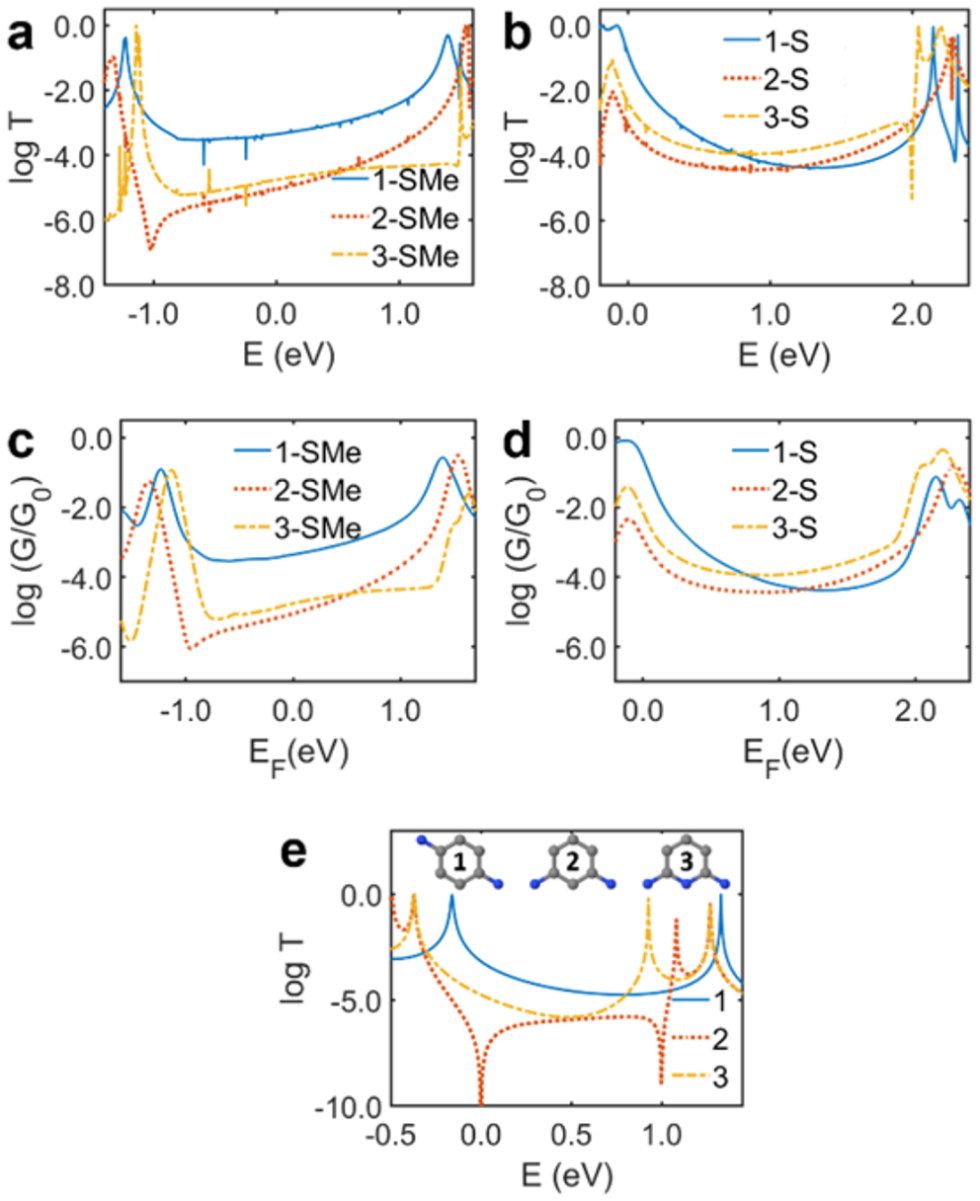
(a, b) DFT-derived transmission coefficient plots for electrons passing through molecular junctions (a: **1-SMe**, **2-SMe**, **3-SMe**; b: **1-S**, **2-S**, **3-S**) with gold electrodes, where *E* = 0 eV corresponds to the DFT Fermi energy. (c, d) Conductance spectra as a function of Fermi level at room temperature (300 K), showing the average conductance of a given molecule (c: **1-SMe**, **2-SMe**, **3-SMe**; d: **1-S**, **2-S**, **3-S**), over several different configurations weighted by a Boltzmann factor. (e) Transmission functions for molecular cores **1–3** derived from simple TB models with all on-site energies and couplings set as ε_0_ = 0 and γ = – 1, respectively, except for the on-site energy of the nitrogen atoms which is ε_*N*_ = – 0.45.

DFT orbital analysis of the molecular cores and the six molecules containing anchor groups that were measured in the STM-BJ experiments is presented in SI Section 4.1 and Figures S27–S28. Magic ratio rules and M-theory analysis are presented in SI Section 4.2, Figures S29–S30 and Tables S2–S14. DFT electron transmission *T*(*E*) plots were calculated for all six molecules in optimized configurations between gold electrodes (Figure S31 in the SI) based on DFT material-specific Hamiltonians. The resulting plots are shown in Figure 4a (**SMe** anchors) and Figure 4b (**S** anchors) with equivalent plots grouped by molecular backbone shown in Figure S34 in the SI. Figure 4a shows that **1-SMe** has the highest transmission of the **SMe**-anchored series at the DFT Fermi level (*E_F_*, i.e., at *E* = 0 eV), and indeed, across the HOMO–LUMO gap. The shape of the transmission function for **1-SMe** is indicative of CQI. **2-SMe** and **3-SMe** have similar transmission at the DFT Fermi level. The transmission function for **2-SMe** has a clear DQI feature at around *E* = ca. −1 eV, i.e., shifted away from *E_F_*, which resembles the SDQI behavior predicted for simpler systems using ECARs.^11^ The form of the transmission function for **3-SMe** is more complex, with no clear DQI antiresonance dip. The sharp change in the conductance for **3-SMe** in Figure 4c is attributed to the broadening of the degenerate HOMO resonance.

**1-S** has substantially higher transmission than **2-S** and **3-S** close to *E_F_* and its transmission remains higher for *E* < ca. 0.75 eV (Figure 4b). As for **1-SMe**, the shape of the transmission function for **1-S** is indicative of CQI. A clear antiresonance feature is seen at *E* = ca. 2 eV (again aligning with SDQI behavior as this is far from *E_F_*) for **3-S**, but interestingly, and in contrast to **2-SMe**, the curve for **2-S** shows no obvious DQI features. The possibility of a CQI-type curve for **2-SH** (a neutral model for **2-S**) due to orbital degeneracy was noted in the molecular orbital analysis (SI section 4.1). The energy difference between the LUMO+1 and LUMO of **2-SH** (0.02 eV) is smaller than for **3-SH** (0.12 eV). Therefore, the contribution from LUMO+1 to transport is expected to be larger in **2-SH** than **3-SH.** From the orbital rule (SI section 4.1), the LUMO+1 is expected to contribute to transport constructively, and therefore, due to the substantial contribution of the LUMO+1 the antiresonance is washed out more in **2-S** compared to **3-S**.

The calculated conductance, *G*, derived from *T*(*E*) for each of the six molecular wires is shown in Figure 4c,d. The conductance of **1-SMe** is an order of magnitude higher than **2-SMe** and **3-SMe** for a wide range of *E*_*F*_ (±0.5 eV) around the DFT Fermi energy. Figure 4d shows the conductance of **1-S** is up to 1 order of magnitude higher than **2-S** and **3-S** for a wide energy range around and above the DFT Fermi energy with the best quantitative agreement with the experimental data near *E_F_* = 0.5 eV (Table 1). The conductance values determined using DFT calculations are slightly higher compared to the experimental measurements; this is due to well-known DFT limits that underestimate the HOMO–LUMO gap, leading to higher calculated conductances.^49^

The DFT-derived results were obtained using computationally intensive procedures based on first principles. To validate the observations from the DFT model via a less intensive method, a simple tight-binding (TB) model was used to model the molecular cores. The TB model utilized on-site energy *ε*_0_ = 0 and hopping integral γ = −1. The on-site energy of the nitrogen atoms was ε_*N*_ = −0.45. Figure 4e shows the resulting transmission *T*(*E*) plots, which indicate CQI for **1-X** and DQI for **2-X**, in good agreement with the orbital analysis. There is no clear antiresonance in the *T*(*E*) plot for **3-X**, but such a feature can be seen in an alternative model detailed in the SI which omits the contribution of the pyrrole nitrogen atoms (see SI Section 4.6 and Figure S34), suggesting that this feature is influenced by the adjoining heterocycles and may be shifted beyond the HOMO–LUMO gap when the N atoms are accounted for. The TB *T*(*E*) results show good agreement with corresponding DFT *T*(*E*) results between the HOMO and LUMO, with the only exception being **2-S**, where an antiresonance present in the TB plot is not observed in the DFT plot. However, as discussed above, the DFT-derived LUMO+1 and LUMO for **2-S** are close in energy which results in a washed out antiresonance feature, accounting for this discrepancy between the methods. Similar contributions from orbitals close in energy to the HOMO and LUMO are likely to account for other differences between the TB and DFT derived *T*(*E*) plots, such as the absence of sharp antiresonances in the DFT-derived transmission functions, as the antiresonance has been perturbed out of, or to the edge of the HOMO–LUMO gap (e.g., **2-SMe**). Overall, accounting for orbital degeneracy means that QI predictions from orbital analysis, M-theory, DFT *T*(*E*) and TB *T*(*E*) are all broadly in agreement. Based on a study of oxazole-terminated molecules with *meta* and *para*-phenylene cores,^71^ it is possible that a σ-channel could contribute to the charge transport in the *meta* systems **2** and **3**. However, due to the larger number of σ bonds in the present systems compared to systems in which this effect has been observed,^71,72^ this is unlikely and therefore further investigation was considered outside the scope of the present work. Discussion of how the experimental and the theoretical results may relate to ECARs is provided in SI Section 1.1.

## Conclusions

Six symmetrical triaryl molecular wires (**1-SMe**, **2-SMe**, **3-SMe**, **1-SAc**, **2-SAc** and **3-SAc**) comprising a central benzene or pyridine ring bound to two 1,3-difunctionalized pyrrole rings through the pyrrole nitrogen have been synthesized and characterized. These molecules were designed to enable investigation of the combined effect of multiple heteroatoms, cross-conjugation and a nonalternant framework on QI in single-molecule junctions. Molecular conductance studies showed that, as frequently seen in other systems, *para*-connectivity (**1-X**) affords higher molecular conductance than analogous *meta*-connectivity (**2-X**). In contrast to systems lacking multiple cross-conjugation,^36–39^ it was observed that perturbation of *meta*-connected species (**2-X**) by inclusion of a heteroatom in the central ring (**3-X**) did not significantly alter the molecular conductance. Qualitatively similar conductance trends were observed regardless of the anchoring group, but a more quantitative analysis based on a quantum circuit rule^14^ suggests there may be subtle differences in the QI behavior observed for the two different anchoring groups.

The applicability of various predictive models to these multifaceted systems has been probed and is summarized in Table S15 in the SI. Relatively straightforward ECARs^11^ cannot fully account for the behavior observed in these systems, but with further empirical data it may be possible to expand the rules to account for a wider range of structural motifs. Mathematically based M-theory^10,33^ performs better, correctly accounting for some key trends in the conductance of the systems. Orbital analysis,^49^ which requires computational determination of molecular orbitals, is also relatively successful as a predictive technique, albeit in this case interpretation is somewhat complicated owing to orbital degeneracy. Calculated transmission functions based on both DFT-derived Hamiltonians and a simple TB model show good agreement with each other (if orbital degeneracy is considered) and qualitative agreement with the experimental data. The resulting transmission plots can be used to further rationalize the less intensive predictive methods, such as orbital analysis.

The combined experimental and computational results emphasize the potential of 1,3-difunctionalized pyrrole units,^12^ and potentially other 5-membered heterocycles,^71,73^ as useful functional units to manipulate and better understand QI. Continued studies of heterocyclic, cross-conjugated, nonalternant systems are anticipated to improve understanding of QI behavior and allow for fine-tuning of properties relevant to molecular electronics, thermoelectrics, and beyond.

## Supplementary Material



## Data Availability

The data associated with this article is available in the manuscript and Supporting Information files.

## Notes

Experimental procedures, NMR spectra, UV–vis absorption spectra, conductance measurements, computational methods, and data (PDF)
