# Supplementary material for: Heteroatom Effects on Quantum Interference in Molecular Junctions: Exploring Perturbation through Multiple Cross-Conjugation
Source: J Phys Chem C Nanomater Interfaces. 2026 Apr 2;130(15):5610–8. doi: 10.1021/acs.jpcc.5c08386 (PMC13093477; doi:10.1021/acs.jpcc.5c08386)
Supplement: Supplementary file 1 [file jp5c08386_si_001.pdf]

SUPPORTING INFORMATION

Heteroatom Effects on Quantum Interference in Molecular Junctions: Exploring Perturbation through Multiple Cross-Conjugation

Luke J. O'Driscoll,[†]* James M. Targett,[‡] Wei Xu,[§] Rebecca J. Salthouse,[†] Luke J. Williams,[†] Abdalghani Daaoub,[‡] Sara Sangtarash,[‡] Wenjing Hong,[§,]* Hatef Sadeghi[‡,]* and Martin R. Bryce[†,]*

[†] Department of Chemistry, Durham University, Lower Mountjoy, Stockton Road, Durham, DH1 3LE, UK

[‡] Quantum Device Modelling Group, School of Engineering, University of Warwick, Coventry, CV4 7AL, UK

[§] State Key Laboratory of Physical Chemistry of Solid Surfaces, iChEM, NEL, College of Chemistry and Chemical Engineering, Xiamen University, Xiamen 361005, China

CONTENTS

1. SUPPLEMENTARY DISCUSSION 2
- 1.1: Extended Curly Arrow Rules analysis of investigated species 2
- 1.2: Literature Conductance Comparisons 5

2. SYNTHESIS AND CHARACTERIZATION 7
- 2.1: General experimental methods 7
- 2.2: Synthetic procedures 7
  - 2.2.1: Thiomethyl-anchored species 7
  - 2.2.2: Protected thiol-anchored species 10
- 2.3: ^{1}H NMR spectra of compounds used in conductance studies 18
- 2.4: ^{13}C NMR spectra of compounds used in conductance studies 24
- 2.5: HRMS Data 30
- 2.6: UV-visible spectra of molecular wires **1-SMe**, **2-SMe**, **3-SMe**, **1-SAc**, **2-SAc** and **3-SAc** 33

3. SINGLE MOLECULE CONDUCTANCE EXPERIMENTS 34
- 3.1: Experimental methods and discussion 34
- 3.2: Supplementary experimental figures 34

4. COMPUTATIONAL METHODS 37
- 4.1: Orbital analysis 37
  - 4.1.1: Basic equations 37
  - 4.1.2: DFT orbital analysis 37
- 4.2: Magic ratio rule and M-theory for heteroatoms 40
  - 4.2.1: Brief description 40
  - 4.2.2: 6-membered molecular core 40
  - 4.2.3: Complete molecules 42
- 4.3: Geometry optimization 53

4.4: Electron transport....53
4.5: Junction Schematics....54
4.6: DFT & TB Transmission Plots....57
5. SUMMARY....58
6. REFERENCES....59

1. SUPPLEMENTARY DISCUSSION

1.1: Extended Curly Arrow Rules analysis of investigated species

Extended curly arrow rules (ECARs) have been shown to enable QI behavior to be predicted using a straightforward method. The rules, as presented previously, [1] are:

ECAR-1. Identify the two anchoring units of a molecular wire and replace one with a donor group D and the other with an acceptor group A. If the D lone pair can be delocalized onto A using curly arrows, CQI is expected, if not DQI is expected.

ECAR-2. If DQI is expected, identify any electron-withdrawing groups (EWGs) or electron-donating groups (EDGs) present in the molecular wire. If EWGs are present, replace each anchor with D. If a lone pair from each D can be independently delocalized to the same EWG, DQI is expected to be shifted away from E_F (SDQI). If EDGs are present, replace each contact with A. If a lone pair (or negative charge) from the same EDG can be independently delocalized to each A, SDQI is expected. Otherwise, DQI is expected around E_F.

Figure S1-Figure S3 illustrate how ECARs can be applied to molecular wires of general structures **1-SR**, **2-SR** and **3-SR**. DQI is predicted in all cases, as it is not possible to either delocalize electrons from a D group at one anchoring site to an A at the other, nor to delocalize electrons from a pyrrole nitrogen lone pair (EDG) to both anchor groups of a given molecule. ECARs were developed empirically based on a variety of reported systems and are as such not necessarily exhaustive, as demonstrated in a recent study of antiaromatic species. [2] Given that multiple cross-conjugation, as seen in the present systems, has not been widely explored, it is of interest to see if other possible modes of curly arrow analysis can align with the trends observed in this work. Given the limited range of structures investigated, further studies would be required to draw confident conclusions. Nevertheless, the following can be noted:

1. In all systems, each pyrrole lone pair can donate to an acceptor group on the same pyrrole ring and into (but not across) the central aromatic ring. (The proposed donor-acceptor interactions between the pyrrole lone pairs and central rings are supported by trends observed in the UV-visible absorption spectra discussed in Section 2.5 below). Given the observed conductances and the absence of antiresonances near the center of the HOMO-LUMO gap (or E_F) in the DFT-derived transmission spectra, such delocalization may be sufficient to result in behavior other than DQI. However, this effect is not currently captured by ECARs. [1]
2. For **1-SR** systems, after delocalizing a pyrrole nitrogen lone pair to an acceptor group attached to the same pyrrole ring, it is then possible to independently delocalize the second pyrrole lone pair to both acceptor groups as shown in Figure S1 (c.f. ECAR-2). This is not possible for **2-SR** or **3-SR** (Figure S2 and Figure S3). Given the relatively high conductance of **1-SR** species, this possibility can tentatively be assigned as favoring increased conductance, potentially via CQI.
3. For **3-SR** systems, after delocalizing a pyrrole nitrogen lone pair to the pyridyl nitrogen on the central ring (an EWG), it is then possible to independently delocalize the second pyrrole lone

pair to both acceptor groups as shown in Figure S3 (c.f. ECAR-2). Given the similarities observed between **2-SR** and **3-SR**, it appears, based on the current data, that this possibility is not associated with increased conductance. It may, however, relate to the absence of a clear DQI antiresonance in the tight binding model for the molecular core of **3** (Section 4.6 below).

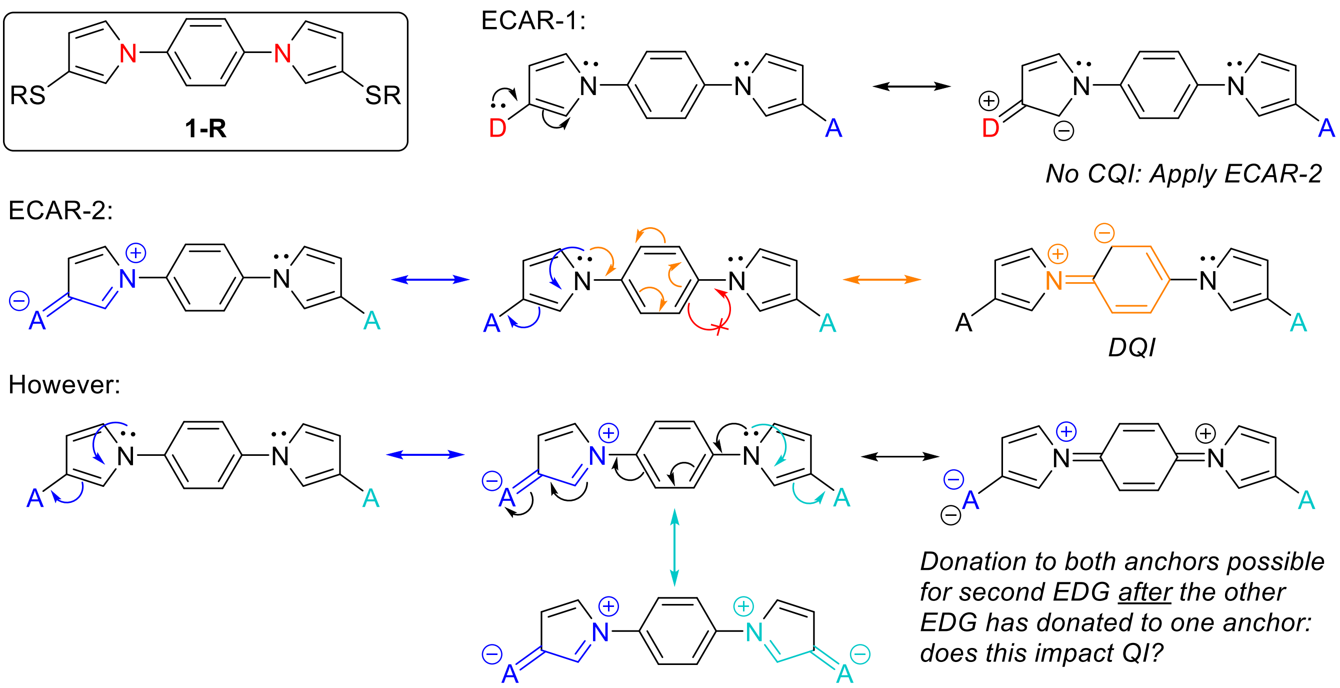

Figure S1. Analysis of the **1-SR** general molecular wire structure using ECARs, [1] and investigation of curly arrow interactions beyond ECARs for this system. Note that for ECAR-2, while it may appear that electrons can be delocalized onto a nitrogen bearing a lone pair, this is not possible as no vacant orbitals are available (red crossed curly arrow).

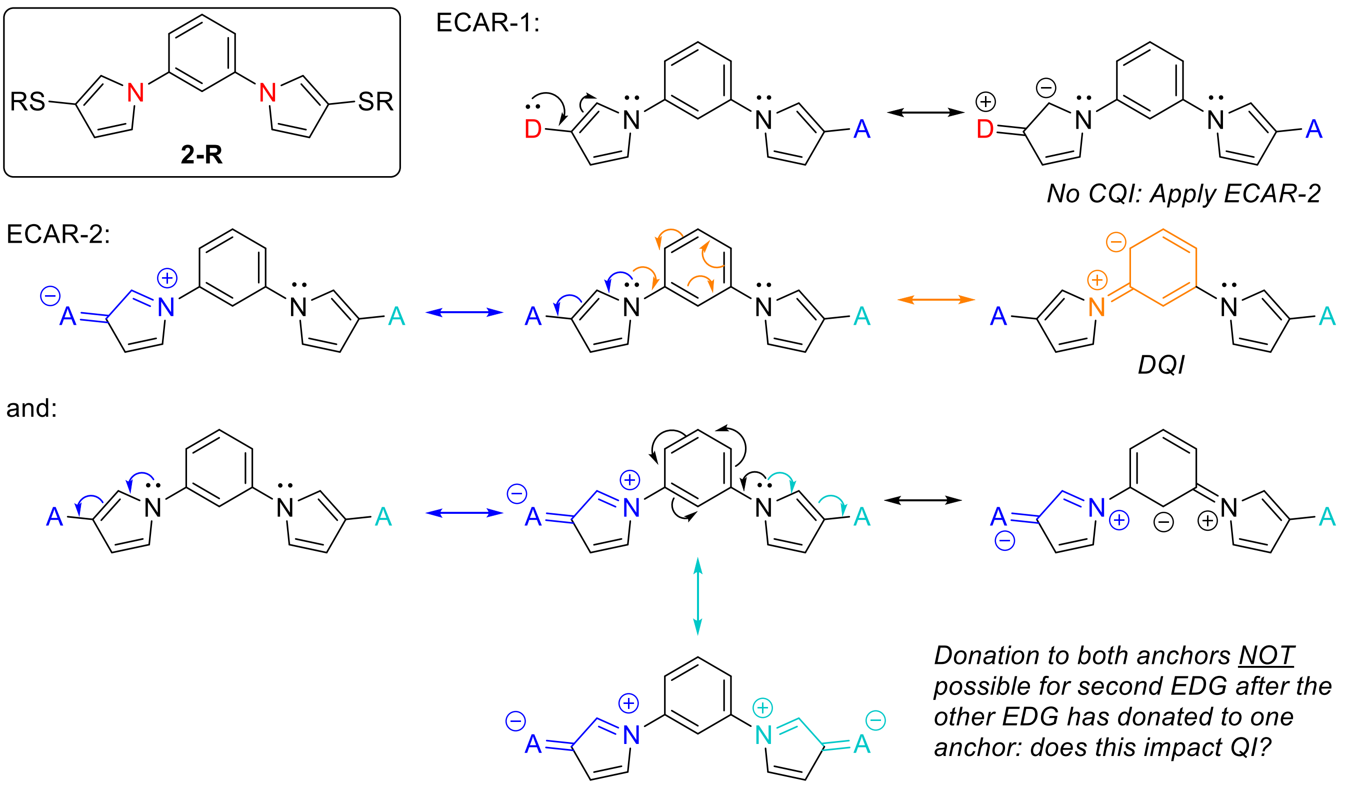

Figure S2. Analysis of the **2-SR** general molecular wire structure using ECARs, [1] and investigation of curly arrow interactions beyond ECARs for this system.

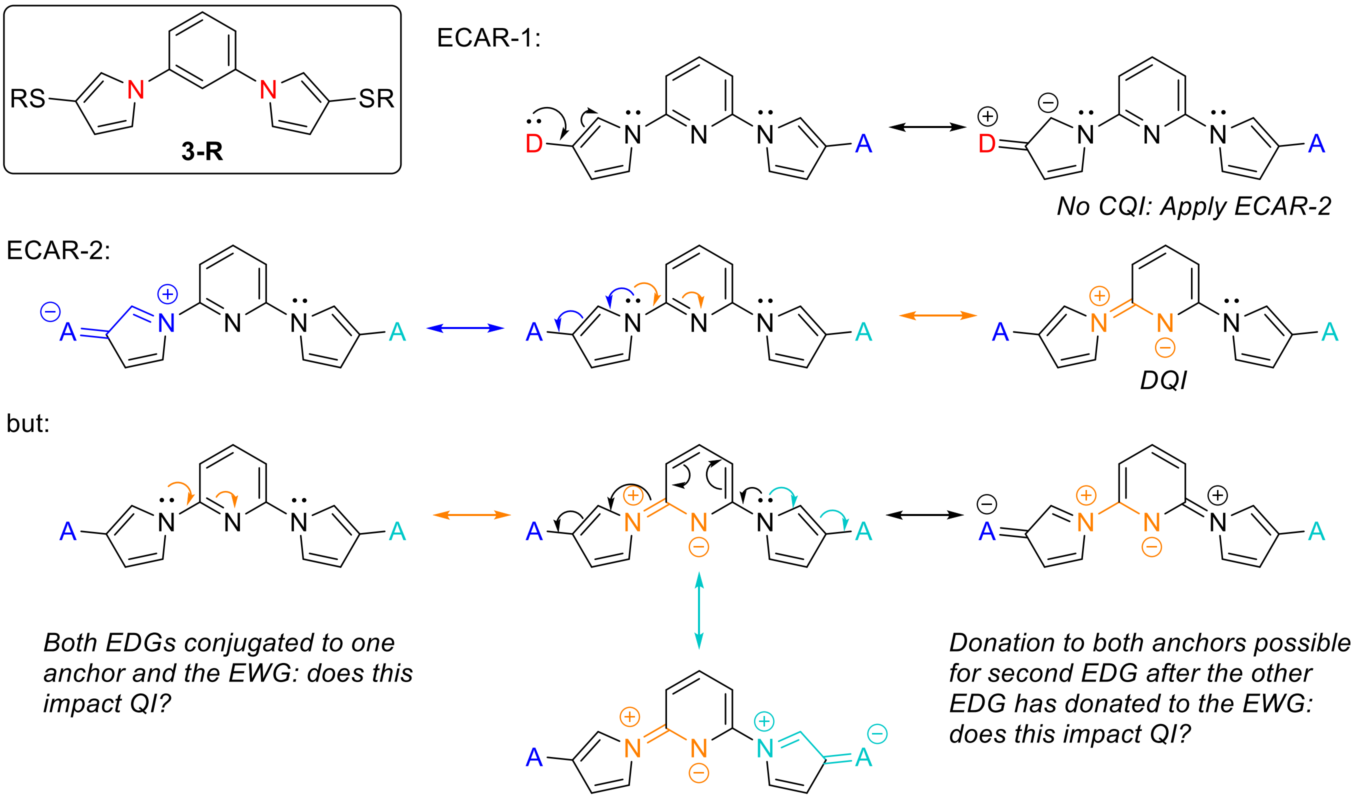

Figure S3. Analysis of the **3-SR** general molecular wire structure using ECARs, [1] and investigation of curly arrow interactions beyond ECARs for this system.

1.2: Literature Conductance Comparisons

Experimental molecular conductance values from a range of literature sources are compared with the data collected in this work in Table 2 of the main manuscript. Table S1 shows the structures of the molecular wires used in these comparisons.

Table S1: Structures of molecules used for conductance comparisons listed in manuscript Table 2

System	"*para*" species	"*meta*" species	"2,6-py" species
**1-SMe**, **2-SMe**, **3-SMe** (this work)	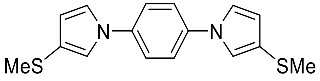	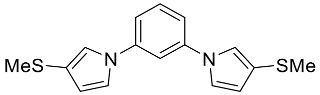	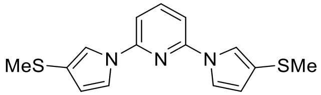
**1-S**, **2-S**, **3-S** (this work)	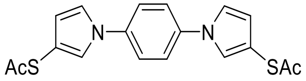	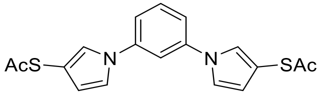	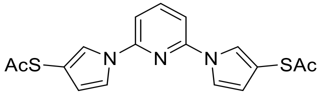
Thiolate-anchored OAEs[3]	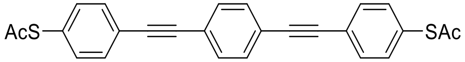	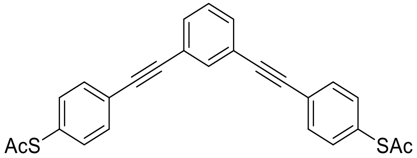	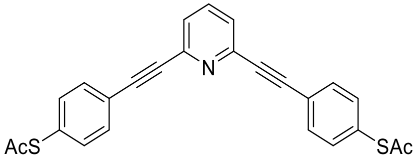
Thiomethyl-anchored terphenyls and derivatives[4, 5]	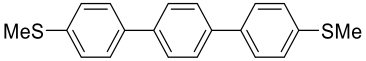	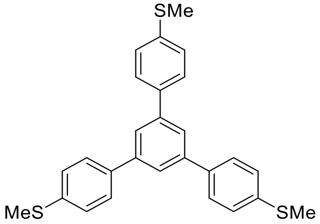	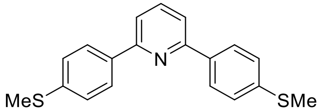
Thiolate-anchored OAEs[6]	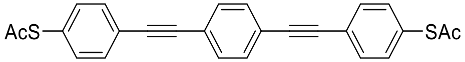	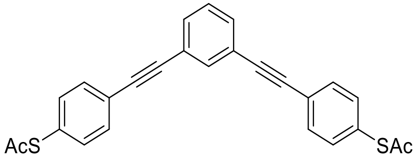	-

System	"*para*" species	"*meta*" species	"2,6-py" species
para-Pyridyl-anchored OAEs (MCBJ) [7]	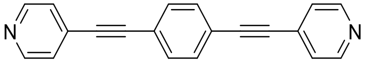	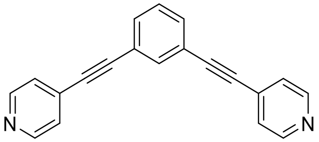	-
para-Pyridyl-anchored OAEs (STM-BJ) [7]	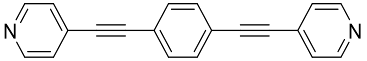	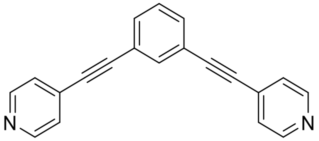	-
meta-Pyridyl-anchored OAEs[7]	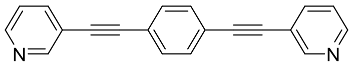	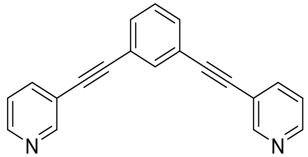	-
DHBT-anchored OAEs[8]	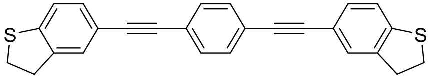	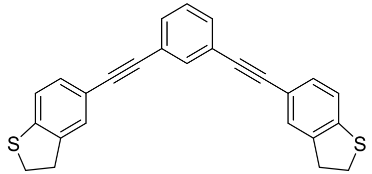	-
Iodo-anchored terphenyls[9]	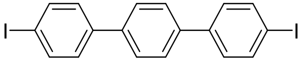	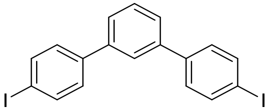	-
5-oxazolyl-anchored triaryls[10]	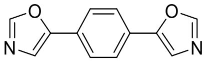	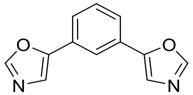	-
4-oxazolyl-anchored triaryls[10]	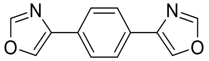	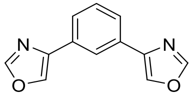	-
Unsymmetrical 4- and 5-oxazolyl-anchored triaryls[10]	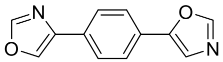	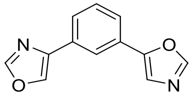	-

2. SYNTHESIS AND CHARACTERIZATION

2.1: General experimental methods

Synthetic reagents were purchased commercially and used as received unless otherwise stated. 3-methylthio-1-(triisopropylsilyl)pyrrole was prepared as reported previously. [11] Anhydrous solvents were prepared using an Innovative Technology solvent purification system and stored in ampoules under argon, except for anhydrous 1,4-dioxane (Acros Organics Extra Dry Acroseal®, used as received). Thin-layer chromatography (TLC) analysis was carried out using Merck silica gel 60 F_{254} TLC plates and spots were visualized using a UV lamp emitting at 365 or 254 nm. Column chromatography was performed using silica gel 60A (40-63 µm) purchased from Fluorochem. ^{1}H and ^{13}C NMR spectroscopy was carried out on a Bruker Avance III-HD-400 NMR spectrometer, Bruker Neo-400 NMR spectrometer or Varian VNMRS-600 NMR spectrometer. For ^{1}H NMR spectra, chemical shifts are reported relative to the residual solvent peak (7.26 ppm for $CHCl_3$; 2.05 ppm for acetone) and for ^{13}C NMR spectra, chemical shifts are reported relative to the solvent peak (77.16 ppm for $CDCl_3$; 29.84 ppm for acetone). These, and any other residual solvent peaks were referenced to values reported in the literature. [12] All NMR spectra were processed using MestReNova V12. ESI mass spectrometry was carried out using a Waters Acquity SQD spectrometer (Waters Ltd, UK) in flow injection analysis mode with acetonitrile as the mobile phase, subsequent accurate mass measurements used a QtoF Premier mass spectrometer (Waters Ltd, UK). ASAP mass spectrometry (including accurate mass measurements) was carried out using an LCT Premier XE mass spectrometer (Waters Ltd, UK) using TOF detection. Exact mass measurements were processed using Elemental Composition 4.0 embedded within MassLynx 4.1 (Waters Ltd, UK). Elemental analysis was performed on an Exeter Analytical E-440 machine.

Unless otherwise stated, reactions were conducted under an argon atmosphere. Where anhydrous solvents were used, glassware was first dried under vacuum using a heat gun, then filled directly with argon. Solvents and liquid reagents were added by syringe or cannula, and solid reagents were added under a positive pressure of argon. Degassing was conducted by bubbling argon through the reaction mixture using an argon-filled balloon fitted with a syringe needle. All mixed solvents in this work were prepared as v/v mixtures.

2.2: Synthetic procedures

2.2.1: Thiomethyl-anchored species

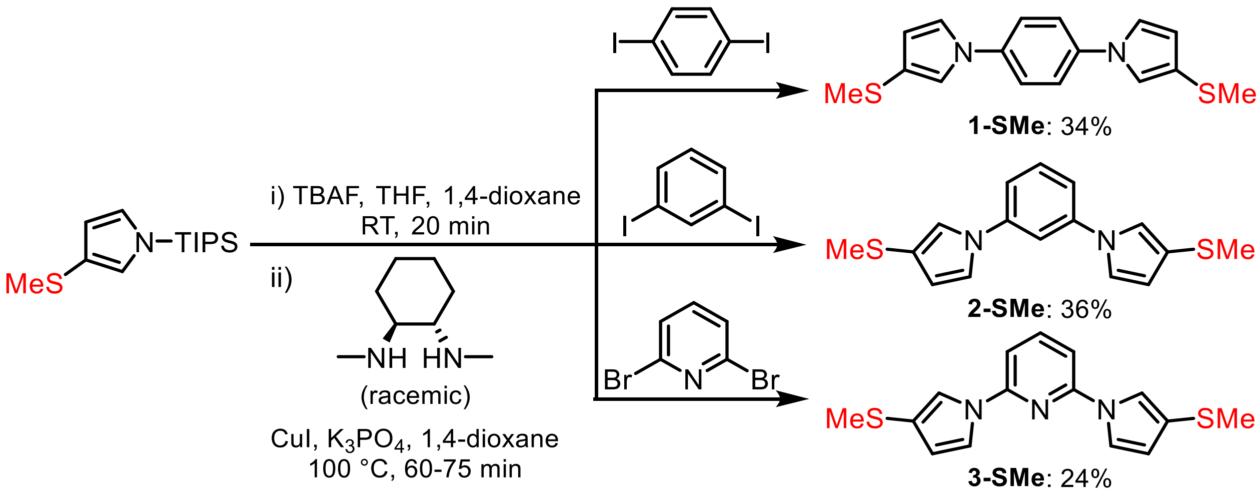

Scheme S1. Synthesis of thiomethyl-anchored triaryl molecular wires **1-SMe**, **2-SMe** and **3-SMe**.

1,4-bis(3-methylthiopyrrol-1-yl)benzene (1-SMe)

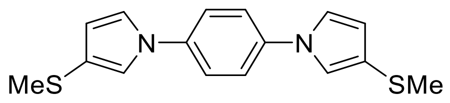

Based on previously reported conditions for C-N coupling of pyrrole derivatives and aryl halides. [11, 13, 14]

3-methylthio-1-(triisopropylsilyl)pyrrole[11] (502 mg, 1.86 mmol, 2.05 eq.) and 1,4-diiodobenzene (300 mg, 0.91 mmol, 1 eq.) were dissolved in anhydrous 1,4-dioxane (30 mL) in a dry flask under Ar at RT and the solution was degassed (Ar, 10 min). While degassing continued, TBAF (1.0 M in THF, 1.91 mL, 1.91 mmol, 2.1 eq.) was added and the solution was stirred while degassing (Ar, 15 min). CuI (355 mg, 1.86 mmol, 2.05 eq.), K_3PO_4 (1.19 g, 5.59 mmol, 6.15 eq.) and (±)-*trans-N,N'*-dimethylcyclohexane-1,2-diamine (0.59 mL, 3.7 mmol, 4.1 eq.) were added and the reaction was heated to 100 °C for 1 h after which TLC indicated the reaction was complete. After cooling to RT the mixture was filtered through a pad of celite, washing with CH_2Cl_2 (ca. 200 mL) to ensure all soluble materials were collected. The filtrate was washed with 2 M $NaOH_{(aq)}$ (3 × 50 mL) and deionized H_2O (3 × 50 mL) then the organic phase was dried ($MgSO_4$) before the solvent was removed *in vacuo*, affording a brown solid (1.15 g). The crude product was redissolved in CH_2Cl_2 and celite (30 mL) was added before evaporating to dryness. The crude material on celite was then purified by column chromatography (3 cm Ø, 100 mL SiO_2, gradient elution from hexane to 9:1 hexane/EtOAc) which afforded **1-SMe** as a fluffy white solid (93 mg, 34%).

^{1}H NMR (400 MHz, $CDCl_3$) δ 7.41 (s, 4H), 7.08 (t, *J* = 2.3, 1.7 Hz, 2H), 7.04 (dd, *J* = 2.9, 2.3 Hz, 2H), 6.39 (dd, *J* = 2.9, 1.7 Hz, 2H), 2.42 (s, 6H).

^{13}C NMR (101 MHz, $CDCl_3$) δ 138.2, 121.5, 120.5, 120.3, 118.3, 113.9, 20.3.

MS (ASAP-TOF): m/z: 301.1 $[M+H]^+$, 300.1 $[M]^{+\cdot}$, 254.1 $[M+H\text{-}SMe]^{+\cdot}$

HRMS (ASAP-TOF): m/z $[M+H]^+$ calculated for $C_{16}H_{17}N_2S_2$, 301.0833; found, 301.0824.

elem. anal.: Anal. Calcd for $C_{16}H_{16}N_2S_2$: C, 63.97; H, 5.37; N, 9.32. Found: C, 63.88; H, 5.37; N, 9.26.

1,3-bis(3-methylthiopyrrol-1-yl)benzene (2-SMe)

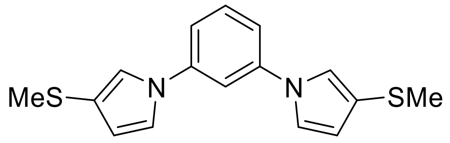

Based on previously reported conditions for C-N coupling of pyrrole derivatives and aryl halides[11, 13, 14]

3-methylthio-1-(triisopropylsilyl)pyrrole[11] (502 mg, 1.86 mmol, 2.05 eq.) and 1,3-diiodobenzene (300 mg, 0.91 mmol, 1 eq.) were dissolved in anhydrous 1,4-dioxane (30 mL) in a dry flask under Ar at RT and the solution was degassed (Ar, 10 min). While degassing continued, TBAF (1.0 M in THF, 1.91 mL, 1.91 mmol, 2.1 eq.) was added and the solution was stirred while degassing (Ar, 15 min). CuI (355 mg, 1.86 mmol, 2.05 eq.), K_3PO_4 (1.19 g, 5.59 mmol, 6.15 eq.) and (±)-*trans-N,N'*-dimethylcyclohexane-1,2-

diamine (0.59 mL, 3.7 mmol, 4.1 eq.) were added and the reaction was heated to 100 °C for 1 h after which TLC indicated the reaction was complete. After cooling to RT the mixture was filtered through a pad of celite, washing with CH_2Cl_2 (ca. 200 mL) to ensure all soluble materials were collected. The filtrate was washed with 2 M $NaOH_{(aq)}$ (3 × 50 mL) and deionized H_2O (3 × 50 mL) then the organic phase was dried ($MgSO_4$) before the solvent was removed *in vacuo*, affording a light brown solid (1.25 g). The crude product was redissolved in CH_2Cl_2 and celite (30 mL) was added before evaporating to dryness. The crude material on celite was then purified by repeated column chromatography (1: 3 cm Ø, 100 mL SiO_2, gradient elution from hexane to 94:6 hexane/EtOAc; 2: 2 cm Ø, 50 mL SiO_2, gradient elution from hexane to 97:3 hexane/EtOAc). The near-pure product (143 mg) was then dissolved in CH_2Cl_2 and stirred for 30 min with a small quantity of active carbon which was then removed by filtration. The filtrate was washed with 2 M NaOH (3 × 20 mL) then the organic layer was dried ($MgSO_4$) before the solvent was removed *in vacuo*, affording a yellow oil (109 mg). As small impurity signals were still visible in the ^{1}H NMR spectrum, the material was further purified by column chromatography (2 cm Ø, 70 mL SiO_2, gradient elution from hexane to 3:1 hexane/CH_2Cl_2) which afforded **2-SMe** as a yellow oil (97 mg, 36%).

^{1}H NMR (400 MHz, $CD_3C(O)CD_3$) δ 7.73 (t, *J* = 2.2 Hz, 1H), 7.55 (dd, *J* = 8.8, 7.3 Hz, 1H), 7.46 – 7.40 (m, 6H), 6.35 (dd, *J* = 3.0, 1.8 Hz, 2H), 2.37 (s, 6H).

^{13}C NMR (101 MHz, $CD_3C(O)CD_3$) δ 142.1, 131.9, 121.0, 120.7, 119.4, 117.1, 114.2, 111.5, 19.6.

MS (ASAP-TOF): m/z: 301.1 $[M+H]^+$, 300.1 $[M]^{+\cdot}$, 254.1 $[M+H\text{-}SMe]^{+\cdot}$

HRMS (ASAP-TOF): m/z $[M+H]^+$ calculated for $C_{16}H_{17}N_2S_2$, 301.0833; found, 301.0826.

2,6-bis(3-methylthiopyrrol-1-yl)pyridine (3-SMe)

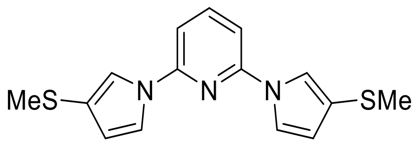

Based on previously reported conditions for C-N coupling of pyrrole derivatives and aryl halides[11, 13, 14]

3-methylthio-1-(triisopropylsilyl)pyrrole[11] (504 mg, 1.87 mmol, 2.05 eq.) and 2,6-dibromopyridine (216 mg, 0.91 mmol, 1 eq.) were dissolved in anhydrous 1,4-dioxane (30 mL) in a dry flask under Ar at RT and the solution was degassed (Ar, 10 min). While degassing continued, TBAF (1.0 M in THF, 1.91 mL, 1.91 mmol, 2.1 eq.) was added and the solution was stirred while degassing (Ar, 15 min). CuI (356 mg, 1.87 mmol, 2.05 eq.), K_3PO_4 (1.19 g, 5.59 mmol, 6.15 eq.) and (±)-*trans-N,N′*-dimethylcyclohexane-1,2-diamine (0.59 mL, 3.7 mmol, 4.1 eq.) were added and the reaction was heated to 100 °C for 75 min after which TLC indicated the reaction was complete. After cooling to RT the mixture was filtered through a pad of celite, washing with CH_2Cl_2 (ca. 200 mL) to ensure all soluble materials were collected. The filtrate was washed with 2 M $NaOH_{(aq)}$ (3 × 50 mL) and deionized H_2O (3 × 50 mL) then the organic phase was dried ($MgSO_4$) before the solvent was removed *in vacuo*, affording a purple-brown solid (1.05 g). The crude product was redissolved in CH_2Cl_2 and celite (30 mL) was added before evaporating to dryness. The crude material on celite was then purified by repeated column chromatography (1: 3 cm Ø, 100 mL SiO_2, 5:1 hexane/EtOAc; 2: 2 cm Ø, 50 mL SiO_2, 3:1 hexane/CH_2Cl_2; 3: 2 cm Ø, 50 mL SiO_2,

gradient elution from 17:3 hexane/CH_2Cl_2 to 3:1 hexane/CH_2Cl_2) which afforded **3-SMe** as a beige solid (67 mg, 24%).

^{1}H NMR (400 MHz, $CD_3C(O)CD_3$) δ 7.98 (t, *J* = 8.1 Hz, 1H), 7.76 (dd, *J* = 3.2, 2.3 Hz, 2H), 7.70 (dd, *J* = 2.3, 1.7 Hz, 2H), 7.43 (d, *J* = 8.1 Hz, 2H), 6.36 (dd, *J* = 3.2, 1.7 Hz, 2H), 2.40 (s, 6H).

^{13}C NMR (101 MHz, $CD_3C(O)CD_3$) δ 150.5, 142.8, 120.6, 120.1, 118.9, 114.5, 107.7, 18.9.

MS (ASAP-TOF): m/z: 302.1 $[M+H]^+$, 301.1 $[M]^{+\cdot}$, 255.1 $[M+H\text{-}SMe]^{+\cdot}$

HRMS (ASAP-TOF): m/z $[M+H]^+$ calculated for $C_{15}H_{16}N_3S_2$, 302.0786; found, 302.0779.

elem. anal.: Anal. Calcd for $C_{15}H_{15}N_3S_2$ + 0.15 CH_2Cl_2: C, 57.92; H, 4.91; N, 13.38 Found: C, 57.81; H, 4.89; N, 13.39.

2.2.2: Protected thiol-anchored species

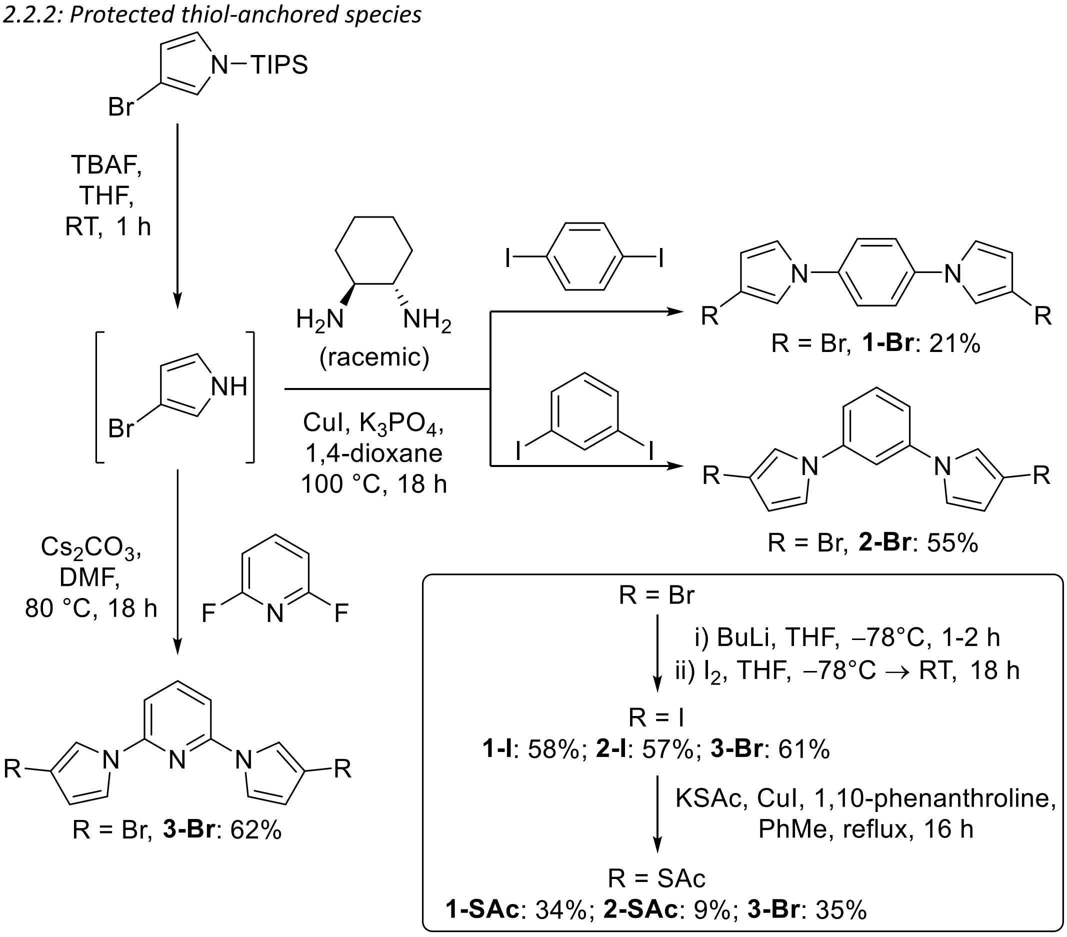

Scheme S2. Synthesis of protected thiol-anchored triaryl molecular wires **1-SAc**, **2-SAc** and **3-SAc**.

3-bromopyrrole

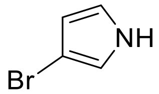

This product was used directly after work-up without further purification owing to concerns over the long-term stability of the *N*-unsubstituted pyrrole. [15, 16] Example procedure:

3-bromo-1(triisopropyl)pyrrole (0.69 mL, 2.7 mmol, 1 eq.), was dissolved in anyhydrous THF (8 mL) in a dry flask under Ar at RT. To this solution was added dropwise TBAF (1.0 M in THF, 2.8 mL, 2.8 mmol, 2.5 eq.) and the mixture was stirred at RT for 1 hour. The reaction was quenched with distilled water (10 mL), then the product was extracted with diethyl ether (3 x 10 mL). The organic phase was dried ($MgSO_4$) before the solvent was removed *in vacuo* affording 3-bromopyrrole of sufficient purity to continue the synthesis directly as a pale yellow oil.

A small sample was characterized as outlined below.

^{1}H NMR (400 MHz, $CDCl_3$) 8.98 (bs, 1H), 6.78 – 6.72 (m, 1H), 6.71 – 6.64 (m, 1H), 6.22 – 6.16 (m, 1H).

^{13}C NMR (101 MHz, $CDCl_3$) δ 118.8, 117.8, 110.9, 96.0.

^{1}H NMR data agrees with previous reports. [15, 16]

1,4-bis(3-bromopyrrol-1-yl)benzene (1-Br)

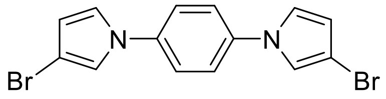

1,4-diiodobenzene (0.5 g, 1.5 mmol, 1 eq.), freshly prepared 3-bromo-1*H*-pyrrole (prepared as described above from 2.7 mmol 3-bromo-1(triisopropyl) pyrrole, ≤ 2.7 mmol, ≤ 1.8 eq.), copper(I) iodide (0.5 g, 2.6 mmol, 1.75 eq.) and K_3PO_4 (1.7 g, 8.0 mmol, 5.3 eq.) were added to anhydrous 1,4-dioxane (20 mL) in a dry flask under Ar at RT and the solution was degassed (Ar, 10 min). (±)-*trans*-1,2-Diaminocyclohexane (0.63 mL, 5.3 mmol, 3.5 eq.) was added and the reaction was stirred at reflux overnight, until TLC analysis (5% EtOAc in hexanes) indicated complete consumption of the starting materials. The reaction mixture was filtered through a pad of celite, washing with CH_2Cl_2 (150 mL). The volume of the filtrate was reduced to *ca.* 100 mL before washing with $NaOH_{(aq)}$ (1M, 3 x 50 mL), followed by H_2O (3 x 50 mL). The organic phase was dried ($MgSO_4$) before the solvent was removed *in vacuo* to give the crude product as a dark brown oil. Purification by column chromatography (SiO_2, gradient elution from hexane to 9:1 hexane/EtOAc) gave **1-Br** as a white solid (114 mg, 21%[*]).

^{1}H NMR (400 MHz, $CDCl_3$) δ 7.40 (s, 4H), 7.07 (t, *J* = 2.4, 1.7 Hz, 2H), 6.97 (t, *J* = 3.0, 2.4 Hz, 2H), 6.36 (dd, *J* = 3.0, 1.7 Hz, 2H).

^{13}C NMR (101 MHz, $CD_3C(O)CD_3$) δ 138.6, 121.9, 121.0, 119.9, 113.8, 98.9.

MS (ESI-SQD): m/z: m/z: 365.0 $[M+H]^+$.

[*] From 1,4-diiodobenzene.

HRMS (ESI-TOF): m/z $[M+H]^+$ calculated for $C_{14}H_{11}N_2{}^{79}Br_2$, 364.9289; found, 364.9304.

1,3-bis(3-bromopyrrol-1-yl)benzene (2-Br)

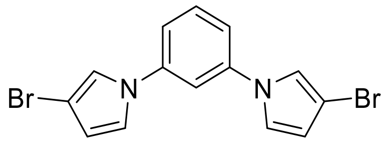

1,3-diiodobenzene (2.1 g, 6.3 mmol, 1 eq.), freshly prepared 3-bromo-1*H*-pyrrole (prepared as described above from 13.2 mmol 3-bromo-1(triisopropyl) pyrrole, ≤ 13.2 mmol, ≤ 2.1 eq.), copper(I) iodide (2.4 g, 12.6 mmol, 2 eq.) and K_3PO_4 (6.7 g, 31.5 mmol, 5 eq.) were added to anhydrous 1,4-dioxane (50 mL) in a dry flask under Ar at RT and the solution was degassed (Ar, 20 min). (±)-*trans*-1,2-Diaminocyclohexane (1.5 mL, 12.5 mmol, 2 eq.) was added and the reaction was stirred at reflux overnight, until TLC analysis (5% EtOAc in hexanes) indicated complete consumption of the starting materials. The reaction mixture was filtered through a pad of celite, washing with CH_2Cl_2 (150 mL). The volume of the filtrate was reduced to *ca.* 100 mL before washing with $NaOH_{(aq)}$ (1M, 3 x 50 mL), followed by H_2O (3 x 50 mL). The organic phase was dried ($MgSO_4$) before the solvent was removed *in vacuo* to give the crude product as a dark brown oil. Purification by column chromatography (SiO_2, gradient elution from hexane to 4:1 hexane/CH_2Cl_2) gave **2-Br** of sufficient purity to continue the synthesis* as a pale yellow oil (1.28 g, 55%†).

^{1}H NMR (400 MHz, $CDCl_3$) δ 7.48 (t, *J* = 8.1 Hz, 1H), 7.30 (t, *J* = 2.1 Hz, 1H), 7.25 (dd, *J* = 8.1, 2.1 Hz, 2H), 7.10 (dd, *J* = 2.5, 1.7 Hz, 2H), 7.00 (dd, *J* = 3.1, 2.5 Hz, 2H), 6.37 (dd, *J* = 3.1, 1.7 Hz, 2H).

MS (ESI-SQD): m/z: 365.1 $[M+H]^+$.

HRMS (ESI-TOF): m/z $[M+H]^+$ calculated for $C_{14}H_{11}N_2{}^{79}Br_2$, 364.9289; found, 364.9301.

2,6-bis(3-bromopyrrol-1-yl)pyridine (3-Br)

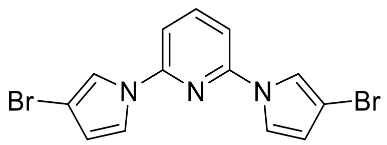

Freshly prepared 3-bromo-1*H*-pyrrole (prepared as described above from 2.7 mmol 3-bromo-1(triisopropyl) pyrrole, ≤ 2.7 mmol, ≤ 2.1 eq.) and anhydrous caesium carbonate (940 mg, 2.9 mmol, 2.2 eq.) were dissolved in anhydrous DMF (5 mL) in a dry flask under Ar at RT, and the resulting mixture was stirred for 10 minutes at RT. 2,6-Difluoropyridine (0.12 mL, 1.3 mmol, 1 eq.) was added and the mixture was stirred at 80 °C overnight. The reaction mixture was then diluted with CH_2Cl_2 (20 mL) and the suspension was poured into 50 mL brine. The organic phase was separated and the aqueous phase further extracted with CH_2Cl_2 (3 x 40 mL). The combined organic phases were dried ($MgSO_4$) before the solvent was removed *in vacuo* to give the crude product as a yellow oil. Purification by column

* Peaks indicative of trace quantities of another pyrrole derivative were visible in the ^{1}H NMR spectrum.

† From 1,3-diiodobenzene.

chromatography (SiO_2, gradient elution from 19:1 hexane/CH_2Cl_2 to 4:1 hexane/CH_2Cl_2) gave the **3-Br** as a white solid (302 mg, 62%).

^{1}H NMR (400 MHz, $CD_3C(O)CD_3$) δ 8.04 (t, *J* = 8.1 Hz, 1H), 7.86 (apparent t*, *J* = 2.1 Hz, 2H), 7.76 (apparent t†, *J* = 2.9 Hz, 2H), 7.51 (d, *J* = 8.1 Hz, 2H), 6.39 (dd, *J* = 3.3, 1.7 Hz, 2H).

^{13}C NMR (101 MHz, $CD_3C(O)CD_3$) δ 150.1, 143.1, 120.1, 119.0, 114.7, 108.3, 100.3.

MS (ESI-SQD): m/z: 366.1 $[M+H]^+$.

HRMS (ESI-TOF): m/z $[M+H]^+$ calculated for $C_{13}H_{10}N_3{}^{79}Br_2$, 365.9241; found, 365.9237.

1,4-bis(3-iodomopyrrol-1-yl)benzene (1-I)

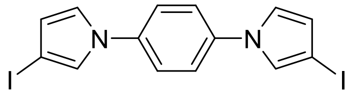

1,4-bis(3-bromo-1*H*-pyrrol-1-yl)benzene, **1-Br**, (200 mg, 0.55 mmol, 1 eq.) was dissolved in anhydrous THF (10 mL, dried over 4 Å molecular sieves) in a dry flask under Ar at RT before the solution was cooled to −78 °C. *n*-Butyllithium (2.5 M in hexanes, 0.72 mL, 1.8 mmol, 3.3 eq.) was added dropwise and the reaction was stirred at −78 °C for 2 hours. A previously prepared solution of iodine in anhydrous THF (0.11 M, 12.9 mL, 1.4 mmol, 2.5 eq., dried overnight over 4 Å molecular sieves after preparation) was added dropwise at −78 °C and the reaction mixture was allowed to warm to RT overnight. The reaction was quenched with saturated aqueous $Na_2S_2O_4$ solution (10 mL) and the mixture was extracted with CH_2Cl_2 (3 x 30 mL). The combined organic phases were dried ($MgSO_4$) before the solvent was removed *in vacuo* to give the crude product as an orange solid. Purification by column chromatography (SiO_2, gradient elution from hexane to 4:1 hexane/CH_2Cl_2) gave **1-I** as an off-white solid (147 mg, 58%).

^{1}H NMR (400 MHz, $CDCl_3$) δ 7.40 (s, 4H), 7.13 (dd, *J* = 2.3, 1.6 Hz, 2H), 6.95 (dd, *J* = 3.0, 2.3 Hz, 2H), 6.44 (dd, *J* = 3.0, 1.6 Hz, 2H).

^{13}C NMR (101 MHz, $CDCl_3$) δ 138.1, 124.3, 121.8, 121.3, 118.1, 62.6.

MS (ESI-SQD): m/z: 461.0 $[M+H]^+$.

HRMS (ESI-TOF): m/z $[M+H]^+$ calculated for $C_{14}H_{11}N_2I_2$, 460.9012; found, 460.9021.

* Believed to be a poorly resolved dd.
† Believed to be a poorly resolved dd.

1,3-bis(3-iodopyrrol-1-yl)benzene (2-I)

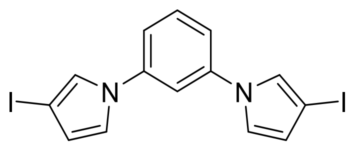

1,3-bis(3-bromo-1*H*-pyrrol-1-yl)benzene, **2-Br**, (1.25 g, 3.41 mmol, 1 eq.) was dissolved in anhydrous THF (20 mL, dried over 4 Å molecular sieves) in a dry flask under Ar at RT before the solution was cooled to −78 °C. *n*-Butyllithium (1.8 M in hexanes, 4.0 mL, 7.2 mmol, 2.1 eq.) was added dropwise and the reaction was stirred at −78 °C for 1 hour. A previously prepared solution of iodine in anhydrous THF (0.3 M, 32 mL, 9.6 mmol, 2.8 eq., dried overnight over 4 Å molecular sieves after preparation) was added dropwise at −78 °C and the reaction mixture was allowed to warm to RT overnight. The reaction was quenched with saturated aqueous $Na_2S_2O_4$ solution (20 mL) and the mixture was extracted with CH_2Cl_2 (3 x 50 mL). The combined organic phases were dried ($MgSO_4$) before the solvent was removed *in vacuo* to give the crude product. Purification by column chromatography (SiO_2, gradient elution from hexane to 4:1 hexane/CH_2Cl_2) gave **2-I** as a yellow oil of sufficient purity* for the following step (902 mg, 57%).

^{1}H NMR (400 MHz, $CDCl_3$) δ 7.48 (t, *J* = 8.1 Hz, 1H), 7.30 (t, *J* = 2.1 Hz, 1H), 7.24 (dd, *J* = 8.1, 2.1 Hz, 2H), 7.16 (dd, *J* = 2.3, 1.6 Hz, 2H), 6.98 (dd, *J* = 3.0, 2.3 Hz, 2H), 6.45 (dd, *J* = 3.0, 1.6 Hz, 2H).

MS (ESI-SQD): m/z: 461.0 $[M+H]^+$.

HRMS (ESI-TOF): m/z $[M+H]^+$ calculated for $C_{14}H_{11}N_2I_2$, 460.9012; found, 460.9024.

2,6-bis(3-iodopyrrol-1-yl)pyridine (3-I)

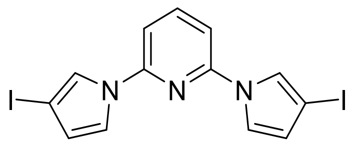

2,6-bis(3-bromo-1*H*-pyrrol-1-yl)pyridine, **3-Br**, (200 mg, 0.55 mmol, 1 eq.) was dissolved in anhydrous THF (10 mL, dried over 4 Å molecular sieves) in a dry flask under Ar at RT before the solution was cooled to −78 °C. *n*-Butyllithium (1.6 M in hexanes, 0.71 mL, 1.14 mmol, 2.1 eq.) was added dropwise and the reaction was stirred at −78 °C for 1 hour. A previously prepared solution of iodine in anhydrous THF (0.11 M, 12.9 mL, 0.79 mmol, 2.6 eq., dried overnight over 4 Å molecular sieves after preparation) was added dropwise at −78 °C and the reaction mixture was allowed to warm to RT overnight. The reaction was quenched with saturated aqueous $Na_2S_2O_4$ solution (10 mL) and the organic phase was separated and the aqueous phase further extracted with CH_2Cl_2 (3 x 30 mL). The combined organic phases were dried ($MgSO_4$) before the solvent was removed *in vacuo* to give the crude product as an

* Impurities present in the ^{1}H NMR spectrum were attributed to by-products in which one or both rings had been dehalogenated or remained brominated. As these were challenging to separate, they were carried forward into the next step as the larger polarity difference between the target dithioacetate product and these species was anticipated to facilitate purification. The conditions used for thioacetylation are only effective for aryl iodides, so residual bromides were not expected to disrupt the reaction.

orange solid. Purification by column chromatography (SiO_2, 19:1 hexane/EtOAc) gave **3-I** as a yellow solid of sufficient purity to continue the synthesis (170 mg, 61%).

^{1}H NMR (400 MHz, $CDCl_3$) δ 7.79 (t, *J* = 8.1 Hz, 1H), 7.63 – 7.57 (m, 2H), 7.43 – 7.36 (m, 2H), 7.06 (d, *J* = 8.1 Hz, 2H), 6.44 (dd, *J* = 3.2, 1.6 Hz, 2H).

MS (ESI-SQD): m/z: 462.0 $[M+H]^+$.

HRMS (ESI-TOF): m/z $[M+H]^+$ calculated for $C_{13}H_{10}N_3I_2$, 461.8964; found, 461.8969.

1,4-bis(3-acetylthiopyrrol-1-yl)benzene (1-SAc)

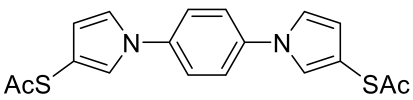

1,4-bis(3-iodo-1*H*-pyrrol-1-yl)benzene, **1-I**, (77 mg, 0.167 mmol, 1 eq.), potassium thioacetate (57 mg, 0.50 mmol, 3 eq.), copper(I) iodide (3 mg, 0.016 mmol, 0.1 eq.) and 1,10-phenanthroline (3 mg, 0.017 mmol, 0.1 eq.) were combined in anhydrous toluene (10 mL) in a dry flask under Ar at RT and the solution was degassed (Ar, 20 min). The mixture was stirred at reflux for 16 hours. The cooled mixture was diluted with CH_2Cl_2 (50 mL) then poured into water (80 mL). The organic phase was separated and the aqueous phase further extracted with CH_2Cl_2 (3 x 30 mL). The combined organic phases were dried ($MgSO_4$) before the solvent was removed *in vacuo* to give the crude product as a brown solid. Purification by column chromatography (SiO_2, gradient elution from hexane to 3:2 hexane/EtOAc) gave **1-SAc** as a yellow solid (20 mg, 34%).

^{1}H NMR (599 MHz, $CDCl_3$) δ 7.45 (s, 4H), 7.19 (dd, *J* = 2.3, 1.7 Hz, 2H), 7.13 (dd, *J* = 3.0, 2.3 Hz, 2H), 6.39 (dd, *J* = 3.0, 1.7 Hz, 2H), 2.40 (s, 6H).

^{13}C NMR (151 MHz, $CDCl_3$) δ 196.1, 138.4, 124.2, 121.9, 120.9, 115.9, 108.3, 29.8.

MS (ESI-SQD): m/z: 357.2 $[M+H]^+$.

HRMS (ESI-TOF): m/z $[M+H]^+$ calculated for $C_{18}H_{17}N_2O_2S_2$, 357.0731; found, 357.0736.

Due to the small quantity of purified material isolated, elemental analysis data was not collected for this species. NMR spectra are provided below.

1,3-bis(3-acetylthiopyrrol-1-yl)benzene (2-SAc)

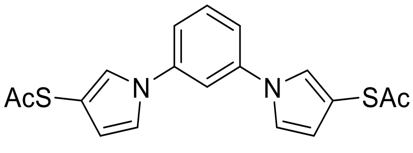

1,3-bis(3-iodo-1*H*-pyrrol-1-yl)benzene, **2-I**, (902 mg, 1.96 mmol, 1 eq.), potassium thioacetate (672 mg, 5.88 mmol, 3 eq.), copper(I) iodide (37 mg, 0.194 mmol, 0.1 eq.) and 1,10-phenanthroline (35 mg, 0.194 mmol, 0.1 eq.) were combined in anhydrous toluene (20 mL) in a dry flask under Ar at RT and

the solution was degassed (Ar, 20 min). The mixture was stirred at reflux for 16 hours. The cooled mixture was diluted with CH_2Cl_2 (50 mL) then poured into water (80 mL). The organic phase was separated and the aqueous phase further extracted with CH_2Cl_2 (3 x 30 mL). The combined organic phases were dried ($MgSO_4$) before the solvent was removed *in vacuo* to give the crude product as a brown solid. Purification by column chromatography (SiO_2, gradient elution from hexane to 4:1 hexane/EtOAc) gave **2-SAc** as a pink-brown solid. Additional material was isolated after crystallizing from impure fractions. Total yield (66 mg, 9%*).

^{1}H NMR (599 MHz, $CDCl_3$) δ 7.50 (t, *J* = 8.1 Hz, 1H), 7.39 (t, *J* = 2.2 Hz, 1H), 7.30 (dd, *J* = 8.1, 2.2 Hz, 2H), 7.21 (t, *J* = 2.3, 1.7 Hz, 2H), 7.16 (dd, *J* = 3.0, 2.3 Hz, 2H), 6.39 (dd, *J* = 3.0, 1.7 Hz, 2H), 2.40 (s, 6H).

^{13}C NMR (151 MHz, $CDCl_3$) δ 196.0, 141.3, 131.1, 124.2, 120.8, 118.5, 116.1, 113.1, 108.6, 31.1.

MS (ESI-SQD): m/z: 357.2 $[M+H]^+$.

HRMS (ESI-TOF): m/z $[M+H]^+$ calculated for $C_{18}H_{17}N_2O_2S_2$, 357.0731; found, 357.0747.

Due to the small quantity of purified material isolated, elemental analysis data was not collected for this species. NMR spectra are provided below.

2,6-bis(3-acetylthiopyrrol-1-yl)pyridine (3-SAc)

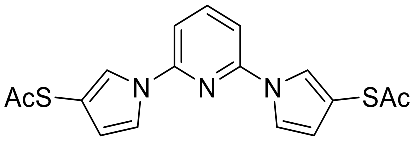

2,6-bis(3-iodo-1*H*-pyrrol-1-yl)pyridine, **3-I**, (100 mg, 0.217 mmol, 1 eq.), potassium thioacetate (76 mg, 0.665 mmol, 3 eq.), copper(I) iodide (10 mg, 0.053 mmol, 0.25 eq.) and 1,10-phenanthroline (12 mg, 0.067 mmol, 0.3 eq.) were combined in anhydrous toluene (5 mL) in a dry flask under Ar at RT and the solution was degassed (Ar, 20 min). The mixture was stirred at reflux for 16 hours. The cooled mixture was diluted with CH_2Cl_2 (50 mL) then poured into water (80 mL). The organic phase was separated and the aqueous phase further extracted with CH_2Cl_2 (3 x 30 mL). The combined organic phases were dried ($MgSO_4$) before the solvent was removed *in vacuo* to give the crude product as a brown solid. Purification by column chromatography (SiO_2, gradient elution from hexane to 3:2 hexane/EtOAc) gave **3-SAc** as a yellow solid (27 mg, 35%).

^{1}H NMR (400 MHz, $CDCl_3$) δ 7.83 (t, *J* = 8.0 Hz, 1H), 7.66 (dd, *J* = 2.3, 1.7 Hz, 2H), 7.58 (dd, *J* = 3.2, 2.3 Hz, 2H), 7.14 (d, *J* = 8.0 Hz, 2H), 6.39 (dd, *J* = 3.2, 1.7 Hz, 2H), 2.41 (s, 6H).

^{13}C NMR (101 MHz, $CDCl_3$) δ 195.8, 149.6, 141.5, 123.1, 119.4, 116.5, 109.5, 107.7, 29.9.

MS (ESI-SQD): m/z: 358.2 $[M+H]^+$.

HRMS (ESI-TOF): m/z $[M+H]^+$ calculated for $C_{17}H_{16}N_3O_2S_2$, 358.0684; found, 358.0697.

* The monosubstituted species was found to be the major product.

Due to the small quantity of purified material isolated, elemental analysis data was not collected for this compound. NMR spectra are provided below.

2.3: ^{1}H NMR spectra of compounds used in conductance studies

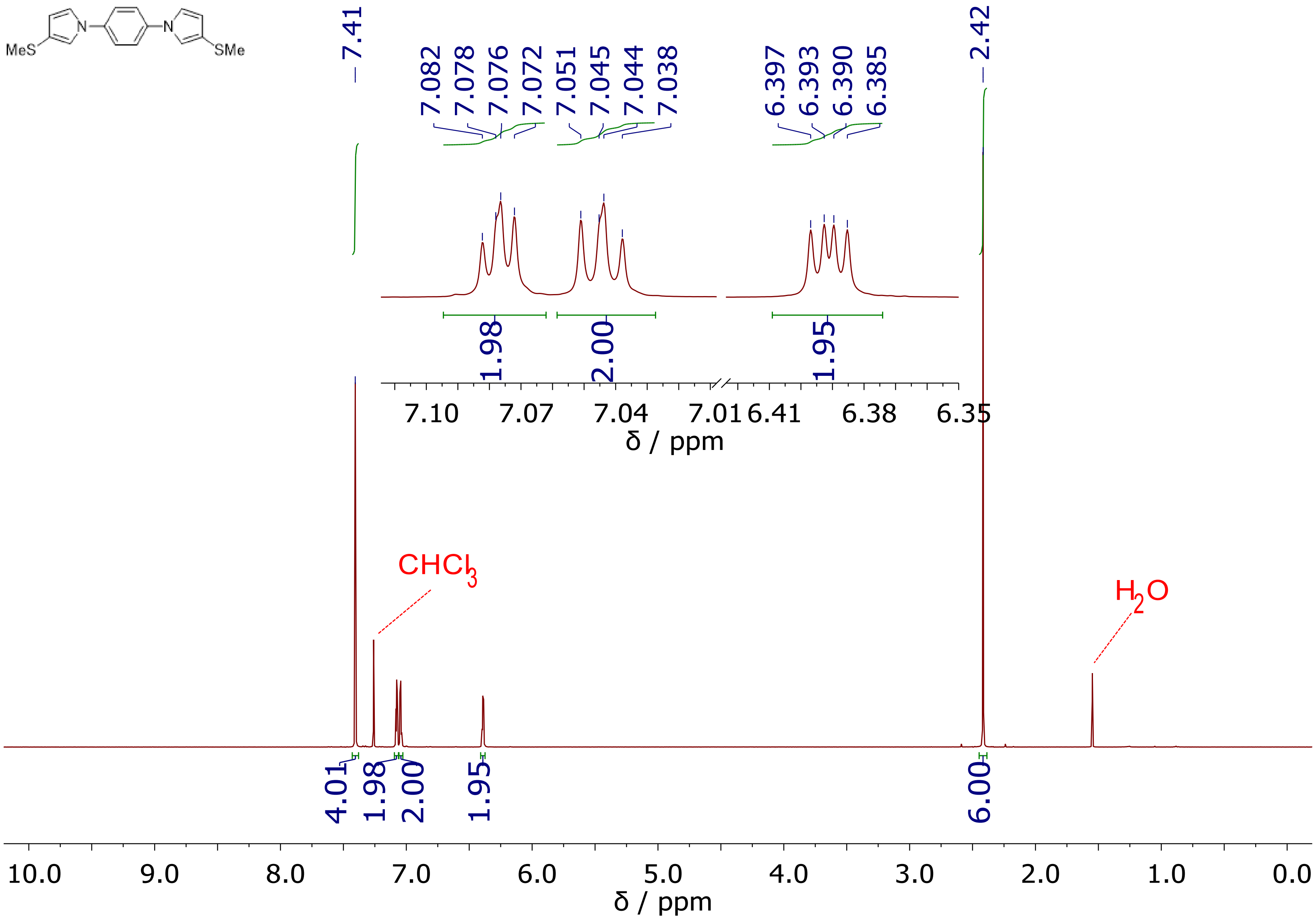

Figure S4. 400 MHz ^{1}H NMR spectrum of compound **1-SMe** in $CDCl_3$.

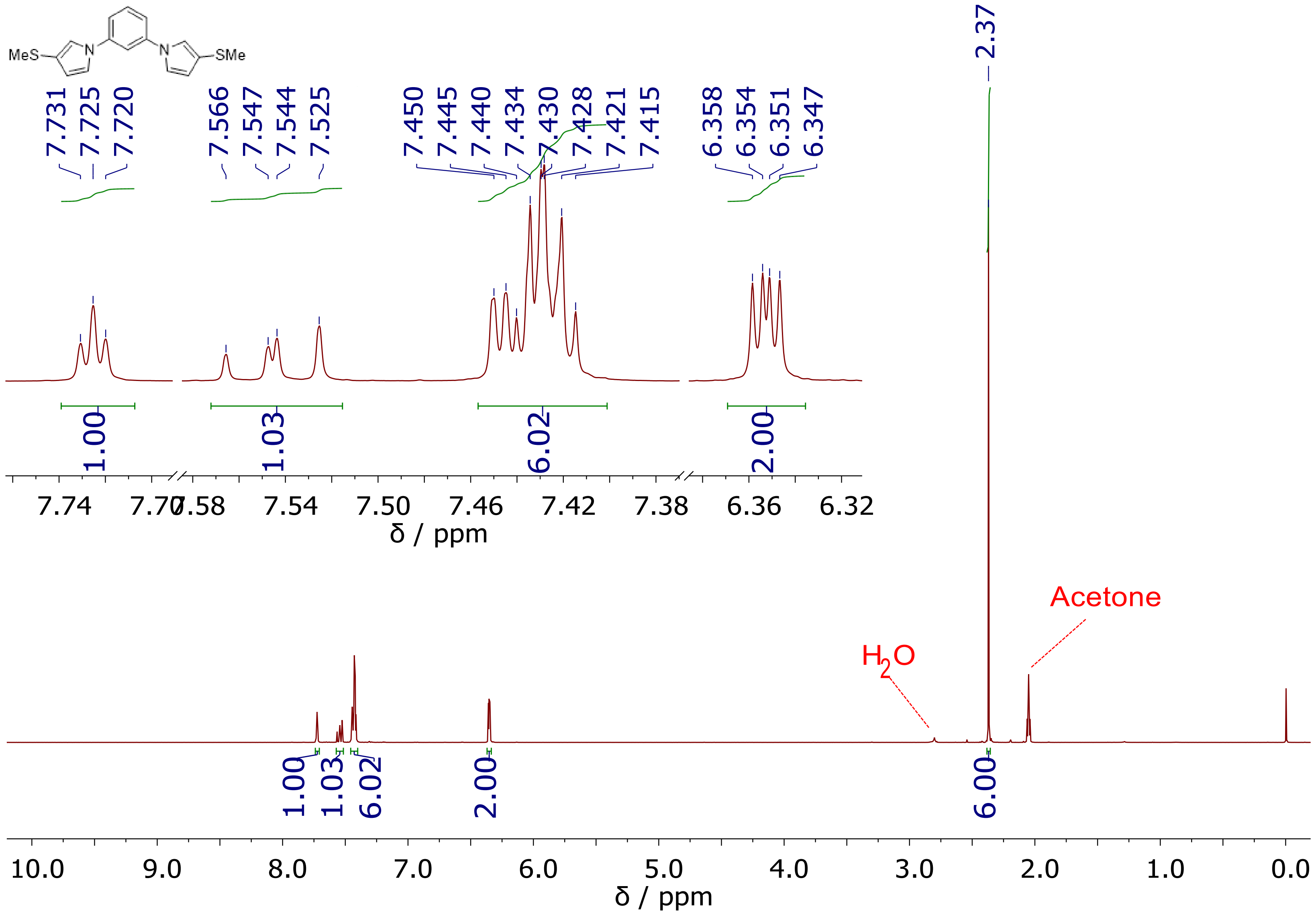

Figure S5. 400 MHz ^{1}H NMR spectrum of compound **2-SMe** in $CD_3C(O)CD_3$.

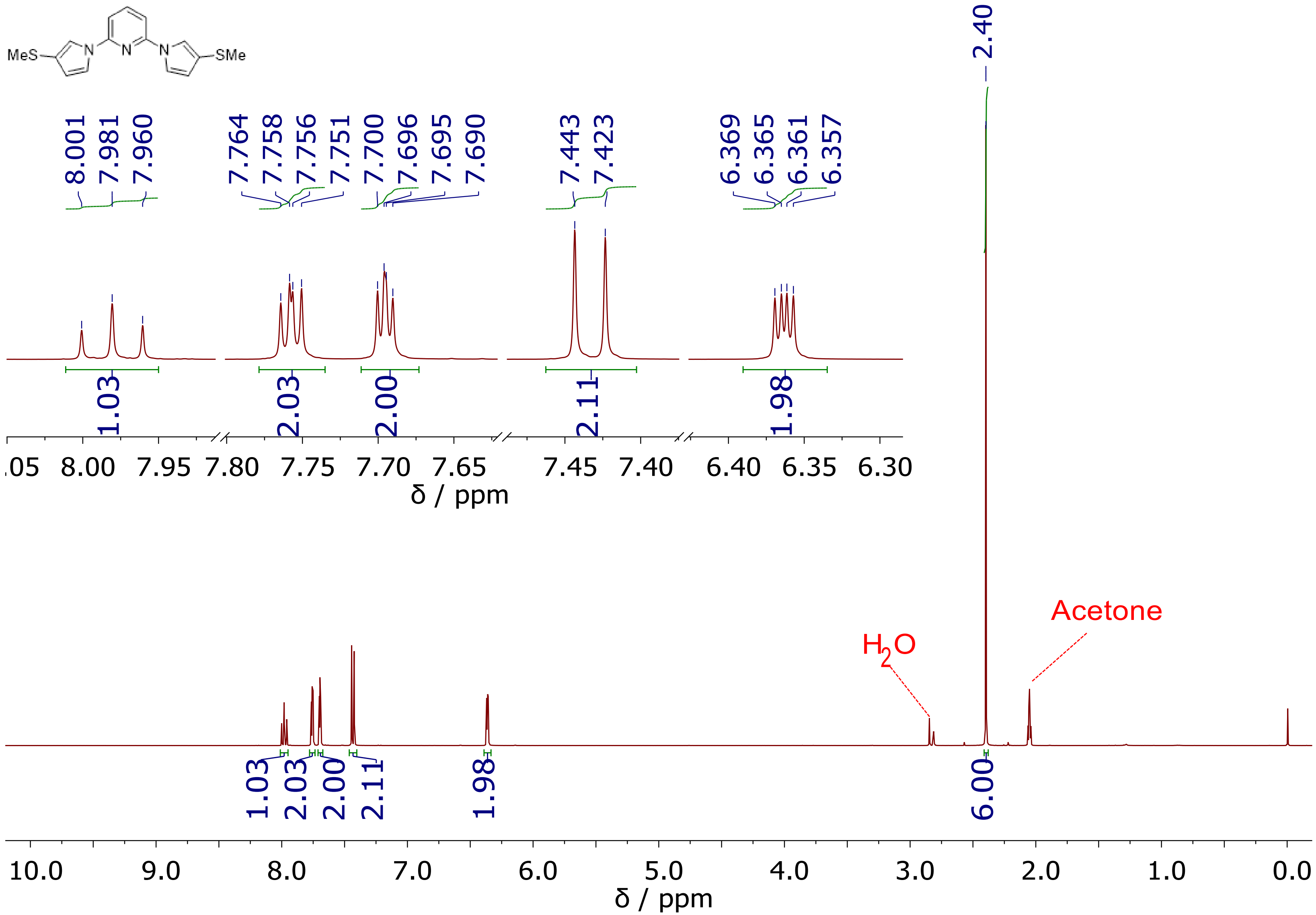

Figure S6. 400 MHz ^{1}H NMR spectrum of compound **3-SMe** in $CD_3C(O)CD_3$.

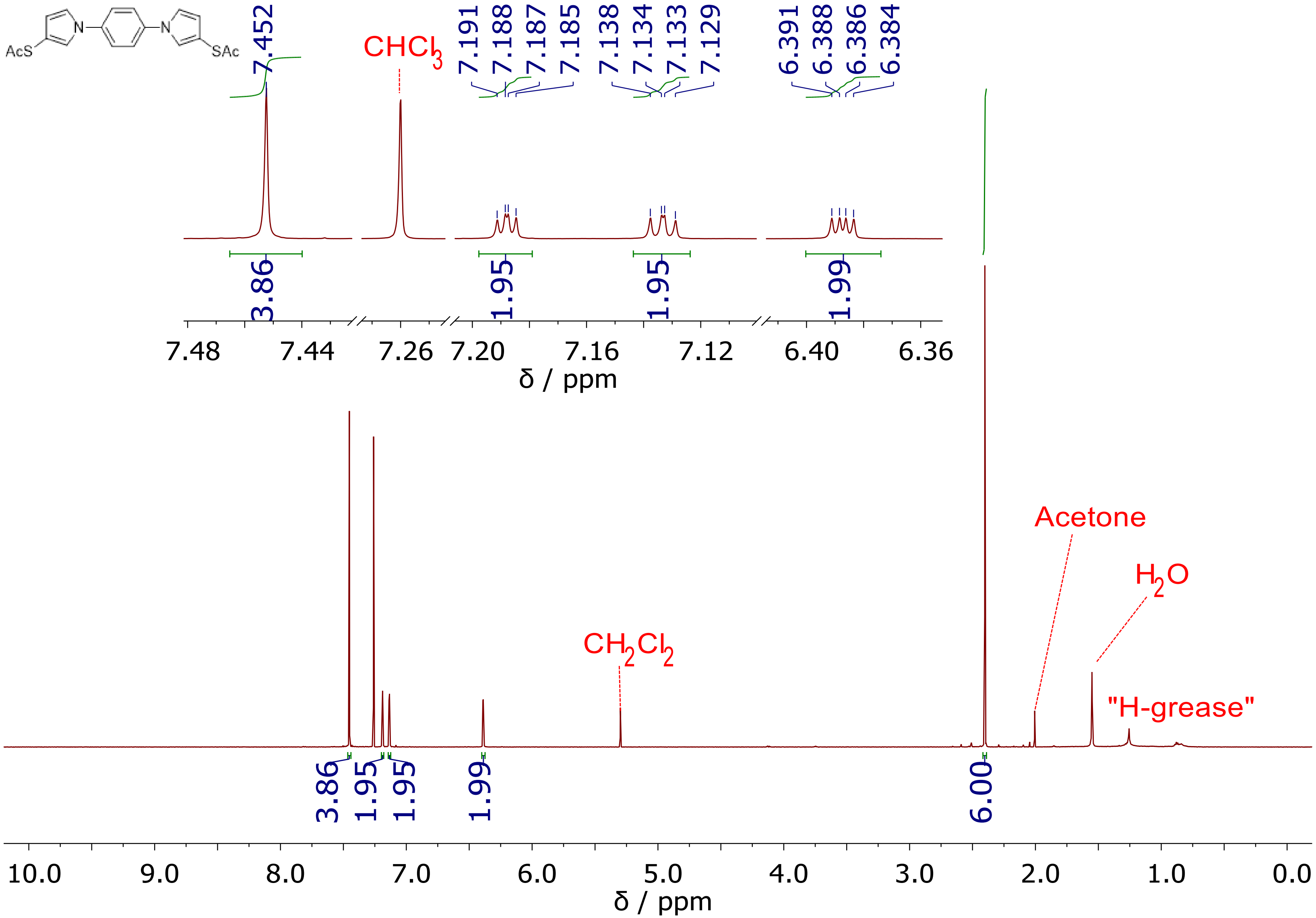

Figure S7. 600 MHz ^{1}H NMR spectrum of compound **1-SAc** in $CDCl_3$.

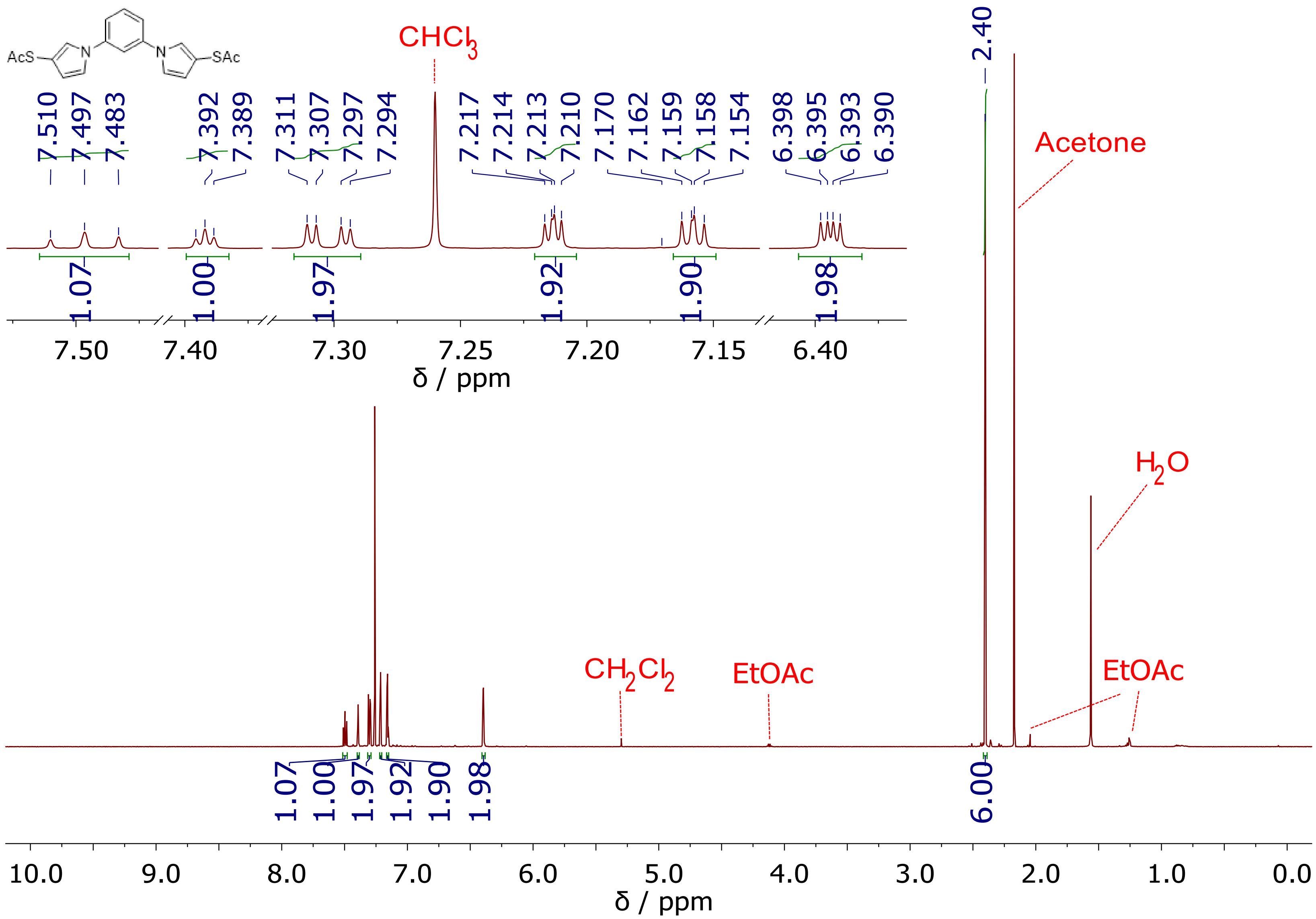

Figure S8. 600 MHz ^{1}H NMR spectrum of compound **2-SAc** in $CDCl_3$.

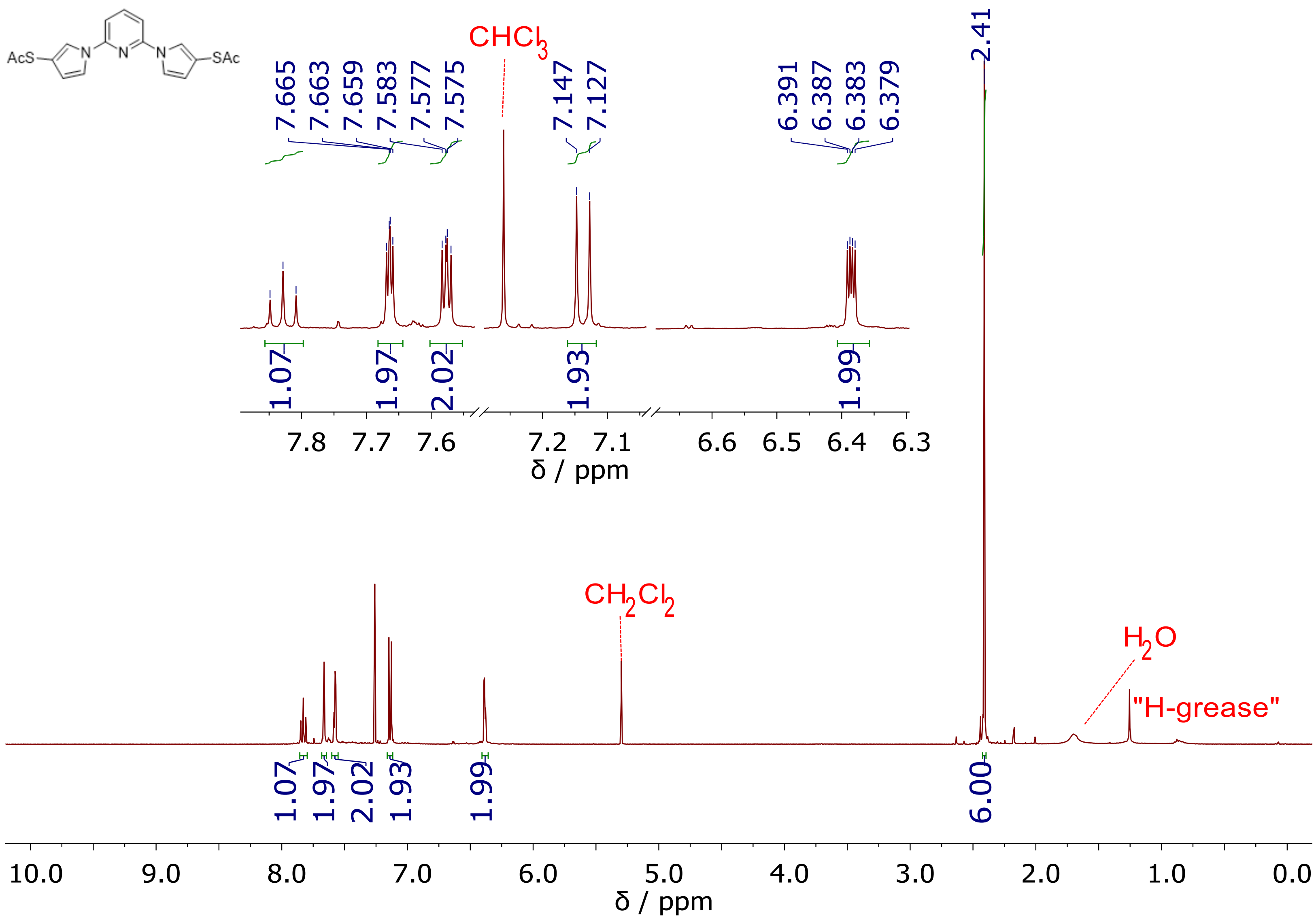

Figure S9. 400 MHz ^{1}H NMR spectrum of compound **3-SAc** in $CDCl_3$.

2.4: ^{13}C NMR spectra of compounds used in conductance studies

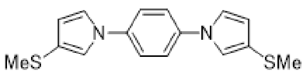

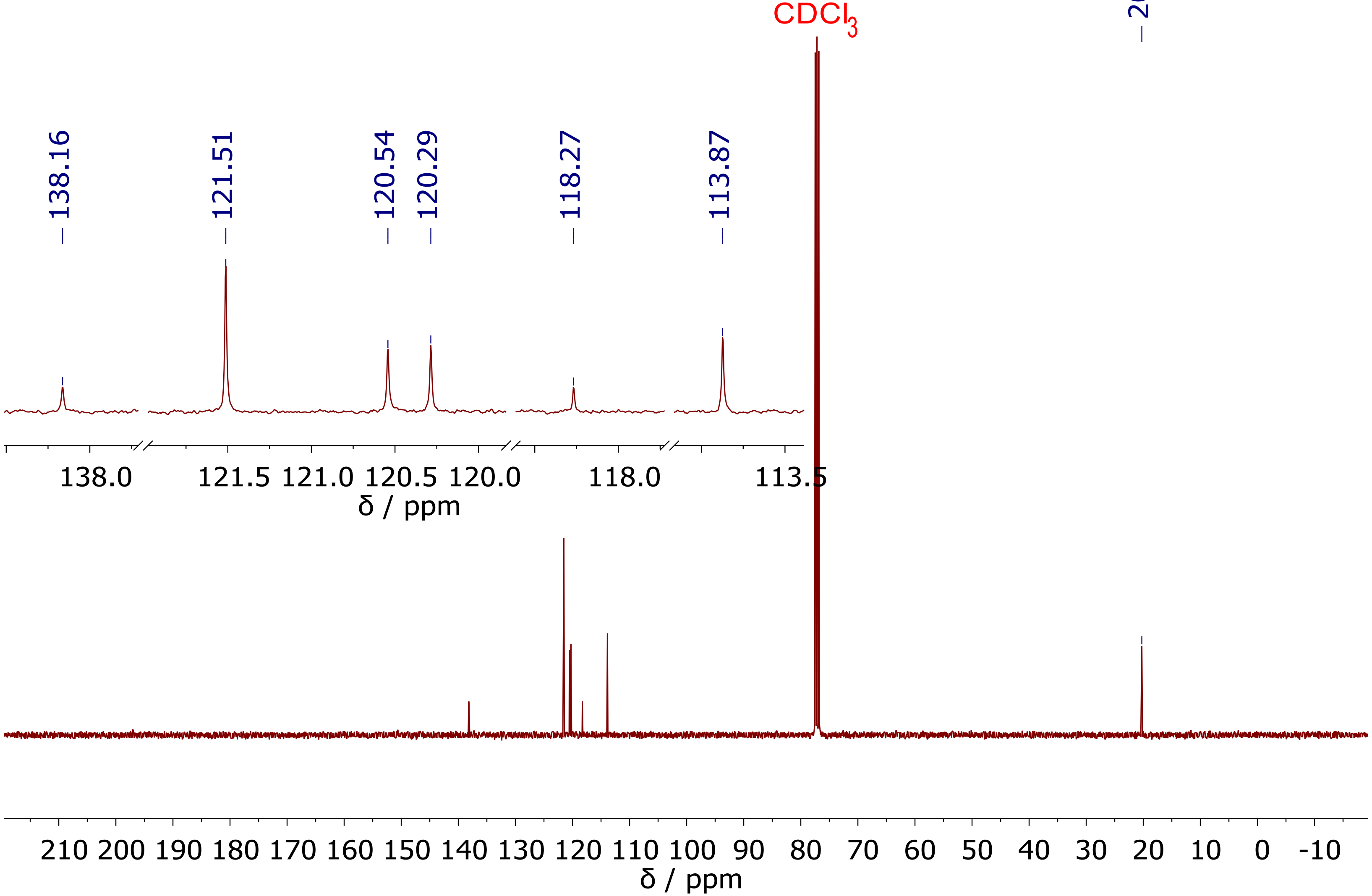

Figure S10. 100 MHz ^{13}C NMR spectrum of compound **1-SMe** in $CDCl_3$.

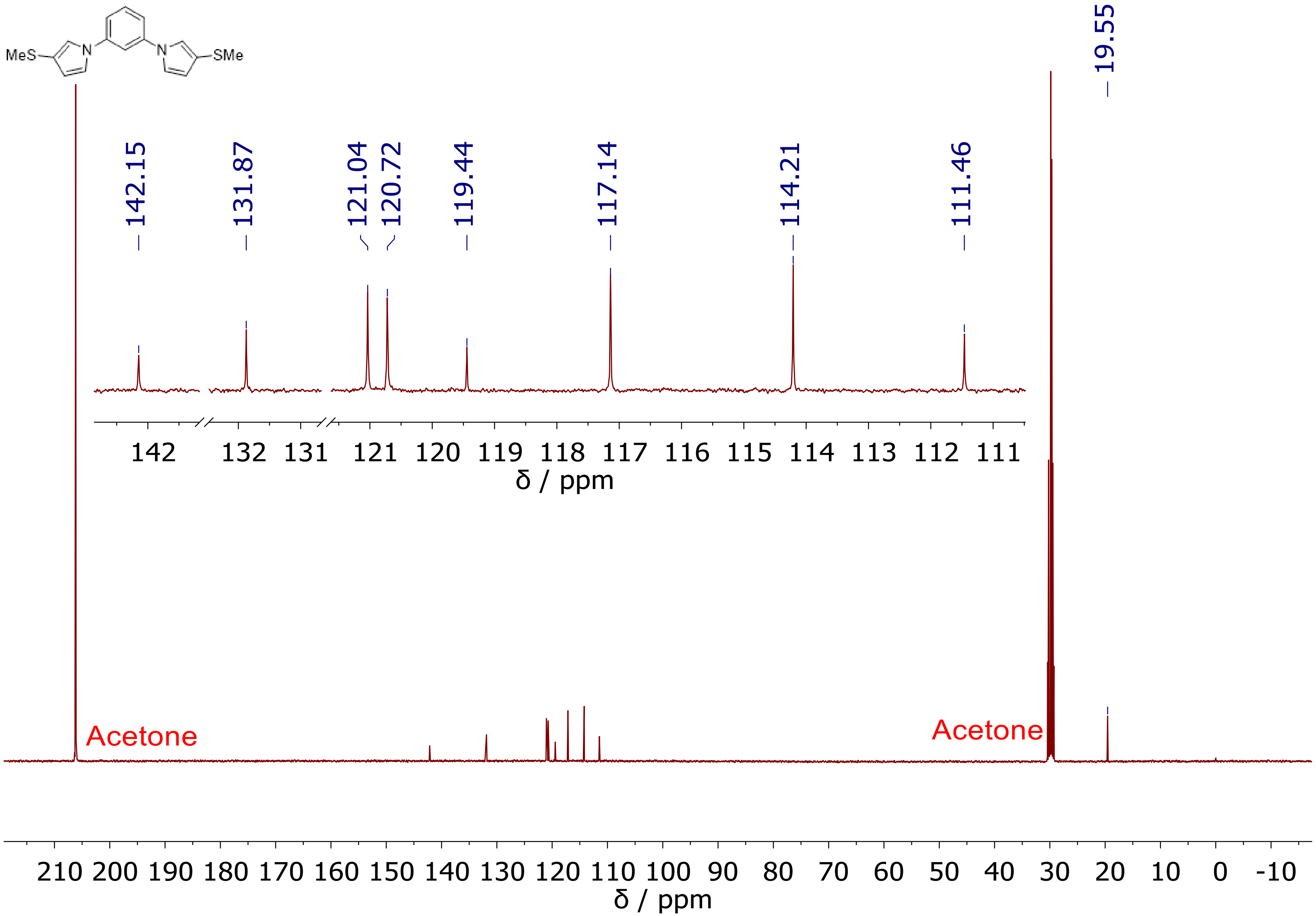

Figure S11. 100 MHz ^{13}C NMR spectrum of compound **2-SMe** in $CD_3C(O)CD_3$.

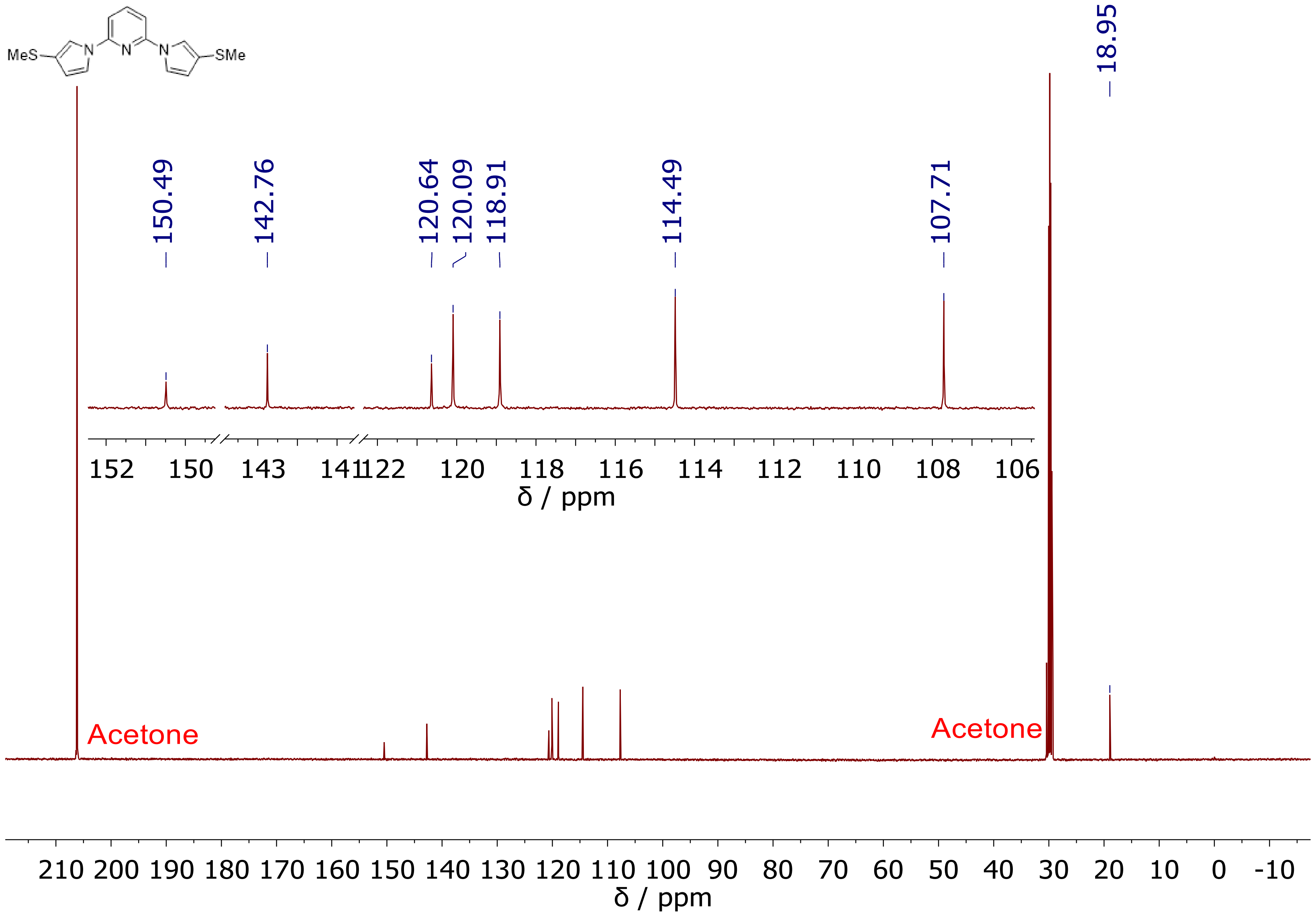

Figure S12. 100 MHz ^{13}C NMR spectrum of compound **3-SMe** in $CD_3C(O)CD_3$.

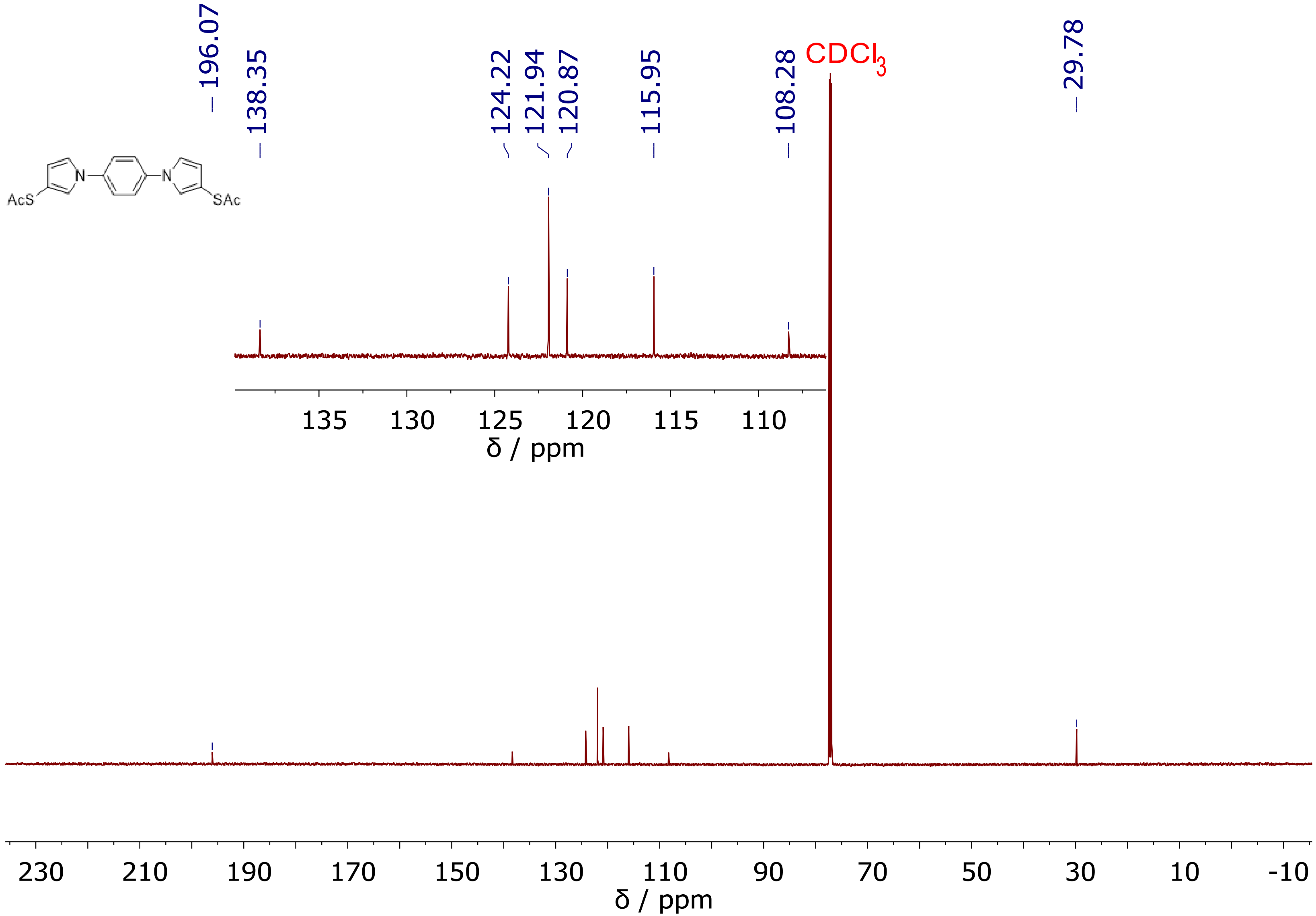

Figure S13. 150 MHz ^{13}C NMR spectrum of compound **1-SAc** in $CDCl_3$.

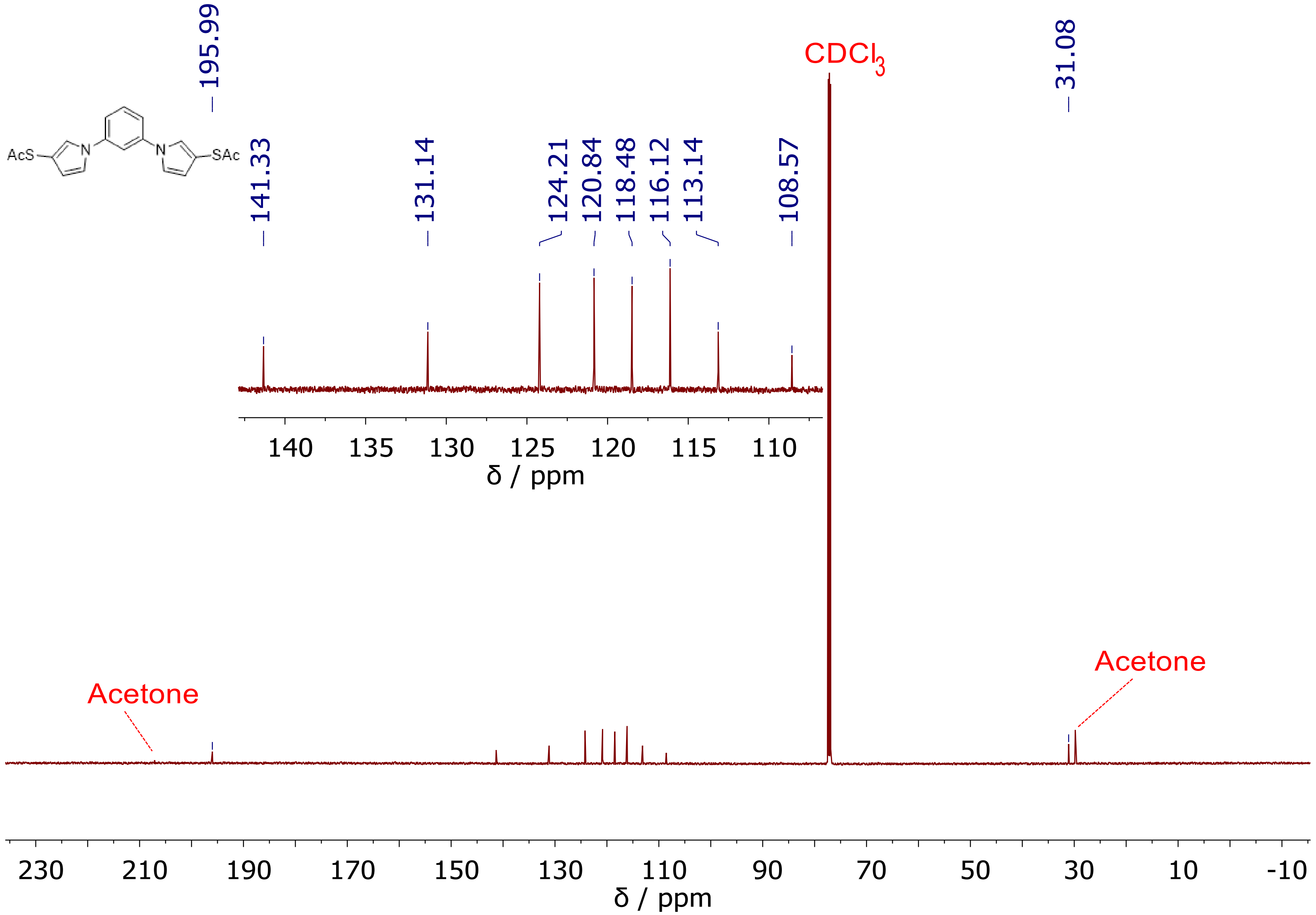

Figure S14. 150 MHz ^{13}C NMR spectrum of compound **2-SAc** in $CDCl_3$.

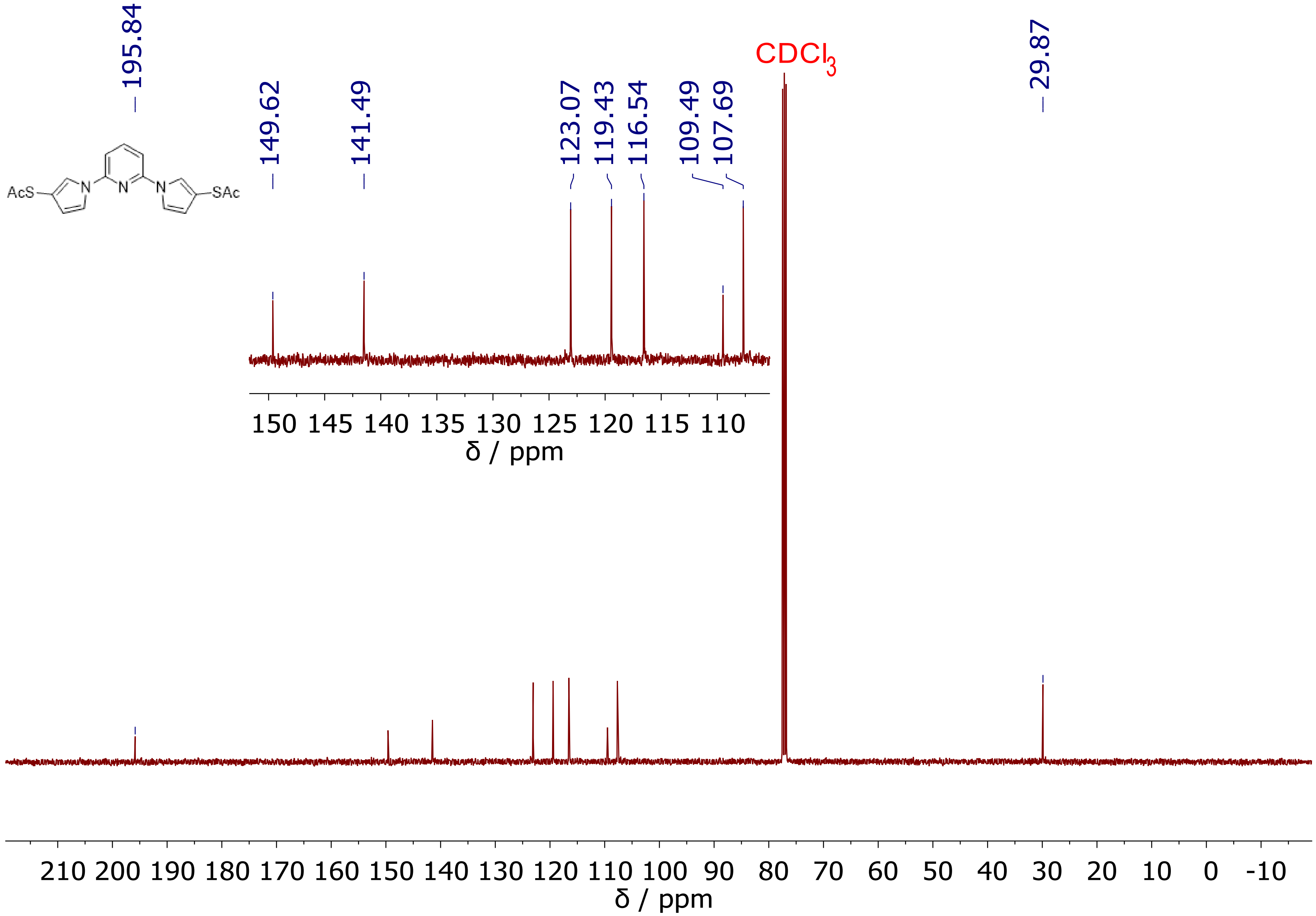

Figure S15. 100 MHz ^{13}C NMR spectrum of compound **3-SAc** in $CDCl_3$.

2.5: HRMS Data

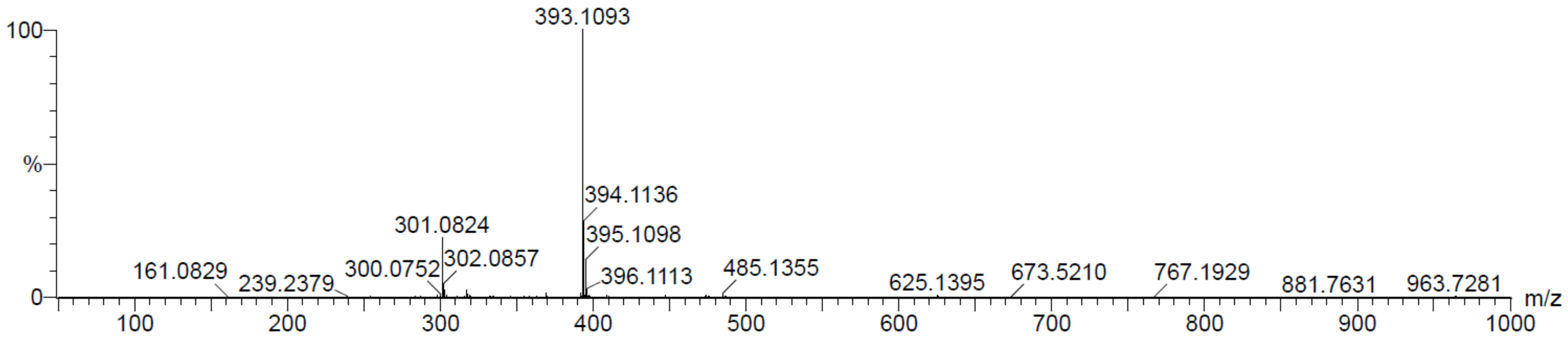

Figure S16. HRMS ASAP-TOF spectrum for **1-SMe** with molecular ion ([M+H]$^+$) peak at m/z = 301.0824.

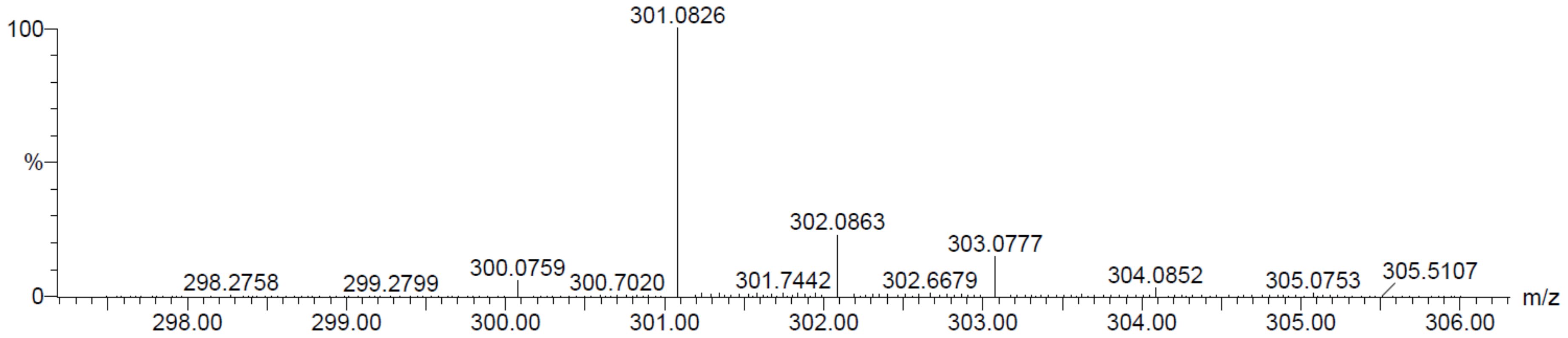

Figure S17. HRMS ASAP-TOF spectrum for **2-SMe** with molecular ion ([M+H]$^+$) peak at m/z = 301.0826.

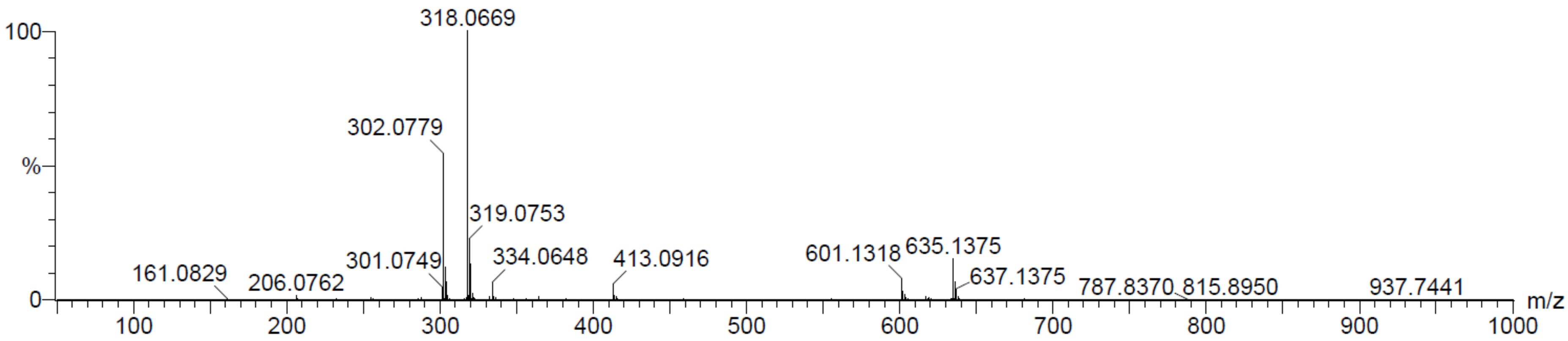

Figure S18. HRMS ASAP-TOF spectrum for **3-SMe** with molecular ion ([M+H]$^+$) peak at m/z = 302.0779.

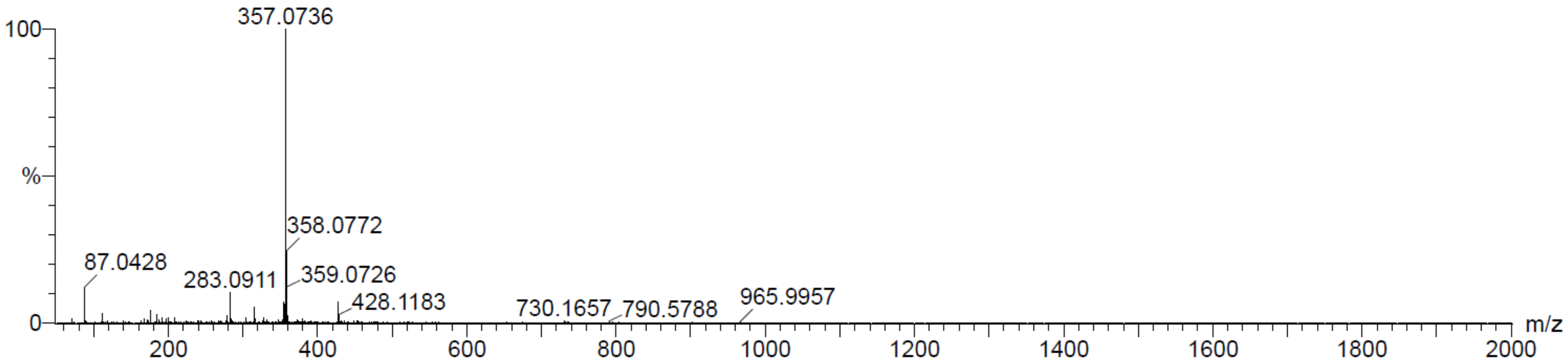

Figure S19. HRMS ESI-TOF spectrum for **1-SAc** with molecular ion ([M+H]$^+$) peak at m/z = 357.0736.

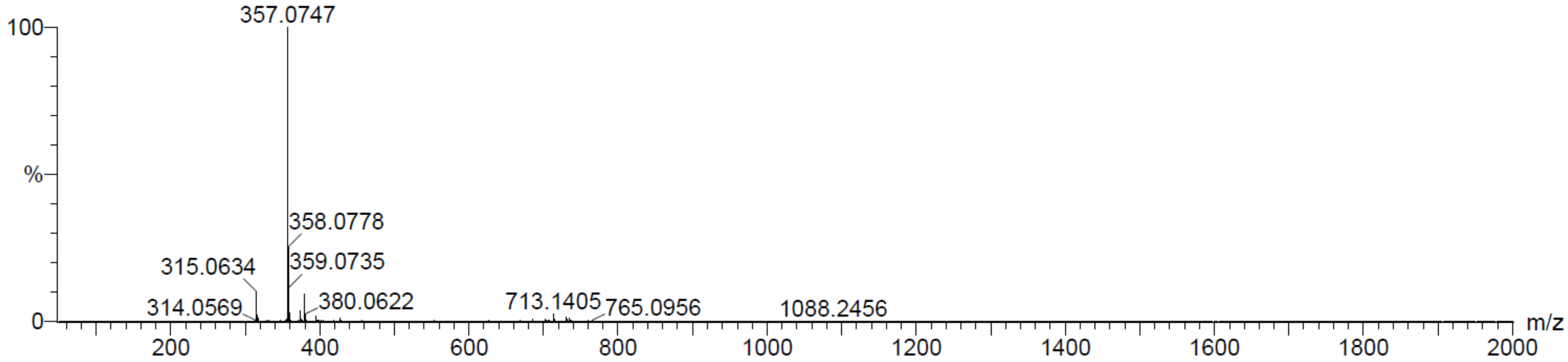

Figure S20. HRMS ESI-TOF spectrum for **2-SAc** with molecular ion ($[M+H]^+$) peak at m/z = 357.0747.

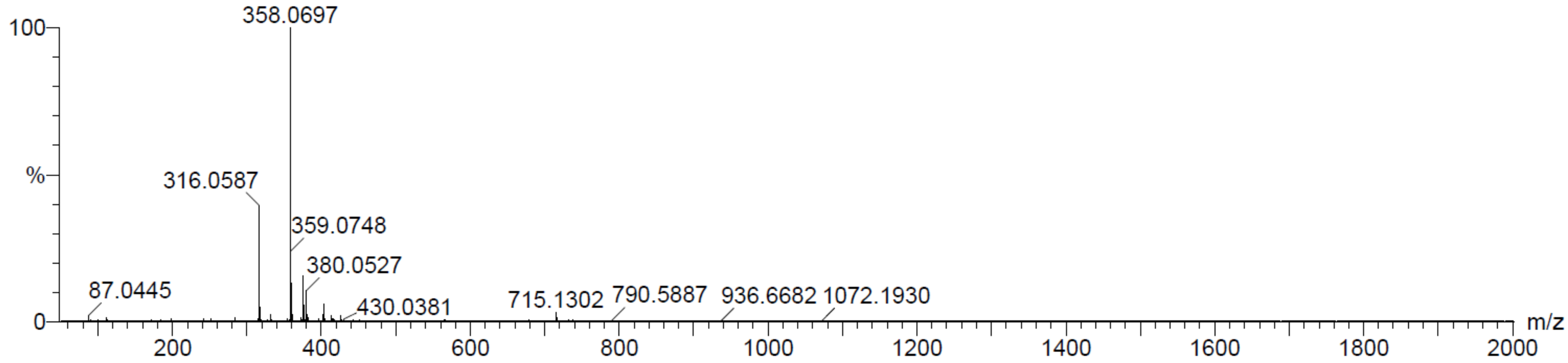

Figure S21. HRMS ESI-TOF spectrum for **3-SAc** with molecular ion ($[M+H]^+$) peak at m/z = 358.0697.

2.6: UV-visible spectra of molecular wires 1-SMe, 2-SMe, 3-SMe, 1-SAc, 2-SAc and 3-SAc

Figure S22 shows UV-visible absorption spectra for the six molecular wires studied. The species bearing thiomethyl anchors (**n-SMe**) all have an absorbance maximum at a longer wavelength than their thioacetate analogs (**n-SAc**). This red shift is indicative of increased conjugation in the thiomethyl species relative to the thioacetates. This can be rationalized by increased electron density in the pyrrole ring of the thiomethyl species versus the thioacetates, as the SMe sulfur lone pair can delocalize only into the pyrrole ring system whereas the SAc sulfur lone pair can also be delocalized into the carbonyl of the acetate group, making it a poorer electron donor. A more electron-rich pyrrole ring is better able to donate electron density into the central six-membered ring system to form an extended conjugated system. This donor-acceptor interaction is supported by the red-shifted absorption maximum of the pyridine derivatives **3-X** relative to their benzene analogs **2-X**. Pyridine is a better acceptor group than benzene due to its electronegative nitrogen atom, and therefore would be expected to better withdraw electrons from the neighboring pyrrole ring, favoring extended conjugation and a red-shifted absorption. Conceptually, these are the same electron delocalizations illustrated when applying ECAR-2 in Figure S1-S3.

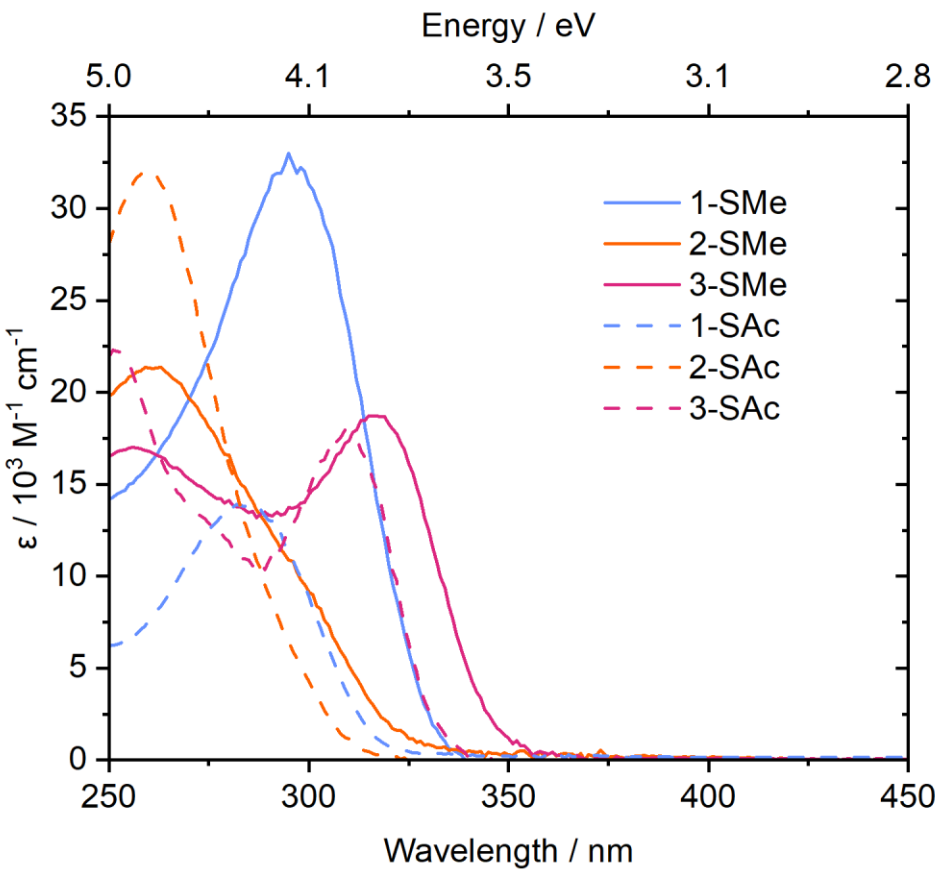

Figure S22. UV-visible absorption spectra of **1-SMe**, **2-SMeb 3-SMe**, **1-SAc**, **2-SAc** and **3-SAc** in CH_2Cl_2. Wires with the same backbone have lines of the same color, wires with the same anchor group have the same line style (solid or dash).

3. SINGLE MOLECULE CONDUCTANCE EXPERIMENTS

3.1: Experimental methods and discussion

The calculation of the junction length was based on a previously reported method. [17] In brief, the junction displacements were calculated by correcting the piezo stretching rate and tip snapback distance. Firstly, we used the pure mesitylene or trichlorobenzene solvent as a blank experiment (Figure S23) and determined the piezo stretching rate by calibrating the direct tunneling distance distribution in the solvent relative to the reported value (range from $10^{-3.5}$ G_0 to $10^{-5.5}$ G_0, 0.36 nm). We then used the determined piezo stretching rate to analyze the statistical junction displacements. The final junction displacements were obtained by adding the snapback distance (0.5 ± 0.1 nm). [17] The distinct configurations of molecular junctions and their corresponding statistical plateau lengths are presented in Figure S25. Experimental results show that the plateau lengths of the molecules fall in the range of 0.6-0.9 nm, while the final junction displacements obtained after calibration are in the range of 1.1-1.4 nm. The small length variation observed for similar molecules is likely attributable to measurement variation induced by the different conformations of the single molecule within the molecular junction.

Flicker noise measurements and subsequent data analysis were performed following established protocols from prior literature.[18, 19] This strategy allows for the identification of charge transport mechanisms in single-molecule junctions by characterizing electrical fluctuations induced by flicker noise within the junctions. Specifically, conductance data were recorded in static mode over a 100 ms time window, and the corresponding power spectral density (PSD) was derived via Fourier transform. The PSD was then integrated across the low-frequency range (100 Hz to 1000 Hz), and normalization of the integrated PSD signal yielded the noise power. A positive correlation between noise power and average junction conductance is indicative of through-space charge transport, where electron transmission proceeds via spatial tunneling. In contrast, a negative correlation between these two parameters classifies the transport behavior as through-bond, in which electron transfer is mediated by the molecular chemical bonding framework. For further quantitative characterization of the transport mechanism, noise power was iteratively normalized against successive integer powers of the average conductance; this normalization process continued until no discernible correlation was observed between the normalized noise power and average conductance. The critical exponent N at which this correlation vanishes acts as a key metric to evaluate the dominant charge transport type: an N value approaching 1 points to preferential through-bond transport, while a value near 2 indicates a predominance of through-space transport in the single-molecule junction. The flicker noise analysis for single-molecule junctions containing **1-S**, **2-S** and **3-S** is shown in Figure S26. In all cases, the value of critical exponent N is closer to 1 than 2, indicative of through-bond transport as the predominant charge transport pathway in these systems.

3.2: Supplementary experimental figures

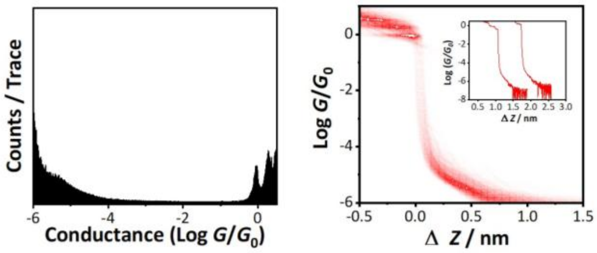

Figure S23: Traces from blank STM-BJ experiments in pure trichlorobenzene.

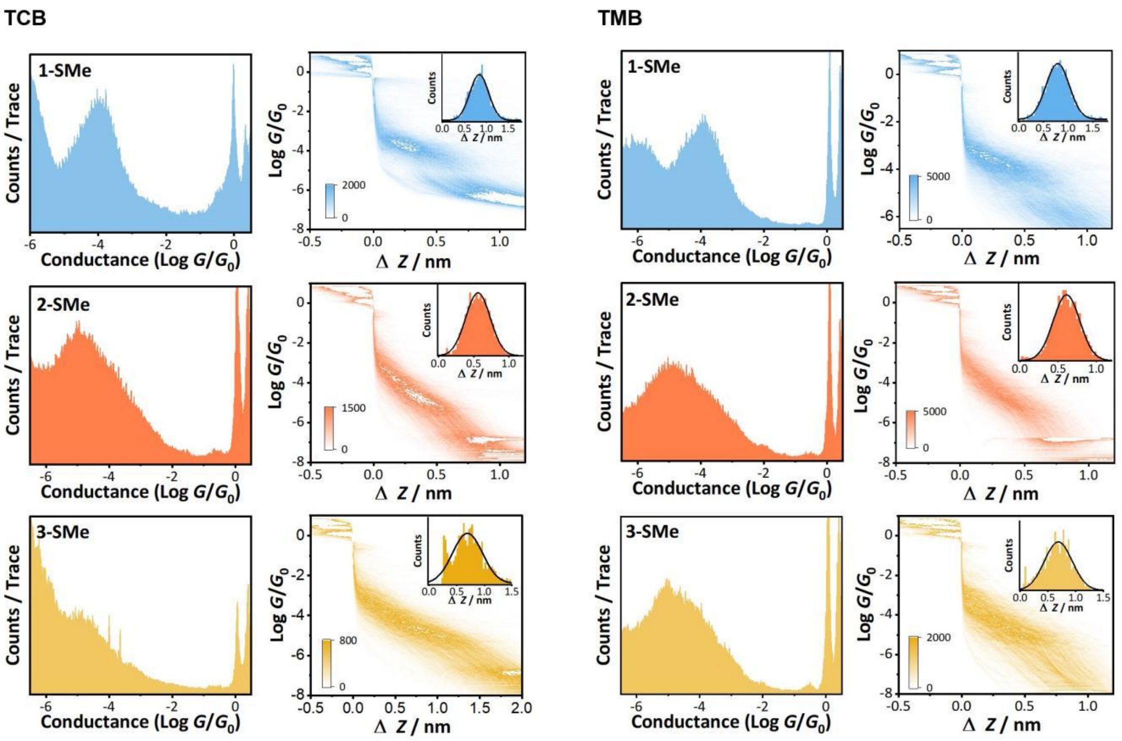

Figure S24. Left: Conductance histograms obtained for **1-SMe**, **2-SMe** and **3-SMe** in trichlorobenzene (TCB). Second from left: 2D conductance-displacement histograms for **1-SMe**, **2-SMe** and **3-SMe** in trichlorobenzene (TCB). Third from left: Conductance histograms obtained for **1-SMe**, **2-SMe** and **3-SMe** in trimethylbenzene (TMB, mesitylene). Right: 2D conductance-displacement histograms for **1-SMe**, **2-SMe** and **3-SMe** in trimethylbenzene (TMB, mesitylene).

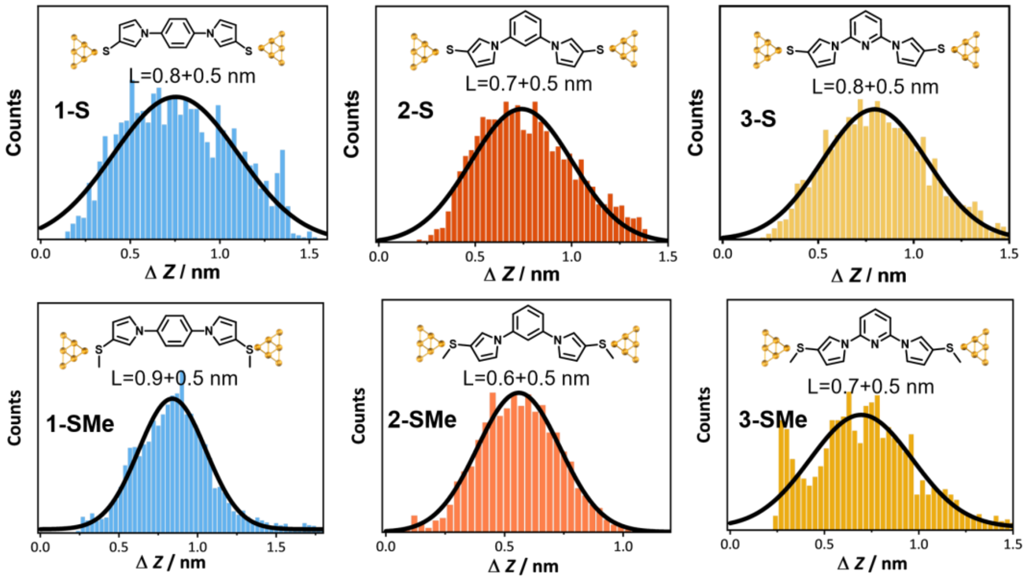

Figure S25. The experimental plateau lengths for the molecules in trichlorobenzene (TCB).

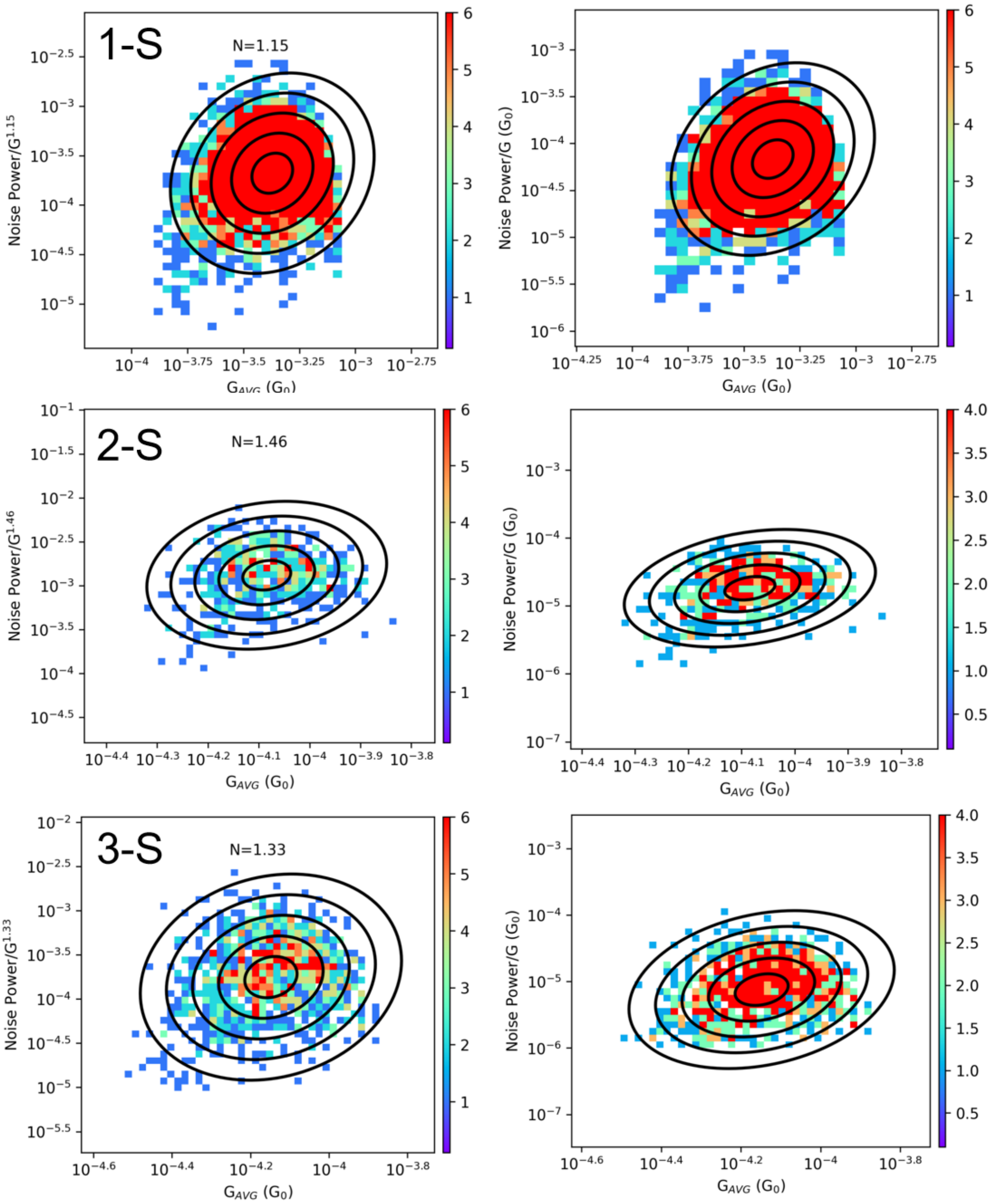

Figure S26. The flicker noise distribution analysis of the single-molecule junctions (**1-S**, **2-S** and **3-S**).

4. COMPUTATIONAL METHODS

4.1: Orbital analysis

4.1.1: Basic equations

Green's function is the impulse response of the Schrödinger equation which carries all information regarding wavefunction evolution from one point to the other in a single-molecule junction system.[20] The Green's function g of a system with N sites described by Hamiltonian H can be written in terms of eigenstates ψ_n and eigenenergies λ_n of H:

$$g = \sum_{n=1}^{N} \frac{|\psi_n\rangle\langle\psi_n|}{E-\lambda_n} \quad (1)$$

where λ_n correspond to the eigenvalues.

Molecular orbitals (i.e., wavefunctions) can be used to predict molecules' DQI and CQI. Consider the wavefunction for energy level E_n at site i is $\psi_n(i)$. QI is expected to be destructive in the middle of the HOMO-LUMO gap, $E_{HL} = (E_L + E_H)/2$ if:

$$T(E) \propto g = \frac{\psi_H(i)\psi_H^*(j)}{E_{HL}-E_H} + \frac{\psi_L(i)\psi_L^*(j)}{E_{HL}-E_L} \quad (2)$$

where g is the Green's function and ψ_H and ψ_L are the HOMO and LUMO wavefunctions, respectively. Since $E_{HL} - E_H > 0$ and $E_{HL} - E_L < 0$, Equation (2) will only be satisfied if the sign of $\psi_H(i)\psi_H^*(j)$ is the same as the sign of $\psi_L(i)\psi_L^*(j)$.

If $|\psi_H(i)\psi_H^*(j)| \ll |\psi_L(i)\psi_L^*(j)|$, then $E \simeq E_H$ and the DQI dip lies close to the HOMO. If the HOMO or/and LUMO is degenerate, then the additional degenerate orbitals need to be considered. For example, when the HOMO is degenerate, Equation (2) reads as:

$$T(E) \propto g = \frac{\psi_{H-1}(i)\psi_{H-1}^*(j)+\psi_H(i)\psi_H^*(j)}{E_{HL}-E_H} + \frac{\psi_L(i)\psi_L^*(j)}{E_{HL}-E_L} \quad (3)$$

Conversely, when the LUMO is degenerate, Equation (2) reads as:

$$T(E) \propto g = \frac{\psi_H(i)\psi_H^*(j)}{E_{HL}-E_H} + \frac{\psi_L(i)\psi_L^*(j)+\psi_{L+1}(i)\psi_{L+1}^*(j)}{E_{HL}-E_L} \quad (4)$$

Based on the observation that DQI is predicted only when Equation (2) is satisfied, i.e. when the signs of $\psi_H(i)\psi_H^*(j)$ and $\psi_L(i)\psi_L^*(j)$ are the same (and hence CQI is predicted when the signs of these products are different), it is possible to make QI predictions based on visual analysis of molecular orbitals. By considering whether the phase of each of HOMO and LUMO is the same or different at the two points at which anchor groups are attached to the molecular backbone, QI behaviour can be predicted as follows: if the phase of only one of the HOMO and LUMO differs between anchor group attachment points, CQI is expected. If either both the HOMO and LUMO have the same phase at each anchor group attachment point, or both have different phases at each attachment point, DQI is expected.

4.1.2: DFT orbital analysis

Figure S27 shows the molecular orbitals and energy levels from HOMO-3 to LUMO+3 for the molecular cores of interest, **1**, **2**, and **3** (i.e. the central portion of the molecules synthesized and measured experimentally, with their sulfur-based anchoring groups replaced by hydrogen).

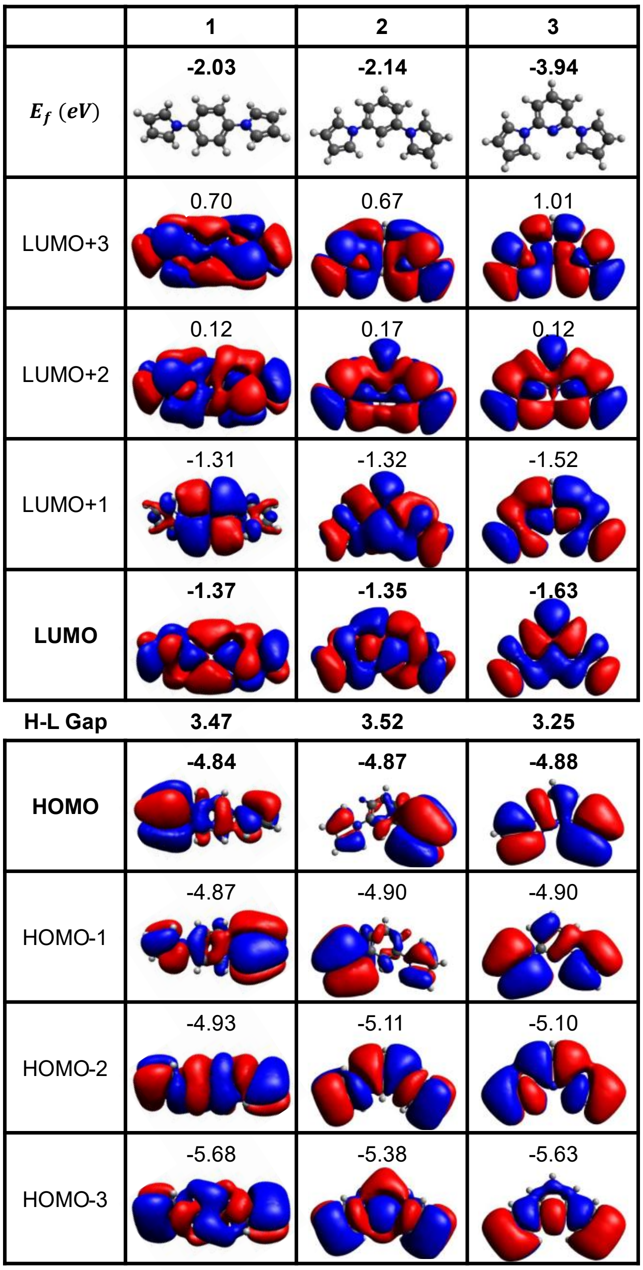

Figure S27: Molecular orbitals from HOMO-3 to LUMO+3 for molecular cores **1**, **2**, and **3**.

The HOMO and HOMO-1 of all three molecular cores are found to have almost identical energies ($\Delta E \cong \pm 0.03\ eV$) in these calculations, i.e., they are degenerate. Both the HOMO and HOMO-1 must therefore both be considered when carrying out orbital analysis due to degeneracy, according to Equation (3). Furthermore, the LUMO and LUMO+1 of molecular core **2** are also degenerate ($\Delta E \cong \pm 0.03\ eV$) and should therefore additionally be considered in orbital analysis of this core (c.f. Equation (4)); the energy difference between the LUMO and LUMO+1 of molecular core **1** is only slightly larger ($\Delta E \cong \pm 0.06\ eV$) so the effects of degeneracy may also be relevant in this case. As a result of this degeneracy, prediction of QI behavior based on orbital analysis of these systems becomes ambiguous. Analysis is further complicated by the chemical equivalence of the pyrrole 3- and 4-positions, as the selection of which is used as the anchoring point for each ring can afford different predictions.

Further orbital analysis was carried out on molecules including anchoring units to afford more definite predictions of QI behavior. This makes the location of the anchoring units definite and additionally

accounts for any effects of neighboring orbitals on the anchor groups on electron transmission. Figure S28 shows the molecular orbitals and energy levels from HOMO-3 to LUMO+3 for **1-SMe**, **2-SMe**, **3-SMe**, **1-SH**, **2-SH**, and **3-SH**.

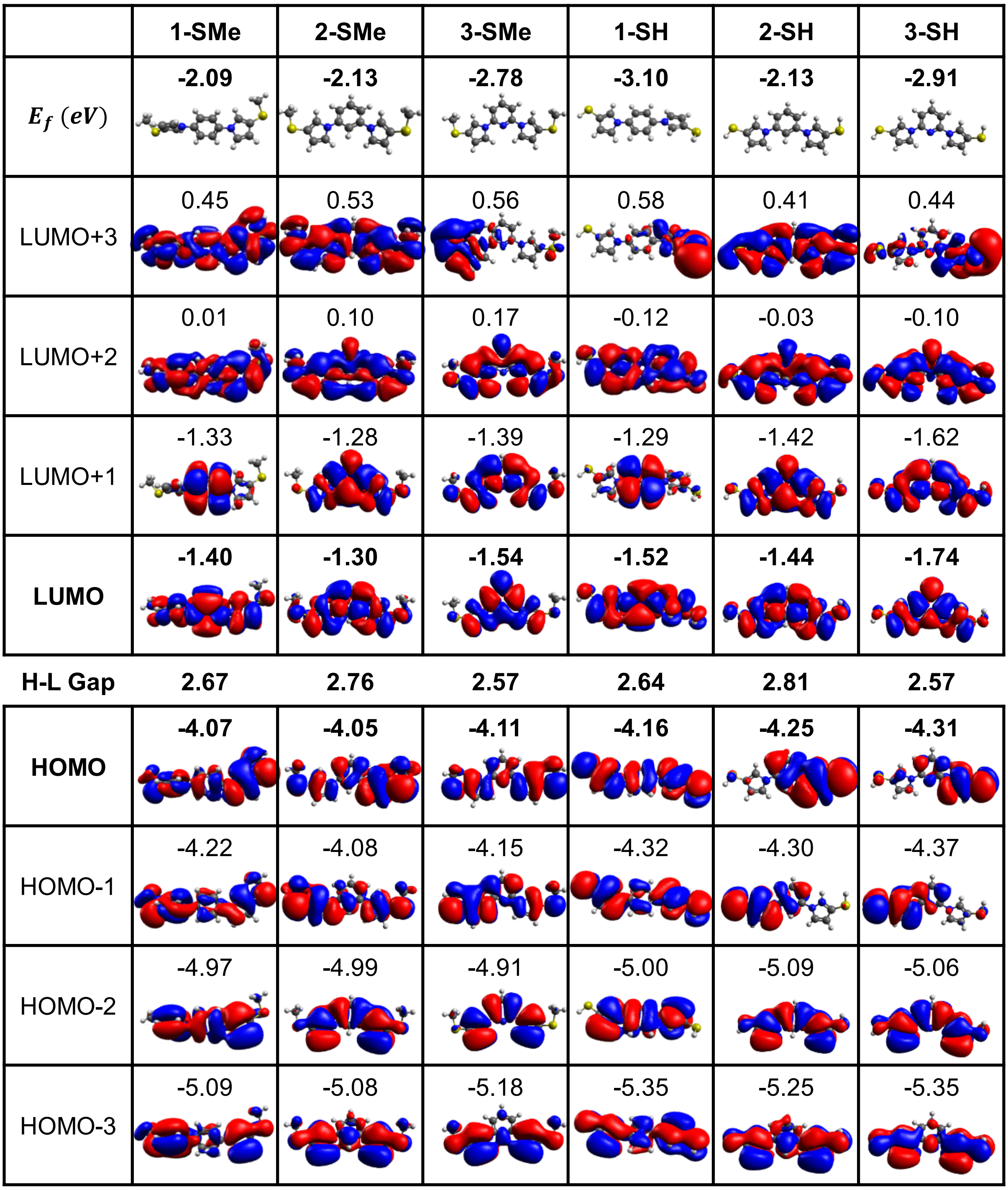

	**1-SMe**	**2-SMe**	**3-SMe**	**1-SH**	**2-SH**	**3-SH**
E_f (eV)	**-2.09**	**-2.13**	**-2.78**	**-3.10**	**-2.13**	**-2.91**
LUMO+3	0.45	0.53	0.56	0.58	0.41	0.44
LUMO+2	0.01	0.10	0.17	-0.12	-0.03	-0.10
LUMO+1	-1.33	-1.28	-1.39	-1.29	-1.42	-1.62
**LUMO**	**-1.40**	**-1.30**	**-1.54**	**-1.52**	**-1.44**	**-1.74**
**H-L Gap**	**2.67**	**2.76**	**2.57**	**2.64**	**2.81**	**2.57**
**HOMO**	**-4.07**	**-4.05**	**-4.11**	**-4.16**	**-4.25**	**-4.31**
HOMO-1	-4.22	-4.08	-4.15	-4.32	-4.30	-4.37
HOMO-2	-4.97	-4.99	-4.91	-5.00	-5.09	-5.06
HOMO-3	-5.09	-5.08	-5.18	-5.35	-5.25	-5.35

Figure S28: Molecular orbitals from HOMO-3 to LUMO+3 for **1-SMe**, **2-SMe**, **3-SMe**, **1-SH**, **2-SH**, and **3-SH**.

The inclusion of the anchoring groups in the MO calculations means that clear degeneracy of the HOMO and/or LUMO is no longer observed in some cases, i.e. **1-SMe** (taking $\Delta E \cong 0.07\ eV$ for the LUMO as a sufficient difference for non-degeneracy) and **1-SH**. The HOMO and HOMO-1 are degenerate for **2-SMe**, **3-SMe** and **2-SH** ($\Delta E \leq \pm 0.05\ eV$) and possibly **3-SH** ($\Delta E \cong \pm 0.06\ eV$), while the LUMO and LUMO+1 are degenerate for **2-SMe** and **2-SH** ($\Delta E \cong \pm 0.02\ eV$). As such, in most cases,

applying orbital analysis for these degenerate orbitals will again yield ambiguous QI predictions, suggesting that the orbital analysis method is not well-suited to the present systems. It is possible to predict CQI for **1-SMe** and **1-SH** (assuming non-degeneracy in the former case) and it is notable that the contribution from the LUMO+1 orbitals to transport for the **2-SR** systems is expected to be constructive, agreeing with the observation of a washed out antiresonance in the transmission functions of these systems (Figures 4 and Figure **S34**b), particularly when comparing **2-S** to **3-S**.

4.2: Magic ratio rule and M-theory for heteroatoms

4.2.1: Brief description

The magic ratio rule (MRR)[21, 22] has been used to capture the influence of connectivity on the conductance ratios of benzene and graphene-like polycyclic aromatic hydrocarbon (PAH) cores such as naphthalene, anthracene, and anthanthrene when the coupling to the electrodes is weak and the Fermi energy lies at the center of the HOMO–LUMO gap. The single-molecule electrical conductance σ_{ij} depends on the choice of connecting atoms i,j.

To understand the effect of introducing a heteroatom like nitrogen into a PAH core, the PAH core can be treated as a parent and the heteroatom-substituted core as a daughter. The conductance relationship can be summarized by M-theory[23] which shows: (a) when i and j are both odd, both parent and daughter have low conductances, (b) when i is odd and j is even, or vice versa, both parent and daughter have high conductances and (c) when i and j are both even, the parent has a low conductance and the daughter a high conductance. The key conceptual and mathematical steps that underline the MRR and associated M-theory governing the heteroatom effect are explained thoroughly in the supplementary information of previously published work. [3]

4.2.2: 6-membered molecular core

Figure S29 illustrates that the connecting atoms on the parent benzene cores of **1-X** molecules are at sites 2 & 5 (even-odd), and for **2-X** at sites 2 & 6 (even-even). The **3-X** series can be considered the daughter of the **2-X** series as they both have the same 2,6 connectivity (even-even) but the pyridine core of **3-X** adds a nitrogen heteroatom at site 1.

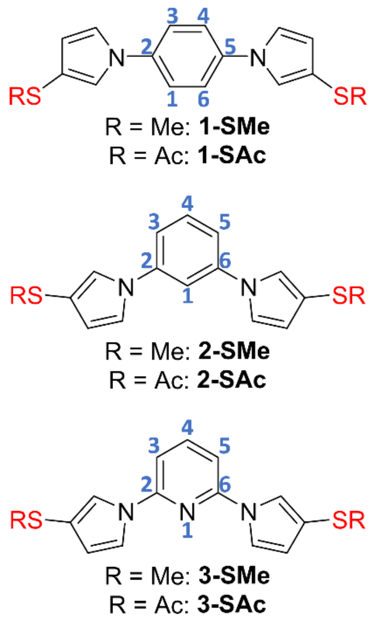

Figure S29: A convenient numbering of the central parent benzene and daughter pyridine cores of the studied molecules for M-theory analysis.

To obtain the M-table for a given molecular core, a connectivity table must first be constructed to show the Hamiltonian H, with rows and columns labelled by the site number i and j, respectively, such that entry H_{ij} is −1 if sites i and j are connected, and zero otherwise. Table S2 shows the Hamiltonian H^p of a parent benzene core.

Table S2: Connectivity table showing the Hamiltonian $\boldsymbol{H^p}$ of parent benzene core.

$\boldsymbol{H^p}$	**1**	**3**	**5**	**2**	**4**	**6**
**1**	0	0	0	-1	0	-1
**3**	0	0	0	-1	-1	0
**5**	0	0	0	0	-1	-1
**2**	-1	-1	0	0	0	0
**4**	0	-1	-1	0	0	0
**6**	-1	0	-1	0	0	0

The corresponding M-table M is defined to be the transpose of the cofactor matrix of H. Table S3 is the M-table $M^p(0)$ at mid-gap ($E = 0$) corresponding to H^p in which odd-numbered sites are connected to even numbered sites only. Connection at site 2 & 5 (2 & 6) has a M integral of −2 (0); this means the conductance of **1-X** series molecules is expected to be higher than the corresponding **2-X** series molecules.

Table S3: The M-table $\boldsymbol{M(0)}$ at mid-gap ($\boldsymbol{E = 0}$) corresponding to $\boldsymbol{H^p}$.

$\boldsymbol{M^p(0)}$	**1**	**3**	**5**	**2**	**4**	**6**
**1**	0	0	0	2	-2	2
**3**	0	0	0	2	2	-2
**5**	0	0	0	-2	2	2
**2**	2	2	-2	0	0	0
**4**	-2	2	2	0	0	0
**6**	2	-2	2	0	0	0

Table S4 shows the Hamiltonian H^d of a daughter pyridine core accounting for the presence of a heteroatom at site 1, with a nitrogen non-zero on-site energy ε_N.

Table S4: The daughter pyridine core Hamiltonian $\boldsymbol{H^d}$ in the presence of a heteroatom on site 1, with a nitrogen non-zero on-site energy $\boldsymbol{\varepsilon_N}$.

$\boldsymbol{H^d}$	**1**	**3**	**5**	**2**	**4**	**6**
**1**	$\boldsymbol{\varepsilon_N}$	0	0	-1	0	-1
**3**	0	0	0	-1	-1	0
**5**	0	0	0	0	-1	-1
**2**	-1	-1	0	0	0	0
**4**	0	-1	-1	0	0	0
**6**	-1	0	-1	0	0	0

Table S5 is the M-table $M^d(0)$ at mid-gap corresponding to H^d. Provided that the Fermi energy of the daughter coincides with the mid-gap of the parent, the effect of the heteroatom substitution onto an odd-numbered site is captured by M-theory. Without assigning a value to ε_N, the fact that it is a non-zero value will result in an enhancement to the transmission coefficient and lead to higher conductance for a wide range of Fermi energies.

Table S5: The M-table $\boldsymbol{M^p(0)}$ corresponding to $\boldsymbol{H^d}$.

$\boldsymbol{M^d(0)}$	**1**	**3**	**5**	**2**	**4**	**6**
**1**	0	0	0	2	-2	2
**3**	0	0	0	2	2	-2
**5**	0	0	0	-2	2	2
**2**	2	2	-2	ε_N	-ε_N	ε_N
**4**	-2	2	2	-ε_N	ε_N	-ε_N
**6**	2	-2	2	ε_N	-ε_N	ε_N

By using M-theory on the molecular cores, the following preliminary predications can be made before any computational calculations: (a) **1-R** series parent molecules are expected to have a higher conductance than both the corresponding **2-R** series parent and **3-R** series daughter molecules, (b) **3-R** series daughter molecules are expected to have a higher conductance than the corresponding **2-R** series parent molecules, but this magnitude of this difference is dependent on the value of ε_N, (c) If we assign $\varepsilon_N = 0.18$ to match the tight binding (TB) models, then the M integral for the **3-R** series daughter molecules is -0.18. As the modulus of the M integral for the different series follows the trend:

1-R (2) >> **3-R** (0.18) > **2-R** (0)

the implication is that for the **1-R** series CQI can be expected whereas DQI can be expected for the **2-R** and **3-R** series, but the conductance of **3-R** is expected to be slightly higher than **2-R**.

Previously, using the same benzene cores but with oligo(aryleneethynylene) (OAE) backbones and thiolate anchors, Liu *et al.* demonstrated that: (a) a *para*-connected OAE with no heteroatom substitution has higher conductance than *meta* derivatives or heteroatom-substituted daughters; (b) a *meta*-connected OAE with a central 2,6-connected pyridine ring has a higher conductance than its parent all-hydrocarbon *meta*-connected OAE. This finding agrees with predictions based on M-theory. [3] Chen *et al.* demonstrated that M-theory predictions still hold true when the 6-membered benzene core is replaced with a 5-membered thiophene ring, i.e. 2,5-connectivity at the central thiophene has a higher conductance than 2,4-connectivity.[24]

4.2.3: Complete molecules

Figure **S30** illustrates that the connecting atoms on the parent **1-X**, **2-X**, and **3-X** molecules are all at site 17 & 18 (odd-even) with nitrogen heteroatoms at sites 7 & 12, and sulfur anchor atoms at sites 17 & 18. The **3-X** series can still be considered the daughter of the **2-X** series with an additional nitrogen heteroatom at site 1. According to M-theory, **1-X**, **2-X**, and **3-X** molecules are all expected to have high conductance.

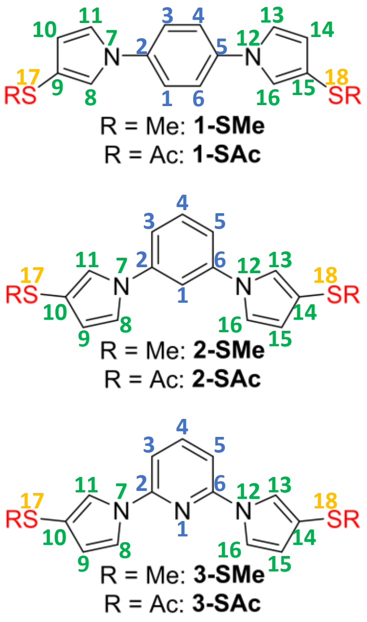

Figure S30: A convenient numbering of the studied full molecules for M-theory analysis.

Table S6 is the connectivity table showing the Hamiltonian H^p of the parent **1-X** molecules in the presence of nitrogen heteroatoms at sites 7 & 12 and sulfur anchor atoms at sites 17 & 18. The nitrogen heteroatoms have a non-zero on-site energy ε_N and the sulfur anchor atoms have a non-zero on-site energy ε_S.

Table S6: Connectivity table showing the Hamiltonian $\boldsymbol{H^p}$ of the parent **1-X** molecules in the presence of nitrogen heteroatoms at sites 7 & 12 and sulfur anchor atoms at sites 17 & 18. The nitrogen heteroatoms have a non-zero on-site energy $\boldsymbol{\varepsilon_N}$ and the sulfur anchor atoms have a non-zero on-site energy $\boldsymbol{\varepsilon_S}$.

$\boldsymbol{H^p}$	**1**	**3**	**5**	**2**	**4**	**6**	**7**	**9**	**11**	**8**	**10**	**13**	**15**	**12**	**14**	**16**	**17**	**18**
**1**	0	0	0	-1	0	-1	0	0	0	0	0	0	0	0	0	0	0	0
**3**	0	0	0	-1	-1	0	0	0	0	0	0	0	0	0	0	0	0	0
**5**	0	0	0	0	-1	-1	0	0	0	0	0	0	0	-1	0	0	0	0
**2**	-1	-1	0	0	0	0	-1	0	0	0	0	0	0	0	0	0	0	0
**4**	0	-1	-1	0	0	0	0	0	0	0	0	0	0	0	0	0	0	0
**6**	-1	0	-1	0	0	0	0	0	0	0	0	0	0	0	0	0	0	0
**7**	0	0	0	-1	0	0	ε_N	0	-1	-1	0	0	0	0	0	0	0	0
**9**	0	0	0	0	0	0	0	0	0	-1	-1	0	0	0	0	0	-1	0
**11**	0	0	0	0	0	0	-1	0	0	0	-1	0	0	0	0	0	0	0
**8**	0	0	0	0	0	0	-1	-1	0	0	0	0	0	0	0	0	0	0
**10**	0	0	0	0	0	0	0	-1	-1	0	0	0	0	0	0	0	0	0
**13**	0	0	0	0	0	0	0	0	0	0	0	0	0	-1	-1	0	0	0
**15**	0	0	0	0	0	0	0	0	0	0	0	0	0	0	-1	-1	0	-1
**12**	0	0	-1	0	0	0	0	0	0	0	0	-1	0	ε_N	0	-1	0	0
**14**	0	0	0	0	0	0	0	0	0	0	0	-1	-1	0	0	0	0	0
**16**	0	0	0	0	0	0	0	0	0	0	0	0	-1	-1	0	0	0	0
**17**	0	0	0	0	0	0	0	-1	0	0	0	0	0	0	0	0	ε_S	0
**18**	0	0	0	0	0	0	0	0	0	0	0	0	-1	0	0	0	0	ε_S

Table S7 is the M-table $M^p(0)$ at mid-gap corresponding to H^p of the parent **1-X** molecules. By assigning non-zero values of $\varepsilon_N = -0.18$ and $\varepsilon_S = -0.6$, an enhancement to the transmission coefficient is seen, leading to higher conductance for a wide range of Fermi energy. Sites 17 and 18 have a M integral of −2.

Table S7: The M-table $\boldsymbol{M^p(0)}$ at mid-gap ($\boldsymbol{E = 0}$) corresponding to $\boldsymbol{H^p}$ of the parent **1-X** molecules in the presence of nitrogen heteroatoms at sites 7 & 12 and sulfur anchor atoms at sites 17 & 18. The nitrogen heteroatoms have an on-site energy $\boldsymbol{\varepsilon_N = -0.18}$ and the sulfur anchor atoms have an on-site energy $\boldsymbol{\varepsilon_S = -0.6}$.

$\boldsymbol{M^p(0)}$	**1**	**3**	**5**	**2**	**4**	**6**	**7**	**9**	**11**	**8**	**10**	**13**	**15**	**12**	**14**	**16**	**17**	**18**
**1**	0.18	0.18	-0.18	0.19	-0.19	-0.17	-0.37	0.37	-0.37	0.25	0.37	0.36	-0.36	0.36	-0.36	-0.24	-0.62	0.60
**3**	0.18	0.18	-0.18	0.19	-0.17	-0.19	-0.37	0.37	-0.37	0.25	0.37	0.36	-0.36	0.36	-0.36	-0.24	-0.62	0.60
**5**	-0.18	-0.18	0.18	-0.19	0.19	0.19	0.37	-0.37	0.37	-0.25	-0.37	-0.36	0.36	-0.36	0.36	0.24	0.62	-0.60
**2**	0.19	0.19	-0.19	0.18	-0.18	-0.18	-0.36	0.36	-0.36	0.24	0.36	0.37	-0.37	0.37	-0.37	-0.25	-0.60	0.62
**4**	-0.19	-0.17	0.19	-0.18	0.18	0.18	0.36	-0.36	0.36	-0.24	-0.36	-0.37	0.37	-0.37	0.37	0.25	0.60	-0.62
**6**	-0.17	-0.19	0.19	-0.18	0.18	0.18	0.36	-0.36	0.36	-0.24	-0.36	-0.37	0.37	-0.37	0.37	0.25	0.60	-0.62
**7**	-0.37	-0.37	0.37	-0.36	0.36	0.36	0.74	-0.74	0.74	-0.49	-0.74	-0.72	0.72	-0.72	0.72	0.48	1.23	-1.20
**9**	0.37	0.37	-0.37	0.36	-0.36	-0.36	-0.74	0.74	-0.74	0.51	0.74	0.72	-0.72	0.72	-0.72	-0.48	-1.23	1.20
**11**	-0.37	-0.37	0.37	-0.36	0.36	0.36	0.74	-0.74	0.74	-0.51	-0.72	-0.72	0.72	-0.72	0.72	0.48	1.23	-1.20
**8**	0.25	0.25	-0.25	0.24	-0.24	-0.24	-0.49	0.51	-0.51	0.36	0.49	0.48	-0.48	0.48	-0.48	-0.32	-0.85	0.80
**10**	0.37	0.37	-0.37	0.36	-0.36	-0.36	-0.74	0.74	-0.72	0.49	0.74	0.72	-0.72	0.72	-0.72	-0.48	-1.23	1.20
**13**	0.36	0.36	-0.36	0.37	-0.37	-0.37	-0.72	0.72	-0.72	0.48	0.72	0.74	-0.74	0.74	-0.72	-0.51	-1.20	1.23
**15**	-0.36	-0.36	0.36	-0.37	0.37	0.37	0.72	-0.72	0.72	-0.48	-0.72	-0.74	0.74	-0.74	0.74	0.51	1.20	-1.23
**12**	0.36	0.36	-0.36	0.37	-0.37	-0.37	-0.72	0.72	-0.72	0.48	0.72	0.74	-0.74	0.74	-0.74	-0.49	-1.20	1.23
**14**	-0.36	-0.36	0.36	-0.37	0.37	0.37	0.72	-0.72	0.72	-0.48	-0.72	-0.72	0.74	-0.74	0.74	0.49	1.20	-1.23
**16**	-0.24	-0.24	0.24	-0.25	0.25	0.25	0.48	-0.48	0.48	-0.32	-0.48	-0.51	0.51	-0.49	0.49	0.36	0.80	-0.85
**17**	-0.62	-0.62	0.62	-0.60	0.60	0.60	1.23	-1.23	1.23	-0.85	-1.23	-1.20	1.20	-1.20	1.20	0.80	2.09	-2.00
**18**	0.60	0.60	-0.60	0.62	-0.62	-0.62	-1.20	1.20	-1.20	0.80	1.20	1.23	-1.23	1.23	-1.23	-0.85	-2.00	2.09

Table S8 is the M-table for the same system as Table S7 but for optimal energy ($E = 0.4455$) instead of the mid-gap energy. In this case, sites 17 and 18 have a M integral of 3.31.

Table S8: The M-table $\boldsymbol{M^p(0.4455\ )}$ at optimal energy ($\boldsymbol{E = 0.4455}$) corresponding to $\boldsymbol{H^p}$ of the parent **1-X** molecules in the presence of nitrogen heteroatoms at sites 7 & 12 and sulfur anchor atoms at sites 17 & 18. The nitrogen heteroatoms have an on-site energy $\boldsymbol{\varepsilon_N = -0.18}$ and the sulfur anchor atoms have an on-site energy $\boldsymbol{\varepsilon_S = -0.6}$.

$\boldsymbol{M^p(0.4455)}$	**1**	**3**	**5**	**2**	**4**	**6**	**7**	**9**	**11**	**8**	**10**	**13**	**15**	**12**	**14**	**16**	**17**	**18**
**1**	17.33	-11.24	-3.21	-27.41	32.42	-31.71	6.12	-9.71	15.52	8.07	-13.03	1.81	-1.14	0.72	-1.52	0.94	9.29	1.09
**3**	-11.24	17.33	-3.21	-27.41	-31.71	32.42	6.12	-9.71	15.52	8.07	-13.03	1.81	-1.14	0.72	-1.52	0.94	9.29	1.09
**5**	-3.21	-3.21	15.42	28.84	-27.41	-27.41	-6.44	10.22	-16.33	-8.49	13.71	-8.73	5.46	-3.44	7.33	-4.54	-9.77	-5.23
**2**	-27.41	-27.41	28.84	15.42	-3.21	-3.21	-3.44	5.46	-8.73	-4.54	7.33	-16.33	10.22	-6.44	13.71	-8.49	-5.23	-9.77
**4**	32.42	-31.71	-27.41	-3.21	17.33	-11.24	0.72	-1.14	1.81	0.94	-1.52	15.52	-9.71	6.12	-13.03	8.07	1.09	9.29
**6**	-31.71	32.42	-27.41	-3.21	-11.24	17.33	0.72	-1.14	1.81	0.94	-1.52	15.52	-9.71	6.12	-13.03	8.07	1.09	9.29
**7**	6.12	6.12	-6.44	-3.44	0.72	0.72	-10.70	16.99	-27.15	-14.11	22.80	3.64	-2.28	1.44	-3.06	1.89	-16.25	2.18
**9**	-9.71	-9.71	10.22	5.46	-1.14	-1.14	16.99	-3.61	13.95	-30.04	-23.20	-5.78	3.62	-2.28	4.86	-3.01	3.45	-3.46
**11**	15.52	15.52	-16.33	-8.73	1.81	1.81	-27.15	13.95	-3.92	29.63	-22.50	9.24	-5.78	3.64	-7.76	4.81	-13.34	5.53
**8**	8.07	8.07	-8.49	-4.54	0.94	0.94	-14.11	-30.04	29.63	-16.26	0.91	4.81	-3.01	1.89	-4.04	2.50	28.73	2.88
**10**	-13.03	-13.03	13.71	7.33	-1.52	-1.52	22.80	-23.20	-22.50	0.91	-12.77	-7.76	4.86	-3.06	6.52	-4.04	22.19	-4.65
**13**	1.81	1.81	-8.73	-16.33	15.52	15.52	3.64	-5.78	9.24	4.81	-7.76	-3.92	13.95	-27.15	-22.50	29.63	5.53	-13.34
**15**	-1.14	-1.14	5.46	10.22	-9.71	-9.71	-2.28	3.62	-5.78	-3.01	4.86	13.95	-3.61	16.99	-23.20	-30.04	-3.46	3.45
**12**	0.72	0.72	-3.44	-6.44	6.12	6.12	1.44	-2.28	3.64	1.89	-3.06	-27.15	16.99	-10.70	22.80	-14.11	2.18	-16.25
**14**	-1.52	-1.52	7.33	13.71	-13.03	-13.03	-3.06	4.86	-7.76	-4.04	6.52	-22.50	-23.20	22.80	-12.77	0.91	-4.65	22.19
**16**	0.94	0.94	-4.54	-8.49	8.07	8.07	1.89	-3.01	4.81	2.50	-4.04	29.63	-30.04	-14.11	0.91	-16.26	2.88	28.73
**17**	9.29	9.29	-9.77	-5.23	1.09	1.09	-16.25	3.45	-13.34	28.73	22.19	5.53	-3.46	2.18	-4.65	2.88	-52.46	3.31
**18**	1.09	1.09	-5.23	-9.77	9.29	9.29	2.18	-3.46	5.53	2.88	-4.65	-13.34	3.45	-16.25	22.19	28.73	3.31	-52.46

Table S9 is the connectivity table showing the Hamiltonian H^p of the parent **2-X** molecules in the presence of nitrogen heteroatoms at sites 7 & 12 and sulfur anchor atoms at sites 17 & 18. The nitrogen heteroatoms have a non-zero on-site energy ε_N and the sulfur anchor atoms have a non-zero on-site energy ε_S.

Table S9: Connectivity table showing the Hamiltonian $\boldsymbol{H^p}$ of the parent **2-X** molecules in the presence of nitrogen heteroatoms at sites 7 & 12 and sulfur anchor atoms at sites 17 & 18. The nitrogen heteroatoms have a non-zero on-site energy $\boldsymbol{\varepsilon_N}$ and the sulfur anchor atoms ahave a non-zero on-site energy $\boldsymbol{\varepsilon_S}$.

$\boldsymbol{H^d}$	**1**	**3**	**5**	**2**	**4**	**6**	**7**	**9**	**11**	**8**	**10**	**13**	**15**	**12**	**14**	**16**	**17**	**18**
**1**	0	0	0	-1	0	-1	0	0	0	0	0	0	0	0	0	0	0	0
**3**	0	0	0	-1	-1	0	0	0	0	0	0	0	0	0	0	0	0	0
**5**	0	0	0	0	-1	-1	0	0	0	0	0	0	0	0	0	0	0	0
**2**	-1	-1	0	0	0	0	-1	0	0	0	0	0	0	0	0	0	0	0
**4**	0	-1	-1	0	0	0	0	0	0	0	0	0	0	0	0	0	0	0
**6**	-1	0	-1	0	0	0	0	0	0	0	0	0	0	-1	0	0	0	0
**7**	0	0	0	-1	0	0	ε_N	0	-1	-1	0	0	0	0	0	0	0	0
**9**	0	0	0	0	0	0	0	0	0	-1	-1	0	0	0	0	0	0	0
**11**	0	0	0	0	0	0	-1	0	0	0	-1	0	0	0	0	0	0	0
**8**	0	0	0	0	0	0	-1	-1	0	0	0	0	0	0	0	0	0	0
**10**	0	0	0	0	0	0	0	-1	-1	0	0	0	0	0	0	0	-1	0
**13**	0	0	0	0	0	0	0	0	0	0	0	0	0	-1	-1	0	0	0
**15**	0	0	0	0	0	0	0	0	0	0	0	0	0	0	-1	-1	0	0
**12**	0	0	0	0	0	-1	0	0	0	0	0	-1	0	ε_N	0	-1	0	0
**14**	0	0	0	0	0	0	0	0	0	0	0	-1	-1	0	0	0	0	-1
**16**	0	0	0	0	0	0	0	0	0	0	0	0	-1	-1	0	0	0	0
**17**	0	0	0	0	0	0	0	0	0	0	-1	0	0	0	0	0	ε_S	0
**18**	0	0	0	0	0	0	0	0	0	0	0	0	0	0	-1	0	0	ε_S

Table S10 is the M-table $M^p(0)$ at mid-gap corresponding to H^p of the parent **2-X** molecules. The values $\varepsilon_N = -0.18$ and $\varepsilon_S = -0.6$ are assigned to on-site energies of the heteroatoms as for the **1-X** series in Table S7. In this case, sites 17 and 18 have a M integral of 0. As the mid-gap M integral is −2 for the **1-X** series at sites 17 and 18, it is expected that the conductance of **1-X** series molecules is higher than the corresponding **2-X** series molecules.

Table S10: The M-table $\boldsymbol{M^p(0)}$ at mid-gap ($\boldsymbol{E = 0}$) corresponding to $\boldsymbol{H^p}$ of the parent **2-X** molecules in the presence of nitrogen heteroatoms at sites 7 & 12 and sulfur anchor atoms at sites 17 & 18. The nitrogen heteroatoms have an on-site energy $\boldsymbol{\varepsilon_N = -0.18}$ and the sulphur anchor atoms have an on-site energy $\boldsymbol{\varepsilon_S = -0.6}$.

$\boldsymbol{M^p(0)}$	**1**	**3**	**5**	**2**	**4**	**6**	**7**	**9**	**11**	**8**	**10**	**13**	**15**	**12**	**14**	**16**	**17**	**18**
**1**	0.37	0	0	0.19	-0.2	0.19	-0.4	0.37	0.25	-0.4	0.37	0.25	0.37	-0.4	0.37	-0.4	-0.6	-0.6
**3**	0	0.37	-0.4	0.19	0.19	-0.2	-0.4	0.37	0.25	-0.4	0.37	-0.3	-0.4	0.37	-0.4	0.37	-0.6	0.62
**5**	0	-0.4	0.37	-0.2	0.19	0.19	0.37	-0.4	-0.3	0.37	-0.4	0.25	0.37	-0.4	0.37	-0.4	0.62	-0.6
**2**	0.19	0.19	-0.2	0	0	0	0	0	0	0	0	0	0	0	0	0	0	0
**4**	-0.2	0.19	0.19	0	0	0	0	0	0	0	0	0	0	0	0	0	0	0
**6**	0.19	-0.2	0.19	0	0	0	0	0	0	0	0	0	0	0	0	0	0	0
**7**	-0.4	-0.4	0.37	0	0	0	0.74	-0.7	-0.5	0.74	-0.7	0	0	0	0	0	1.23	0
**9**	0.37	0.37	-0.4	0	0	0	-0.7	0.74	0.49	-0.4	0.74	0	0	0	0	0	-1.2	0
**11**	0.25	0.25	-0.3	0	0	0	-0.5	0.49	0.96	-0.9	0.87	0	0	0	0	0	-1.5	0
**8**	-0.4	-0.4	0.37	0	0	0	0.74	-0.4	-0.9	0.74	-0.7	0	0	0	0	0	1.23	0
**10**	0.37	0.37	-0.4	0	0	0	-0.7	0.74	0.87	-0.7	0.74	0	0	0	0	0	-1.2	0
**13**	0.25	-0.3	0.25	0	0	0	0	0	0	0	0	0.96	0.49	-0.5	0.87	-0.9	0	-1.5
**15**	0.37	-0.4	0.37	0	0	0	0	0	0	0	0	0.49	0.74	-0.7	0.74	-0.4	0	-1.2
**12**	-0.4	0.37	-0.4	0	0	0	0	0	0	0	0	-0.5	-0.7	0.74	-0.7	0.74	0	1.23
**14**	0.37	-0.4	0.37	0	0	0	0	0	0	0	0	0.87	0.74	-0.7	0.74	-0.7	0	-1.2
**16**	-0.4	0.37	-0.4	0	0	0	0	0	0	0	0	-0.9	-0.4	0.74	-0.7	0.74	0	1.23
**17**	-0.6	-0.6	0.62	0	0	0	1.23	-1.2	-1.5	1.23	-1.2	0	0	0	0	0	2.69	0
**18**	-0.6	0.62	-0.6	0	0	0	0	0	0	0	0	-1.5	-1.2	1.23	-1.2	1.23	0	2.69

Table S11 is the M-table for the same system as Table S10 but for optimal energy ($E = 0.42745$) instead of the mid-gap energy. In this case, sites 17 and 18 have a M integral of −0.04. As the M integral at optimal energy for the **1-X** series is 3.31 at sites 17 and 18, it is expected that the conductance of **1-X** series molecules is higher than the corresponding **2-X** series molecules.

Table S11: The M-table $\boldsymbol{M^p}(\mathbf{0.42745})$ at optimal energy ($\boldsymbol{E} = \mathbf{0.42745}$) corresponding to $\boldsymbol{H^p}$ of the parent **2-X** molecules in the presence of nitrogen heteroatoms at sites 7 & 12 and sulfur anchor atoms at sites 17 & 18. The nitrogen heteroatoms have an on-site energy $\boldsymbol{\varepsilon_N} = -\mathbf{0.18}$ and the sulfur anchor atoms have an on-site energy $\boldsymbol{\varepsilon_S} = -\mathbf{0.6}$.

$\boldsymbol{M^p}(\mathbf{0.42745})$	**1**	**3**	**5**	**2**	**4**	**6**	**7**	**9**	**11**	**8**	**10**	**13**	**15**	**12**	**14**	**16**	**17**	**18**
**1**	0.61	-0.34	-0.34	-1.45	1.59	-1.45	0.35	-0.70	0.41	0.82	-0.52	0.41	-0.70	0.35	-0.52	0.82	0.51	0.51
**3**	-0.34	0.60	0.06	-1.36	-1.53	1.51	0.33	-0.66	0.39	0.77	-0.49	-0.43	0.73	-0.36	0.55	-0.86	0.48	-0.53
**5**	-0.34	0.06	0.60	1.51	-1.53	-1.36	-0.36	0.73	-0.43	-0.86	0.55	0.39	-0.66	0.33	-0.49	0.77	-0.53	0.48
**2**	-1.45	-1.36	1.51	0.92	-0.34	-0.30	-0.22	0.45	-0.26	-0.52	0.33	0.09	-0.15	0.07	-0.11	0.17	-0.32	0.11
**4**	1.59	-1.53	-1.53	-0.34	0.99	-0.34	0.08	-0.16	0.10	0.19	-0.12	0.10	-0.16	0.08	-0.12	0.19	0.12	0.12
**6**	-1.45	1.51	-1.36	-0.30	-0.34	0.92	0.07	-0.15	0.09	0.17	-0.11	-0.26	0.45	-0.22	0.33	-0.52	0.11	-0.32
**7**	0.35	0.33	-0.36	-0.22	0.08	0.07	-0.58	1.17	-0.69	-1.37	0.87	-0.02	0.04	-0.02	0.03	-0.04	-0.85	-0.03
**9**	-0.70	-0.66	0.73	0.45	-0.16	-0.15	1.17	-0.66	0.02	-1.18	-1.18	0.04	-0.07	0.04	-0.05	0.08	1.14	0.05
**11**	0.41	0.39	-0.43	-0.26	0.10	0.09	-0.69	0.02	-0.88	1.56	-1.57	-0.02	0.04	-0.02	0.03	-0.05	1.53	-0.03
**8**	0.82	0.77	-0.86	-0.52	0.19	0.17	-1.37	-1.18	1.56	-0.20	0.71	-0.05	0.08	-0.04	0.06	-0.10	-0.69	-0.06
**10**	-0.52	-0.49	0.55	0.33	-0.12	-0.11	0.87	-1.18	-1.57	0.71	-0.20	0.03	-0.05	0.03	-0.04	0.06	0.20	0.04
**13**	0.41	-0.43	0.39	0.09	0.10	-0.26	-0.02	0.04	-0.02	-0.05	0.03	-0.88	0.02	-0.69	-1.57	1.56	-0.03	1.53
**15**	-0.70	0.73	-0.66	-0.15	-0.16	0.45	0.04	-0.07	0.04	0.08	-0.05	0.02	-0.66	1.17	-1.18	-1.18	0.05	1.14
**12**	0.35	-0.36	0.33	0.07	0.08	-0.22	-0.02	0.04	-0.02	-0.04	0.03	-0.69	1.17	-0.58	0.87	-1.37	-0.03	-0.85
**14**	-0.52	0.55	-0.49	-0.11	-0.12	0.33	0.03	-0.05	0.03	0.06	-0.04	-1.57	-1.18	0.87	-0.20	0.71	0.04	0.20
**16**	0.82	-0.86	0.77	0.17	0.19	-0.52	-0.04	0.08	-0.05	-0.10	0.06	1.56	-1.18	-1.37	0.71	-0.20	-0.06	-0.69
**17**	0.51	0.48	-0.53	-0.32	0.12	0.11	-0.85	1.14	1.53	-0.69	0.20	-0.03	0.05	-0.03	0.04	-0.06	-2.76	-0.04
**18**	0.51	-0.53	0.48	0.11	0.12	-0.32	-0.03	0.05	-0.03	-0.06	0.04	1.53	1.14	-0.85	0.20	-0.69	-0.04	-2.76

Table S12 shows the Hamiltonian H^d of the daughter **3-X** molecules in the presence of nitrogen heteroatoms at sites 1, 7 & 12 and sulfur anchor atoms at sites 17 & 18. The nitrogen heteroatoms have a non-zero on-site energy ε_N and the sulfur anchor atoms have a non-zero on-site energy ε_S.

Table S12: The Hamiltonian $\boldsymbol{H^d}$ of the daughter **3-X** molecules in the presence of nitrogen heteroatoms at sites 1, 7 & 12 and sulfur anchor atoms at sites 17 & 18. The nitrogen heteroatoms have a non-zero on-site energy $\boldsymbol{\varepsilon_N}$ and the sulfur anchor atoms have a non-zero on-site energy $\boldsymbol{\varepsilon_S}$.

$\boldsymbol{H^d}$	**1**	**3**	**5**	**2**	**4**	**6**	**7**	**9**	**11**	**8**	**10**	**13**	**15**	**12**	**14**	**16**	**17**	**18**
**1**	ε_N	0	0	-1	0	-1	0	0	0	0	0	0	0	0	0	0	0	0
**3**	0	0	0	-1	-1	0	0	0	0	0	0	0	0	0	0	0	0	0
**5**	0	0	0	0	-1	-1	0	0	0	0	0	0	0	0	0	0	0	0
**2**	-1	-1	0	0	0	0	-1	0	0	0	0	0	0	0	0	0	0	0
**4**	0	-1	-1	0	0	0	0	0	0	0	0	0	0	0	0	0	0	0
**6**	-1	0	-1	0	0	0	0	0	0	0	0	0	0	-1	0	0	0	0
**7**	0	0	0	-1	0	0	ε_N	0	-1	-1	0	0	0	0	0	0	0	0
**9**	0	0	0	0	0	0	0	0	0	-1	-1	0	0	0	0	0	0	0
**11**	0	0	0	0	0	0	-1	0	0	0	-1	0	0	0	0	0	0	0
**8**	0	0	0	0	0	0	-1	-1	0	0	0	0	0	0	0	0	0	0
**10**	0	0	0	0	0	0	0	-1	-1	0	0	0	0	0	0	0	-1	0
**13**	0	0	0	0	0	0	0	0	0	0	0	0	0	-1	-1	0	0	0
**15**	0	0	0	0	0	0	0	0	0	0	0	0	0	0	-1	-1	0	0
**12**	0	0	0	0	0	-1	0	0	0	0	0	-1	0	ε_N	0	-1	0	0
**14**	0	0	0	0	0	0	0	0	0	0	0	-1	-1	0	0	0	0	-1
**16**	0	0	0	0	0	0	0	0	0	0	0	0	-1	-1	0	0	0	0
**17**	0	0	0	0	0	0	0	0	0	0	-1	0	0	0	0	0	ε_S	0
**18**	0	0	0	0	0	0	0	0	0	0	0	0	0	0	-1	0	0	ε_S

Table S13 is the M-table $M^d(0)$ at mid-gap corresponding to H^d of the daughter **3-X** molecules. The values $\varepsilon_N = -0.18$ and $\varepsilon_S = -0.6$ are assigned to on-site energies of the heteroatoms as for the **1-X** series in Table S7 and **2-X** series in Table S10. In this case, sites 17 and 18 have a M integral of −0.18. By comparing with the mid-gap M integrals at sites 17 and 18 for the **1-X** series (−2) and **2-X** series (0), two conductance predictions can be made: (1) The conductance of **1-X** series molecules is expected to be much higher than the corresponding **2-X** and **3-X** species, (2) The conductance of the **3-X** series molecules is expected to be slightly higher than the corresponding **2-X** species.

Table S13: The M-table $\boldsymbol{M^d(0)}$ at mid-gap ($\boldsymbol{E = 0}$) corresponding to $\boldsymbol{H^d}$ in the presence of nitrogen heteroatoms at sites 7 & 12 and sulfur anchor atoms at sites 17 & 18. The nitrogen heteroatoms have an on-site energy $\boldsymbol{\varepsilon_N = -0.18}$ and the sulfur anchor atoms have an on-site energy $\boldsymbol{\varepsilon_S = -0.6}$.

$\boldsymbol{M^d(0)}$	**1**	**3**	**5**	**2**	**4**	**6**	**7**	**9**	**11**	**8**	**10**	**13**	**15**	**12**	**14**	**16**	**17**	**18**
**1**	0.37	0	0	0.19	-0.19	0.19	-0.37	0.37	0.25	-0.37	0.37	0.25	0.37	-0.37	0.37	-0.37	-0.62	-0.62
**3**	0	0.43	-0.43	0.22	0.22	-0.22	-0.43	0.43	0.29	-0.43	0.43	-0.29	-0.43	0.43	-0.43	0.43	-0.72	0.72
**5**	0	-0.43	0.43	-0.22	0.22	0.22	0.43	-0.43	-0.29	0.43	-0.43	0.29	0.43	-0.43	0.43	-0.43	0.72	-0.72
**2**	0.19	0.22	-0.22	-0.02	0.02	-0.02	0.03	-0.03	-0.02	0.03	-0.03	-0.02	-0.03	0.03	-0.03	0.03	0.06	0.06
**4**	-0.19	0.22	0.22	0.02	-0.02	0.02	-0.03	0.03	0.02	-0.03	0.03	0.02	0.03	-0.03	0.03	-0.03	-0.06	-0.06
**6**	0.19	-0.22	0.22	-0.02	0.02	-0.02	0.03	-0.03	-0.02	0.03	-0.03	-0.02	-0.03	0.03	-0.03	0.03	0.06	0.06
**7**	-0.37	-0.43	0.43	0.03	-0.03	0.03	0.80	-0.80	-0.54	0.80	-0.80	0.04	0.06	-0.06	0.06	-0.06	1.34	-0.11
**9**	0.37	0.43	-0.43	-0.03	0.03	-0.03	-0.80	0.80	0.54	-0.36	0.80	-0.04	-0.06	0.06	-0.06	0.06	-1.34	0.11
**11**	0.25	0.29	-0.29	-0.02	0.02	-0.02	-0.54	0.54	1.10	-0.98	0.98	-0.03	-0.04	0.04	-0.04	0.04	-1.64	0.07
**8**	-0.37	-0.43	0.43	0.03	-0.03	0.03	0.80	-0.36	-0.98	0.80	-0.80	0.04	0.06	-0.06	0.06	-0.06	1.34	-0.11
**10**	0.37	0.43	-0.43	-0.03	0.03	-0.03	-0.80	0.80	0.98	-0.80	0.80	-0.04	-0.06	0.06	-0.06	0.06	-1.34	0.11
**13**	0.25	-0.29	0.29	-0.02	0.02	-0.02	0.04	-0.04	-0.03	0.04	-0.04	1.10	0.54	-0.54	0.98	-0.98	0.07	-1.64
**15**	0.37	-0.43	0.43	-0.03	0.03	-0.03	0.06	-0.06	-0.04	0.06	-0.06	0.54	0.80	-0.80	0.80	-0.36	0.11	-1.34
**12**	-0.37	0.43	-0.43	0.03	-0.03	0.03	-0.06	0.06	0.04	-0.06	0.06	-0.54	-0.80	0.80	-0.80	0.80	-0.11	1.34
**14**	0.37	-0.43	0.43	-0.03	0.03	-0.03	0.06	-0.06	-0.04	0.06	-0.06	0.98	0.80	-0.80	0.80	-0.80	0.11	-1.34
**16**	-0.37	0.43	-0.43	0.03	-0.03	0.03	-0.06	0.06	0.04	-0.06	0.06	-0.98	-0.36	0.80	-0.80	0.80	-0.11	1.34
**17**	-0.62	-0.72	0.72	0.06	-0.06	0.06	1.34	-1.34	-1.64	1.34	-1.34	0.07	0.11	-0.11	0.11	-0.11	2.98	-0.18
**18**	-0.62	0.72	-0.72	0.06	-0.06	0.06	-0.11	0.11	0.07	-0.11	0.11	-1.64	-1.34	1.34	-1.34	1.34	-0.18	2.98

Table S14 is the M-table for the same system as Table S13 but for optimal energy ($E = 0.42745$) instead of the mid-gap energy. In this case, sites 17 and 18 have an M integral of −0.02. By comparing with the optimal energy M integrals at sites 17 and 18 for the **1-X** series (3.31) and **2-X** series (−0.04), two conductance predictions can be made: (1) The conductance of **1-X** series molecules is expected to be much higher than the corresponding **2-X** and **3-X** species, (2) The conductance of **3-X** series molecules is expected to be almost identical to the corresponding **2-X** species.

Table S14: The M-table $\boldsymbol{M^d}(\boldsymbol{E} = \mathbf{0.42745})$ at optimal energy ($\boldsymbol{E} = \mathbf{0.42745}$) corresponding to $\boldsymbol{H^d}$ in the presence of nitrogen heteroatoms at sites 7 & 12 and sulfur anchor atoms at sites 17 & 18. The nitrogen heteroatoms have an on-site energy $\boldsymbol{\varepsilon_N} = -\mathbf{0.18}$ and the sulfur anchor atoms have an on-site energy $\boldsymbol{\varepsilon_S} = -\mathbf{0.6}$.

$\boldsymbol{M^d}(\mathbf{0.42745})$	**1**	**3**	**5**	**2**	**4**	**6**	**7**	**9**	**11**	**8**	**10**	**13**	**15**	**12**	**14**	**16**	**17**	**18**
**1**	0.53	-0.30	-0.30	-1.28	1.41	-1.28	0.31	-0.62	0.36	0.73	-0.47	0.36	-0.62	0.31	-0.47	0.73	0.46	0.46
**3**	-0.30	0.50	0.06	-1.12	-1.33	1.30	0.27	-0.54	0.32	0.64	-0.41	-0.37	0.63	-0.32	0.47	-0.74	0.40	-0.46
**5**	-0.30	0.06	0.50	1.30	-1.33	-1.12	-0.32	0.63	-0.37	-0.74	0.47	0.32	-0.54	0.27	-0.41	0.64	-0.46	0.40
**2**	-1.28	-1.12	1.30	0.90	-0.42	-0.13	-0.22	0.44	-0.26	-0.51	0.33	0.04	-0.06	0.03	-0.05	0.07	-0.32	0.05
**4**	1.41	-1.33	-1.33	-0.42	0.99	-0.42	0.10	-0.21	0.12	0.24	-0.15	0.12	-0.21	0.10	-0.15	0.24	0.15	0.15
**6**	-1.28	1.30	-1.12	-0.13	-0.42	0.90	0.03	-0.06	0.04	0.07	-0.05	-0.26	0.44	-0.22	0.33	-0.51	0.05	-0.32
**7**	0.31	0.27	-0.32	-0.22	0.10	0.03	-0.49	0.98	-0.57	-1.15	0.74	-0.01	0.02	-0.01	0.01	-0.02	-0.72	-0.01
**9**	-0.62	-0.54	0.63	0.44	-0.21	-0.06	0.98	-0.54	0.00	-1.03	-0.98	0.02	-0.03	0.02	-0.02	0.04	0.96	0.02
**11**	0.36	0.32	-0.37	-0.26	0.12	0.04	-0.57	0.00	-0.75	1.35	-1.35	-0.01	0.02	-0.01	0.01	-0.02	1.32	-0.01
**8**	0.73	0.64	-0.74	-0.51	0.24	0.07	-1.15	-1.03	1.35	-0.14	0.58	-0.02	0.04	-0.02	0.03	-0.04	-0.57	-0.03
**10**	-0.47	-0.41	0.47	0.33	-0.15	-0.05	0.74	-0.98	-1.35	0.58	-0.16	0.01	-0.02	0.01	-0.02	0.03	0.16	0.02
**13**	0.36	-0.37	0.32	0.04	0.12	-0.26	-0.01	0.02	-0.01	-0.02	0.01	-0.75	0.00	-0.57	-1.35	1.35	-0.01	1.32
**15**	-0.62	0.63	-0.54	-0.06	-0.21	0.44	0.02	-0.03	0.02	0.04	-0.02	0.00	-0.54	0.98	-0.98	-1.03	0.02	0.96
**12**	0.31	-0.32	0.27	0.03	0.10	-0.22	-0.01	0.02	-0.01	-0.02	0.01	-0.57	0.98	-0.49	0.74	-1.15	-0.01	-0.72
**14**	-0.47	0.47	-0.41	-0.05	-0.15	0.33	0.01	-0.02	0.01	0.03	-0.02	-1.35	-0.98	0.74	-0.16	0.58	0.02	0.16
**16**	0.73	-0.74	0.64	0.07	0.24	-0.51	-0.02	0.04	-0.02	-0.04	0.03	1.35	-1.03	-1.15	0.58	-0.14	-0.03	-0.57
**17**	0.46	0.40	-0.46	-0.32	0.15	0.05	-0.72	0.96	1.32	-0.57	0.16	-0.01	0.02	-0.01	0.02	-0.03	-2.35	-0.02
**18**	0.46	-0.46	0.40	0.05	0.15	-0.32	-0.01	0.02	-0.01	-0.03	0.02	1.32	0.96	-0.72	0.16	-0.57	-0.02	-2.35

The predictions of M-theory on the full molecules, working at the mid-gap energy, do not differ from those afforded by analyzing only the molecular cores in Section 4.2.2. Working instead at the optimal energy of each system, the predictions are: (a) **1-R** series parent molecules are expected to have a higher conductance than both the corresponding **2-R** series parent and **3-R** series daughter molecules (in agreement with the simpler core-only model and the mid-gap calculations), (b) **3-R** series daughter molecules are expected to have very similar conductance to the corresponding **2-R** series parent molecules (in contrast to the other models, where the **3-R** series are expected to be more conductive than the **2-R** series), (c) the modulus of the optimal energy M integral for the different series follows the trend:

1-R (3.31) >> **2-R** (0.04) ≈ **3-R** (0.02)

The implication is that for the **1-R** series CQI can be expected whereas DQI can be expected for the **2-R** and **3-R** series.

4.3: Geometry optimization

The optimized molecular geometries and corresponding ground-state Hamiltonian matrices were self-consistently obtained using the SIESTA[25] implementation of DFT using standard norm-conserving pseudopotentials. We utilized the exchange and correlation functionals generalized gradient approximation (GGA) with the Perdew-Burke-Ernzerhof (PBE) parameterization, a double-zeta polarized basis set, and a real space grid defined with an equivalent energy cutoff of 250 Rydberg. Geometry optimization for each structure was performed for the force of 0.01 $eV\,\text{Å}^{-1}$ and the temperature was set at 300 K (26.85 °C).

4.4: Electron transport

A typical molecular junction system consists of left (source) and right (drain) leads and the scattering region. To calculate the system's phase coherent and elastic scattering properties, from the converged DFT calculations, the underlying mean-field Hamiltonian matrices were combined with an implementation of the non-equilibrium Green's function method, GOLLUM.[20, 26] The transmission coefficient $T(E)$ for electrons of energy E passing from the left lead to the right lead can be calculated using Equation (4):

$$T(E) = trace\left(\Gamma_R(E)G^R(E)\Gamma_L(E)G^{R\dagger}(E)\right) \qquad (5)$$

where:
$\Gamma_{L,R}(E) = i\left(\sum_{L,R}(E) - \sum_{L,R}^{\dagger}(E)\right)$ describes the level broadening due to the coupling between the left (L) and right (R) electrodes and the central scattering region,
$\sum_{L,R}(E)$ are the retarded self-energies associated with the coupling,
$G^R = (ES - H - \Sigma_L - \Sigma_R)^{-1}$ is the retarded Green's function,
H is the Hamiltonian,
S is the overlap matrix.

Once $T(E)$ is obtained from Equation (5), the electrical current I and conductance G can be calculated by the Landauer formula,[27] which describes the phenomenon of coherent transport in molecular junctions:

$$I = \frac{e}{h}\int dE\, T(E)\,\left(f\left(E + \frac{eV}{2}\right) - f\left(E - \frac{eV}{2}\right)\right)$$

$$G = G_0 \int dE\, T(E)\left(-\frac{\partial f}{\partial E}\right) \qquad (6)$$

where:
e is the electron charge,
h is Planck's constant,

$G_0 = 2e^2/h$ is the conductance quantum,
V is the bias voltage,
$f(E) = \left(1 + \exp\left(\frac{(E-E_F)}{k_B T}\right)\right)^{-1}$ is the Fermi distribution function,
$k_B = 8.6 \times 10^{-5}\ eV/K$ is the Boltzmann's constant,
T is the temperature.

At $T = 0\ K$, Equation (5) simplifies to:

$$G = G_0 T(E_F) \tag{7}$$

4.5: Junction Schematics

Figure S31 shows the DFT-optimized junction schematics of all molecules between two gold electrodes used for electron transport calculations. Electron transport is sensitive to configurations such as a gold electrode connected with a 'floppy' SMe anchor group.[28] However, for this work, the priority was understanding the effect of changes in the molecular core. Hence, it was crucial to minimize the effect of anchors on transport. Instead of changing the anchor group, the alternative was to find the ground state geometry and the gold-SMe configuration for one of the bridges. The SMe anchors have been made part of the gold electrodes to address the floppiness. The optimized distance between the gold electrode tip and the sulfur atom in the anchor is 0.266 nm. The same gold-SMe configuration was used for all other structures (by fixing the geometry of the gold to the SMe and relaxing the bridge) to rule out the effect of the anchor on transport for different bridges. With this method, a fair comparison between different molecules could be carried out, and the effect of the molecular core on transport could be further understood. For the gold-thiolate configurations, the methyl groups were removed from the SMe anchors and then the resulting molecule was relaxed between the gold leads.

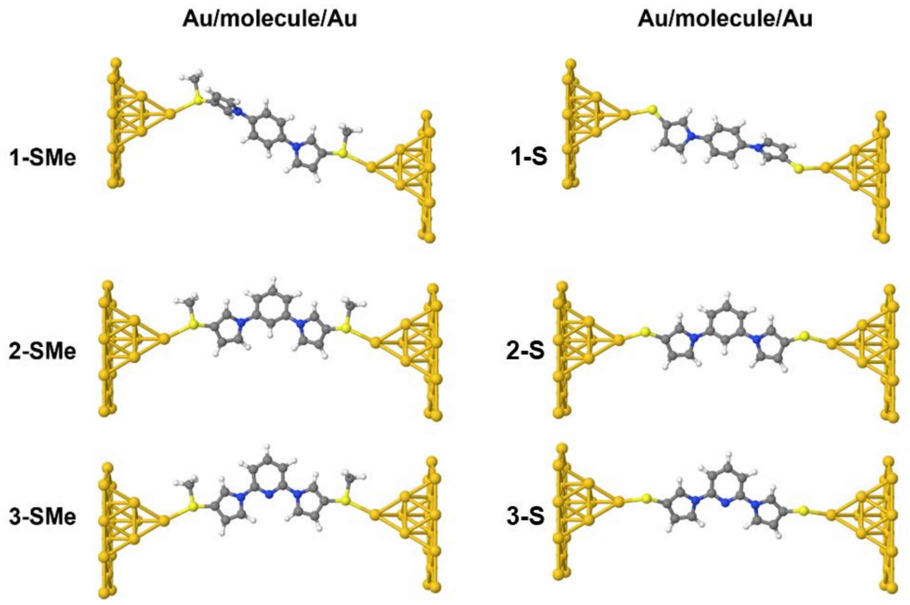

Figure S31: DFT-optimized configurations of all molecules between two gold electrodes for electron transport calculations. Calculated Au---Au distances are: **1-SMe** 1.765 nm; **2-SMe** 1.720 nm; **3-SMe** 1.724 nm; **1-S** 1.762 nm; **2-S** 1.722 nm; **3-S** 1.725 nm.

4.6: Local density of states

Analyzing the local density of states (LDOS) allowed the exact HOMO-LUMO resonances in the $T(E)$ plots to be pinpointed. Figure S32 shows the LDOS for **1-SMe**, **2-SMe** and **3-SMe**.

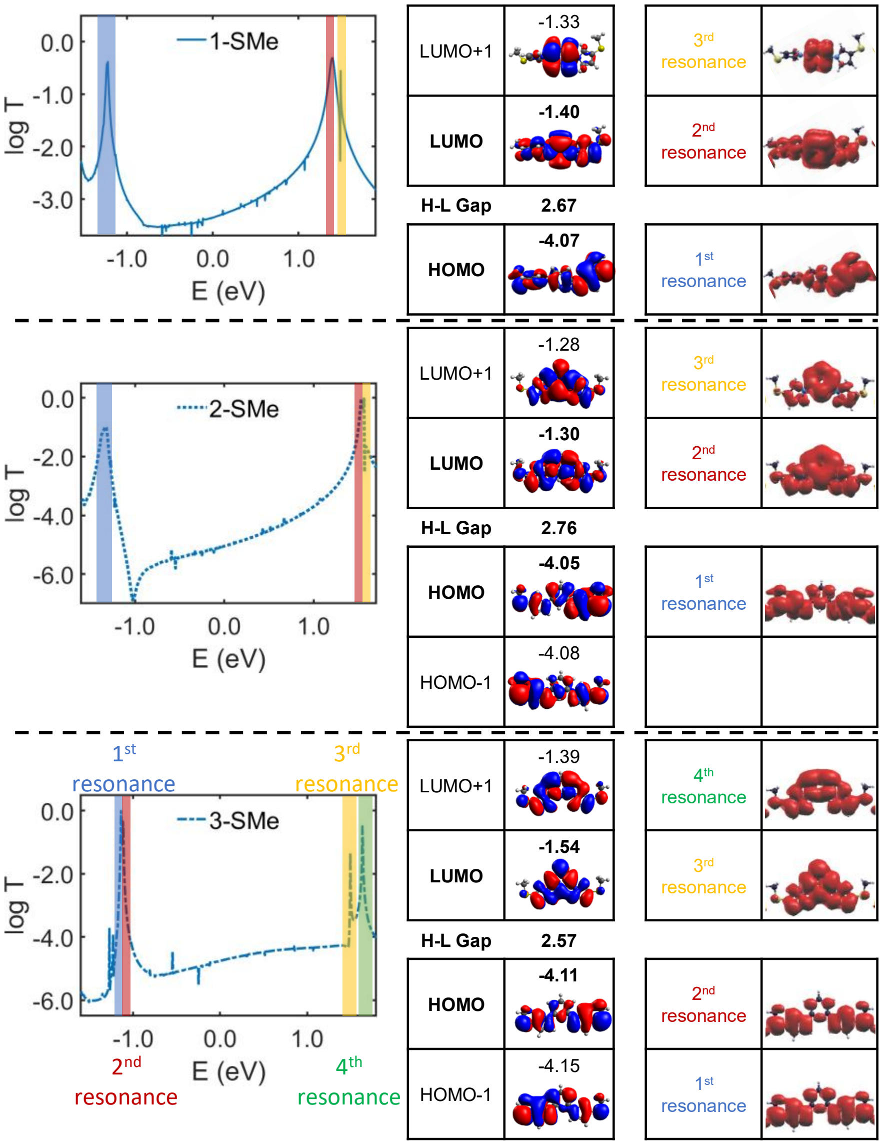

Figure S32: LDOS for **1-SMe, 2-SMe**, and **3-SMe**.

As there is no degeneracy for **1-SMe**, there is an excellent agreement between LDOS and DFT orbitals confirming the 1st and 2nd resonances are the HOMO and LUMO resonances, respectively. A CQI feature is observed between the HOMO and LUMO resonances for **1-SMe**. For **2-SMe**, due to degeneracy of both the HOMO and LUMO, the LDOS appears to be hybridized. Hence, the LDOS for the 1st resonance looks like a combination of the HOMO and HOMO-1 while the LDOS for both the 2nd and 3rd resonances (which lie very close to one another in energy) resembles a combination of the LUMO and LUMO+1. A DQI feature is observed between the HOMO (1st) and LUMO (2nd) resonances for **2-SMe**, closer in energy to the HOMO resonance (as this lies far from E_F and the center of the HOMO-LUMO gap, this can be assigned as SDQI). For **3-SMe**, due to degeneracy of the HOMO side, the corresponding LDOS also appears to be hybridized, i.e. the LDOS for the 1st and 2nd resonances looks like a combination of the HOMO and HOMO-1 orbitals. There is no sharp DQI dip between the

HOMO (2nd) and LUMO (3rd) resonances, but the shape of the curve is not characteristic of CQI, with a sharp change in transmission close to the LUMO and possible weak DQI feature close to the HOMO. LDOS analysis for **1-S**, **2-S** and **3-S** is shown in Figure S33.

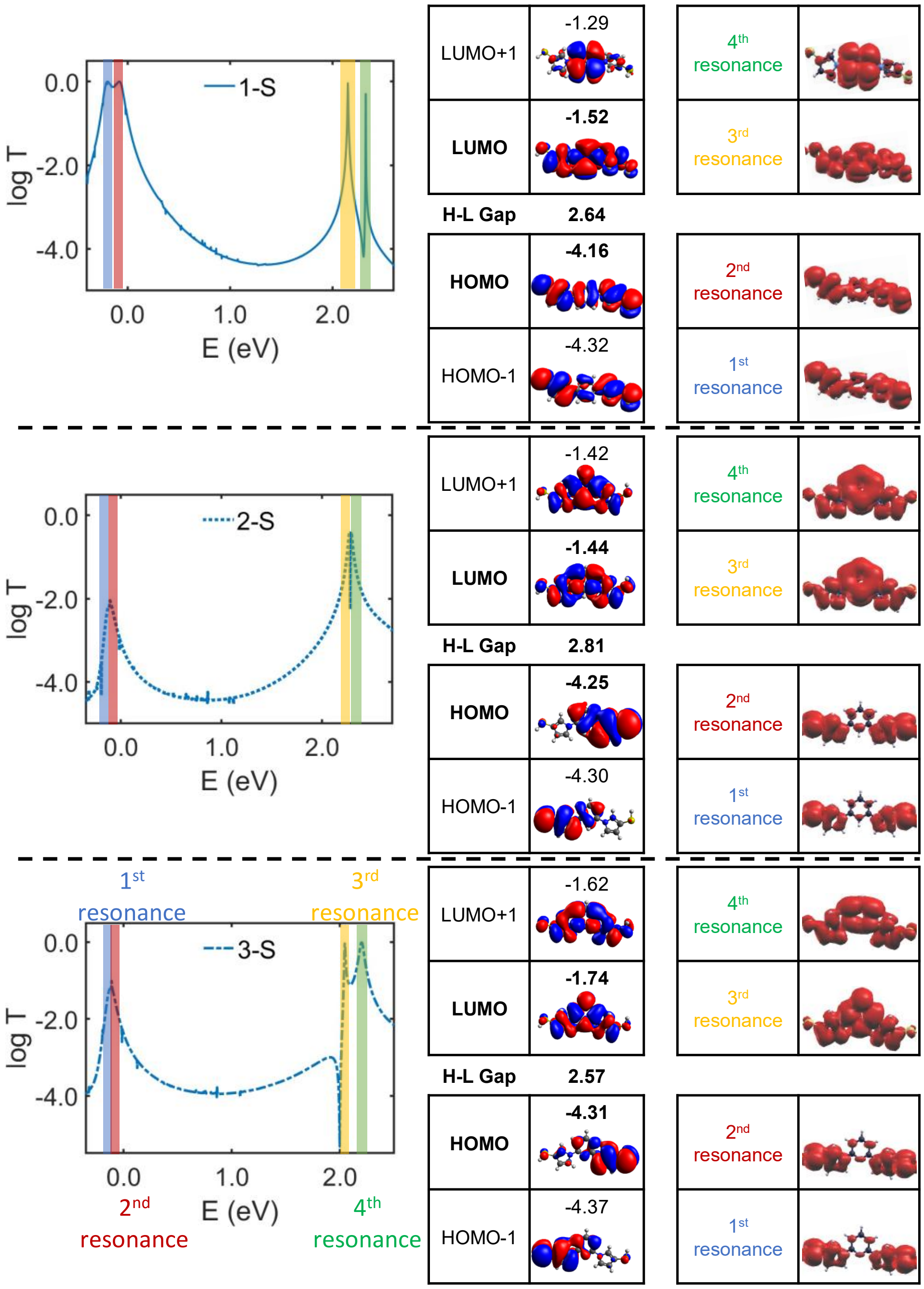

Figure S33: LDOS for **1-S, 2-S**, and **3-S**.

For **1-S**, there is no degeneracy and a CQI feature is observed between the HOMO (2nd) and LUMO (3rd) resonances. For **2-S**, a CQI feature is seen between the HOMO (2nd) and LUMO (3rd) resonances. As discussed in detail in Section 4.1.2, the form of this curve is attributed to washing out of an antiresonance close to the LUMO, and CQI is expected for **2-S**. For **3-S**, due to degeneracy of the HOMO side, the corresponding LDOS also appears to be hybridized, i.e. the LDOS for the 1st and 2nd resonances looks like a combination of the HOMO and HOMO-1 orbitals. A clear DQI feature is observed between the HOMO (2nd) and LUMO (3rd) resonances for **3-S**, much closer in energy to the LUMO resonance (as this lies far from E_F and the center of the HOMO-LUMO gap, this can be assigned as SDQI).

4.6: DFT & TB Transmission Plots

Panels a-c of Figure S34 show the DFT electron transmission $T(E)$ plots where the area under the curve is current. Figure S34a indicates CQI for both **1-SMe** and **1-S** as no antiresonances are apparent. Figure S34b indicates DQI for **2-SMe** due to an antiresonance in the HOMO-LUMO gap (resembling SDQI as classified by ECARs[1] as this lies far from E_F and the center of the HOMO-LUMO gap) but this is not seen for **2-S**, indicative of CQI or an antiresonance shifted beyond the HOMO-LUMO gap. Figure S34c indicates DQI for both **3-SMe** and **3-S**, with both curves showing features close to the LUMO (again resembling SDQI as classified by ECARs). **1-SMe** and **1-S** show higher transmission than **2-SMe** and **2-S**, respectively, at E_F, which is expected as *para* connectivity generally shows higher transmission than *meta* connectivity.

Two simple tight-binding (TB) models, denoted **I** (see Figure S34d) and **II** (see Figure S34e), respectively, were used to validate the DFT $T(E)$ plots. TB model **II** resembles the real system much more closely as it considered the effect of the nitrogen heteroatoms present in the leads. Hence, the TB results from model **II** are the focus of discussion in the main text and are also shown in Figure 4. Both TB models utilized on-site energy $\varepsilon_0 = 0$ and hopping integral $\gamma = -1$, with the on-site energy of the nitrogen atoms $\varepsilon_N = -0.45$. Both models indicate CQI for molecular core **1** and DQI for molecular core **2**. A fairly weak SDQI feature is seen for molecular core **3** in model **I** but is unclear using model **II**, where the effect of the pyrrole nitrogen atoms is accounted for. While the shorter C–N bond increases electronic coupling by ~10%, this variation does not significantly alter the TB model's trends. To maintain a minimalistic model that highlights the essential physics, we focus on the on-site energy shifts of the heteroatoms. Modifying only the on-site energy of N relative to C provides sufficient qualitative agreement with DFT calculations, confirming that the heteroatom on-site energy is the primary driver of the observed behavior.

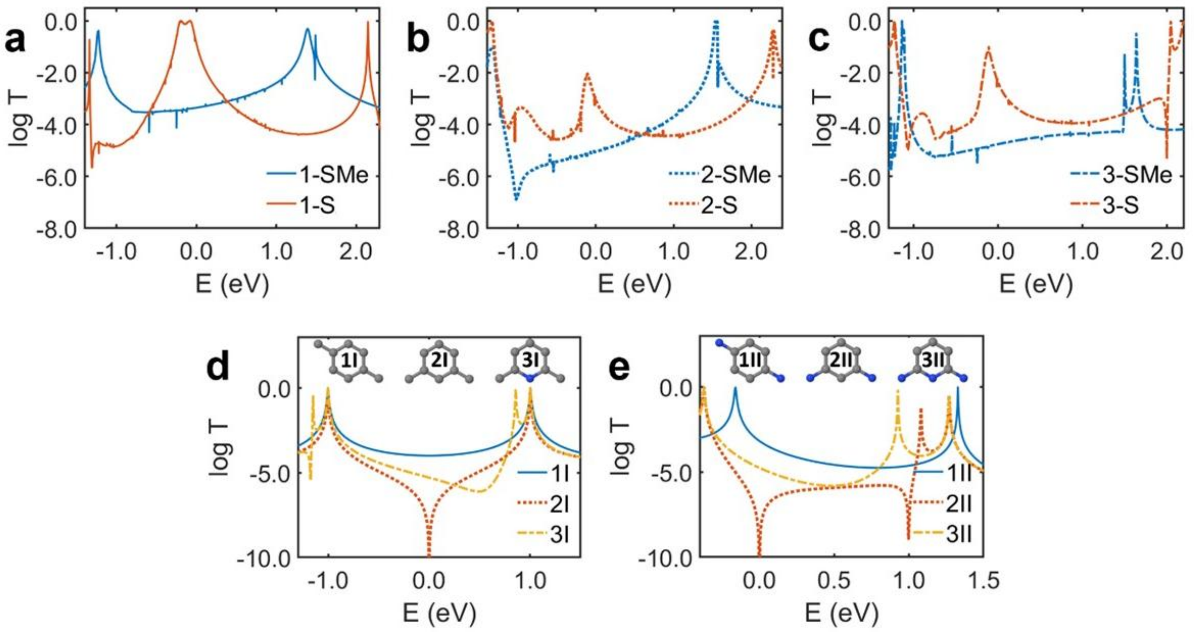

Figure S34: Top: Transmission coefficients for electrons passing through all molecules between gold electrodes using DFT material-specific Hamiltonians: (a) **1-SMe** and **1-S**, (b) **2-SMe** and **2-S**, (c) **3-SMe** and **3-S**. $\boldsymbol{E = 0}$ is DFT Fermi energy. Bottom: Simple TB models with all non-nitrogen on-site energies and couplings as $\boldsymbol{\varepsilon_0 = 0}$ and $\boldsymbol{\gamma = -1}$, respectively and on-site energy of nitrogen atoms $\boldsymbol{\varepsilon_N = -0.45}$: (d) TB model I for molecular cores **1**-**3**, (e) TB model II for molecular cores **1**-**3**.

5. SUMMARY

Table S15 summarizes the observed experimental and theoretical conductance trends, and the QI behavior predicted by different methods.

Table S15: Summary of conductance trends and predicted QI behavior.

	**Predictive, computational or experimental method**	**1-SMe**	**2-SMe**	**3-SMe**	**1-S**	**2-S**	**3-S**
Conductance	Experimental conductance (STM-BJ)	$G_{1\text{-SMe}} > G_{2\text{-SMe}} \approx G_{3\text{-SMe}}$			$G_{1\text{-S}} > G_{2\text{-S}} \approx G_{3\text{-S}}$		
	M-theory (core)[a]	$G_1 >> G_3 > G_2$					
	M-theory (core+S,[b] mid-gap)	$G_1 >> G_3 > G_2$					
	M-theory (core+S,[b] optimal energy)	$G_1 >> G_2 \approx G_3$					
	DFT conductance at E_F = 0.5 eV	$G_{1\text{-SMe}} > G_{2\text{-SMe}} \approx G_{3\text{-SMe}}$			$G_{1\text{-S}} > G_{3\text{-S}} > G_{2\text{-S}}$		
QI Behavior	ECARs[a,c]	DQI	DQI	DQI	DQI	DQI	DQI
	Orbital analysis (bridge only)[a]	CQI	Ambi-guous[d]	Ambi-guous[d]	CQI	Ambi-guous[d]	Ambi-guous[d]
	Orbital analysis (bridge + anchor)	CQI	Ambi-guous[d]	Ambi-guous[d]	CQI[e]	Ambi-guous[d,e]	Ambi-guous[d,e]
	TB – model **I**[a]	CQI	DQI	SDQI	CQI	DQI	SDQI
	TB – model **II**[a]	CQI	DQI	CQI?	CQI	DQI	CQI?
	DFT $T(E)$ (based on transmission curve in HOMO-LUMO gap)	CQI	SDQI	SDQI?	CQI	CQI	SDQI

[a] Accounts for molecular core **1**, **2** or **3** only – no anchoring group effects; [b] Accounts for the sulfur atom of the anchoring group, but does not distinguish between thiolate (**n-S**) and thiomethyl (**n-SMe**); [c] See Section 1 above for discussion of how the ECARs method could be amended to account for the observed experimental results, noting that further empirical observations are required to confirm this; [d] QI prediction dependent on how degeneracy is accounted for; [e] orbitals modelled for neutral thiol derivatives **(n-SH)** rather than gold-bound thiolates (**n-S**).

6. REFERENCES

(1) O'Driscoll, L. J.; Bryce, M. R. Extended curly arrow rules to rationalise and predict structural effects on quantum interference in molecular junctions. *Nanoscale* **2021**, *13* (2), 1103-1123.

(2) Leary, E.; Roldán-Piñero, C.; Rico-Sánchez-Mateos, R.; Zotti, L. A. Antiaromatic non-alternant heterocyclic compounds as molecular wires. *J. Mater. Chem. C* **2024**, *12* (12), 4306-4315.

(3) Liu, X.; Sangtarash, S.; Reber, D.; Zhang, D.; Sadeghi, H.; Shi, J.; Xiao, Z.-Y.; Hong, W.; Lambert, C. J.; Liu, S.-X. Gating of Quantum Interference in Molecular Junctions by Heteroatom Substitution. *Angewandte Chemie International Edition* **2017**, *56* (1), 173-176.

(4) Tang, Y.; Zhou, Y.; Zhou, D.; Chen, Y.; Xiao, Z.; Shi, J.; Liu, J.; Hong, W. Electric Field-Induced Assembly in Single-Stacking Terphenyl Junctions. *Journal of the American Chemical Society* **2020**, *142* (45), 19101-19109.

(5) Qu, F.-Y.; Zhao, Z.-H.; Ren, X.-R.; Zhang, S.-F.; Wang, L.; Wang, D. Multiple heteroatom substitution effect on destructive quantum interference in tripodal single-molecule junctions. *Phys. Chem. Chem. Phys.* **2022**, *24* (43), 26795-26801.

(6) Miao, R.; Xu, H.; Skripnik, M.; Cui, L.; Wang, K.; Pedersen, K. G. L.; Leijnse, M.; Pauly, F.; Wärnmark, K.; Meyhofer, E.; Reddy, P.; Linke, H. Influence of Quantum Interference on the Thermoelectric Properties of Molecular Junctions. *Nano Lett.* **2018**, *18* (9), 5666-5672.

(7) Manrique, D. Z.; Huang, C.; Baghernejad, M.; Zhao, X.; Al-Owaedi, O. A.; Sadeghi, H.; Kaliginedi, V.; Hong, W.; Gulcur, M.; Wandlowski, T.; Bryce, M. R.; Lambert, C. J. A quantum circuit rule for interference effects in single-molecule electrical junctions. *Nat. Commun.* **2015**, *6* (1), 6389.

(8) Huang, B.; Liu, X.; Yuan, Y.; Hong, Z.-W.; Zheng, J.-F.; Pei, L.-Q.; Shao, Y.; Li, J.-F.; Zhou, X.-S.; Chen, J.-Z.; et al. Controlling and Observing Sharp-Valleyed Quantum Interference Effect in Single Molecular Junctions. *Journal of the American Chemical Society* **2018**, *140* (50), 17685-17690.

(9) Li, Y.; Buerkle, M.; Li, G.; Rostamian, A.; Wang, H.; Wang, Z.; Bowler, D. R.; Miyazaki, T.; Xiang, L.; Asai, Y.; Zhou, G.; Tao, N. Gate controlling of quantum interference and direct observation of anti-resonances in single molecule charge transport. *Nat. Mater.* **2019**, *18* (4), 357-363.

(10) Li, S.; Yu, H.; Schwieter, K.; Chen, K.; Li, B.; Liu, Y.; Moore, J. S.; Schroeder, C. M. Charge Transport and Quantum Interference Effects in Oxazole-Terminated Conjugated Oligomers. *J. Am. Chem. Soc.* **2019**, *141* (40), 16079-16084.

(11) O'Driscoll, L. J.; Sangtarash, S.; Xu, W.; Daaoub, A.; Hong, W.; Sadeghi, H.; Bryce, M. R. Heteroatom Effects on Quantum Interference in Molecular Junctions: Modulating Antiresonances by Molecular Design. *J. Phys. Chem. C* **2021**, *125* (31), 17385-17391.

(12) Gottlieb, H. E.; Kotlyar, V.; Nudelman, A. NMR Chemical Shifts of Common Laboratory Solvents as Trace Impurities. *J. Org. Chem.* **1997**, *62* (21), 7512-7515.

(13) O'Driscoll, L. J.; Hamill, J. M.; Grace, I.; Nielsen, B. W.; Almutib, E.; Fu, Y.; Hong, W.; Lambert, C. J.; Jeppesen, J. O. Electrochemical control of the single molecule conductance of a conjugated bis(pyrrolo)tetrathiafulvalene based molecular switch. *Chem. Sci.* **2017**, *8* (9), 6123-6130.

(14) Antilla, J. C.; Baskin, J. M.; Barder, T. E.; Buchwald, S. L. Copper−Diamine-Catalyzed N-Arylation of Pyrroles, Pyrazoles, Indazoles, Imidazoles, and Triazoles. *J. Org. Chem.* **2004**, *69* (17), 5578-5587.

(15) Bray, B. L.; Mathies, P. H.; Naef, R.; Solas, D. R.; Tidwell, T. T.; Artis, D. R.; Muchowski, J. M. N-(Triisopropylsilyl)pyrrole. A progenitor "par excellence" of 3-substituted pyrroles. *J. Org. Chem.* **1990**, *55* (26), 6317-6328.

(16) Leroy, J.; Porhiel, E.; Bondon, A. Synthesis and characterization of partially β-fluorinated 5,10,15,20-tetraphenylporphyrins and some derivatives. *Tetrahedron* **2002**, *58* (33), 6713-6722.

(17) Hong, W.; Manrique, D. Z.; Moreno-García, P.; Gulcur, M.; Mishchenko, A.; Lambert, C. J.; Bryce, M. R.; Wandlowski, T. Single Molecular Conductance of Tolanes: Experimental and Theoretical Study on the Junction Evolution Dependent on the Anchoring Group. *Journal of the American Chemical Society* **2012**, *134* (4), 2292-2304.

(18) Tang, C.; Tang, Y.; Ye, Y.; Yan, Z.; Chen, Z.; Chen, L.; Zhang, L.; Liu, J.; Shi, J.; Xia, H.; Hong, W, Identifying the Conformational Isomers of Single-Molecule Cyclohexane at Room Temperature. *Chem* **2020**, *6* (10), 2770-2781.

(19) Adak, O.; Rosenthal, E.; Meisner, J.; Andrade, E. F.; Pasupathy, A. N.; Nuckolls, C.; Hybertsen, M. S.; Venkataraman, L. Flicker Noise as a Probe of Electronic Interaction at Metal–Single Molecule Interfaces. *Nano Lett.* **2015**, *15* (6), 4143-4149.

(20) Sadeghi, H. Theory of electron, phonon and spin transport in nanoscale quantum devices. *Nanotechnology* **2018**, *29* (37), 373001.

(21) Geng, Y.; Sangtarash, S.; Huang, C.; Sadeghi, H.; Fu, Y.; Hong, W.; Wandlowski, T.; Decurtins, S.; Lambert, C. J.; Liu, S.-X. Magic Ratios for Connectivity-Driven Electrical Conductance of Graphene-like Molecules. *Journal of the American Chemical Society* **2015**, *137* (13), 4469-4476.
(22) Sangtarash, S.; Huang, C.; Sadeghi, H.; Sorohhov, G.; Hauser, J.; Wandlowski, T.; Hong, W.; Decurtins, S.; Liu, S.-X.; Lambert, C. J. Searching the Hearts of Graphene-like Molecules for Simplicity, Sensitivity, and Logic. *Journal of the American Chemical Society* **2015**, *137* (35), 11425-11431.
(23) Sangtarash, S.; Sadeghi, H.; Lambert, C. J. Exploring quantum interference in heteroatom-substituted graphene-like molecules. *Nanoscale* **2016**, *8* (27), 13199-13205.
(24) Chen, H.; Chen, Y.; Zhang, H.; Cao, W.; Fang, C.; Zhou, Y.; Xiao, Z.; Shi, J.; Chen, W.; Liu, J.; Hong, W. Quantum interference enhanced thermopower in single-molecule thiophene junctions. *Chin. Chem. Lett.* **2022**, *33* (1), 523-526.
(25) Soler, J. M.; Artacho, E.; Gale, J. D.; García, A.; Junquera, J.; Ordejón, P.; Sánchez-Portal, D. The SIESTA method for ab initio order-N materials simulation. *J. Phys.: Condens. Matter* **2002**, *14* (11), 2745.
(26) Ferrer, J.; Lambert, C. J.; García-Suárez, V. M.; Manrique, D. Z.; Visontai, D.; Oroszlany, L.; Rodríguez-Ferradás, R.; Grace, I.; Bailey, S. W. D.; Gillemot, K.; Sadeghi, H.; Algharagholy, L. A. GOLLUM: a next-generation simulation tool for electron, thermal and spin transport. *New J. Phys.* **2014**, *16* (9), 093029.
(27) Landauer, R. Spatial Variation of Currents and Fields Due to Localized Scatterers in Metallic Conduction. *IBM Journal of Research and Development* **1957**, *1* (3), 223-231.
(28) Daaoub, A.; Ornago, L.; Vogel, D.; Bastante, P.; Sangtarash, S.; Parmeggiani, M.; Kamer, J.; Agraït, N.; Mayor, M.; van der Zant, H.; Sadeghi, H. Engineering Transport Orbitals in Single-Molecule Junctions. *J. Phys. Chem. Lett.* **2022**, *13* (39), 9156-9164.
